# Immune correlates analysis of antibody responses against SARS-CoV-2 variants in the ENSEMBLE vaccine efficacy trial

**DOI:** 10.1016/j.isci.2025.113660

**Published:** 2025-09-29

**Authors:** Alex Luedtke, Youyi Fong, Lars van der Laan, Fei Heng, Ying Huang, Yiwen Lu, Chenchen Yu, Lindsay N. Carpp, Sanne Roels, Mathieu Le Gars, Griet A. Van Roey, Daniel J. Stieh, Ilse Van Dromme, Avi Kenny, Marco Carone, Ollivier Hyrien, Victor Ayala, Lakshmi Jayashankar, Flora Castellino, Obrimpong Amoa-Awua, Manjula Basappa, Britta Flach, Bob C. Lin, Christopher Moore, Mursal Naisan, Muhammed Naqvi, Sandeep Narpala, Sarah O’Connell, Allen Mueller, Leo Serebryannyy, Mike Castro, Jennifer Wang, Gabrielle Dziubla, April K. Randhawa, Michele P. Andrasik, Jenny Hendriks, Carla Truyers, Frank Struyf, Hanneke Schuitemaker, Macaya Douoguih, James G. Kublin, Lawrence Corey, Kathleen M. Neuzil, Linda-Gail Bekker, Nigel Garrett, Sandra W. Cardoso, Patrice DelaFontaine, Craig A. Magaret, Johan Vingerhoets, Martin Casapia, Marcelo H. Losso, Susan J. Little, Aditya Gaur, Edith Swann, Christos J. Petropoulos, Adrian B. McDermott, Jerald Sadoff, Glenda E. Gray, Beatriz Grinsztejn, Paul A. Goepfert, Dean Follmann, Pavitra Roychoudhury, Alexander L. Greninger, Richard A. Koup, Ruben O. Donis, Peter B. Gilbert, the Immune Assays Team, the Coronavirus Vaccine Prevention Network (CoVPN)/ENSEMBLE Team, the United States Government (USG)/CoVPN Biostatistics Team

**Affiliations:** 1 Department of Statistics, University of Washington, Seattle, WA 98195, USA; 2 Vaccine and Infectious Disease Division, Fred Hutchinson Cancer Center, Seattle, WA 98109, USA; 3 Department of Biostatistics, University of Washington, Seattle, WA 98195, USA; 4 Public Health Sciences Division, Fred Hutchinson Cancer Center, Seattle, WA 98109, USA; 5 Department of Mathematics and Statistics, University of North Florida, Jacksonville, FL 32224, USA; 6 Johnson & Johnson Innovative Medicine, Janssen Pharmaceutica NV, 2340 Beerse, Belgium; 7 Johnson & Johnson Innovative Medicine, Janssen Vaccines & Prevention B.V., 2301 Leiden, the Netherlands; 8 Department of Biostatistics and Bioinformatics, Duke University, Durham, NC 27710, USA; 9 Global Health Institute, Duke University, Durham, NC 27710, USA; 10 Biomedical Advanced Research and Development Authority, Administration for Strategic Preparedness and Response, U.S. Department of Health and Human Services, Washington, DC 20201, USA; 11 Vaccine Research Center, National Institute of Allergy and Infectious Diseases, National Institutes of Health, Bethesda, MD 20892, USA; 12 Department of Laboratory Medicine and Pathology, University of Washington, Seattle, WA 98195, USA; 13 Center for Vaccine Development and Global Health, University of Maryland School of Medicine, Baltimore, MD 21201, USA; 14 Institute of Infectious Disease and Molecular Medicine, University of Cape Town, Observatory, Cape Town 7925, South Africa; 15 Department of Medicine, University of Cape Town and Groote Schuur Hospital, Observatory, Cape Town 7925, South Africa; 16 Desmond Tutu HIV Centre, University of Cape Town, Cape Town 7705, South Africa; 17 Centre for the AIDS Programme of Research in South Africa, University of KwaZulu-Natal, Durban, South Africa; 18 Discipline of Public Health Medicine, School of Nursing and Public Health, University of KwaZulu-Natal, Durban 4041, South Africa; 19 Evandro Chagas National Institute of Infectious Diseases-Fundação Oswaldo Cruz, Rio de Janeiro CEP 21.040-900, Brazil; 20 Department of Medicine, Tulane University, New Orleans, LA 70112, USA; 21 Department of Physiology, Tulane University, New Orleans, LA 70112, USA; 22 Department of Pharmacology, Tulane University, New Orleans, LA 70112, USA; 23 Facultad de Medicina Humana, Universidad Nacional de la Amazonia Peru, Iquitos 16004, Peru; 24 Hospital General de Agudos José María Ramos Mejia, Buenos Aires 1221, Argentina; 25 Division of Infectious Diseases, University of California, San Diego, La Jolla, CA 92037, USA; 26 Department of Infectious Diseases, St. Jude Children’s Research Hospital, Memphis, TN 38105, USA; 27 Vaccine Research Program, Division of AIDS, National Institute of Allergy and Infectious Diseases, National Institutes of Health, Bethesda, MD 20892, USA; 28 LabCorp-Monogram Biosciences, South San Francisco, CA 94080, USA; 29 Perinatal HIV Research Unit, Faculty of Health Sciences, University of the Witwatersrand, Johannesburg 2000, South Africa; 30 South African Medical Research Council, Cape Town 7505, South Africa; 31 Division of Infectious Diseases, Department of Medicine, University of Alabama at Birmingham, Birmingham, AL 35294, USA; 32 Biostatistics Research Branch, National Institute of Allergy and Infectious Diseases, National Institutes of Health, Bethesda, MD 20892, USA; 33 Department of Biochemistry, University of Washington, Seattle, WA 98195, USA

**Keywords:** Immune response, Immunology

## Abstract

In Latin American sites of the ENSEMBLE trial of the Ad26.COV2.S vaccine vs. placebo, binding antibodies and neutralizing antibodies measured 4 weeks post-vaccination (∼peak) against circulating lineages (Ancestral, Gamma, Lambda, Mu, Zeta) were assessed as a correlate of risk of, and correlate of protection against, lineage-specific COVID-19. Comparison of lineage-matched controlled vaccine efficacy (VE) curves showed similar relationships across lineages of lineage-specific antibody with VE against lineage-matched COVID-19, supporting a “variant-invariant correlate of protection model” that undergirds immunobridging inferences of efficacy against new variants based on variant-matched neutralizing antibody titers. Lambda departed from this model at undetectable/just-detectable titers: at ∼ peak Reference-specific titers of 2.7 arbitrary units (AU)/mL (just-detectable) and 30 AU/ml, VE against Ancestral COVID-19 was 53.0% (95% CI: 30.7%, 67.9%) and 84.5% (73.6%, 93.0%), respectively; at the same Lambda-specific titers, VE against Lambda COVID-19 was 12.3% (−54.1%, 50.3%) and 91.1% (68.9%, 98.0%). Additional research is needed for Omicron variants.

## Highlights

• Variant-invariant CoP model is evaluated by comparing lineage-matched VE curves
• This model is supported when antibody levels are moderate to high
• Model not supported for Lambda vs. other lineages at lowest antibody levels
• Findings generally support variant-matched neutralizing antibody immunobridging

## Graphical abstract

**Figure:**
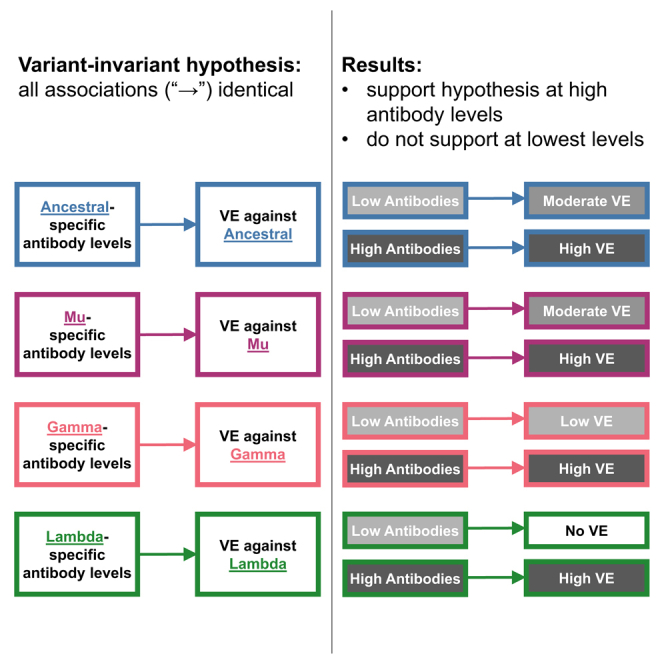

## Introduction

The phase 3 randomized, placebo-controlled ENSEMBLE trial (NCT04505722) of the recombinant replication-incompetent adenovirus vector-based Ad26.COV2.S vaccine was conducted in Latin America (Argentina, Brazil, Chile, Colombia, Mexico, Peru), South Africa, and the United States.^1^ In the final analysis of the double-blind phase, estimated vaccine efficacy (VE) against moderate to severe–critical COVID-19 with onset at least 28 days post-administration was 52.9% [95% confidence interval (CI), 47.1%–58.1%].^2^ Over the follow-up period, new SARS-CoV-2 lineages emerged and circulated in different geographic regions, with e.g., Beta dominant in South Africa, Lambda in Peru, and Mu in Colombia.^1^ With the earliest SARS-CoV-2 strain (GenBank accession number: MN908947) referred to as Index, and this strain with the D614G point mutation referred to as Reference (as in ref.^2^), Sadoff et al. reported that estimated VE against moderate to severe–critical COVID-19 with onset at least 28 days post-administration was higher against COVID-19 caused by Reference viruses [58.2% (35.0%–73.7%)] compared to COVID-19 caused by a lineage other than Reference [44.4% (34.6%–52.8%)].^2^ Magaret et al. reported VE estimates against lineage-specific moderate to severe-critical COVID-19 in Latin America, South Africa, and the United States. Spike diversity was greatest in Latin America where estimated VE (95% CI) against Reference COVID-19 was 64.7% (54.3%, 72.7%); against Zeta COVID-19, 65.0% (48.6%, 76.2%); against Gamma COVID-19, 40.5% (20.7%, 55.3%); against Mu COVID-19, 39.6% (10.1%, 59.5%); and against Lambda COVID-19, 10.8% (−34.6%, 41.0%).^3^ The pattern of decreasing lineage-specific VE tracked with Spike weighted Hamming distances of lineages to the vaccine strain.

The Ad26.COV2.S vaccine expresses the pre-fusion stabilized Spike protein from the SARS-CoV-2 strain equal to the Index strain with changes R682S + R685G + K986P + V987P.^4^ We previously assessed three Day 29 (D29) antibody markers: IgG binding antibodies (bAbs) against Spike (Spike IgG) of the Index strain, IgG bAbs against the Spike receptor-binding domain (RBD IgG) of the Index strain, and 50% inhibitory dilution (ID50) neutralizing antibody titer (nAb-ID50) against pseudovirus expressing Spike from the Reference strain, as correlates of risk (CoR) of moderate to severe-critical COVID-19 and correlates of protection (CoP) against moderate to severe-critical COVID-19.^5^ [ref.^6^^,^^7^^,^^8^ provide additional background on CoRs and CoPs, where a CoR is an immunological marker that correlates with an infectious disease outcome of interest, and a CoP is an immunological marker that statistically associates with (predicts) vaccine efficacy against the outcome.]

Here, we assessed the same Spike IgG and nAb-ID50 markers, except now additionally measured against the SARS-CoV-2 lineages that circulated in the different geographic areas in which the trial was conducted, as CoRs and CoPs of moderate to severe-critical lineage-matched COVID-19. The overarching objective was to determine how well a previously postulated (ref.^5^^,^^9^^,^^10^) “variant-invariant CoP model” holds, which states that the level of antibody to the exposing lineage will have the same statistical relationship with the level of vaccine efficacy against lineage-matched COVID-19 for all lineages.

## Results

### Antibody marker sampling

**Figure 1:**
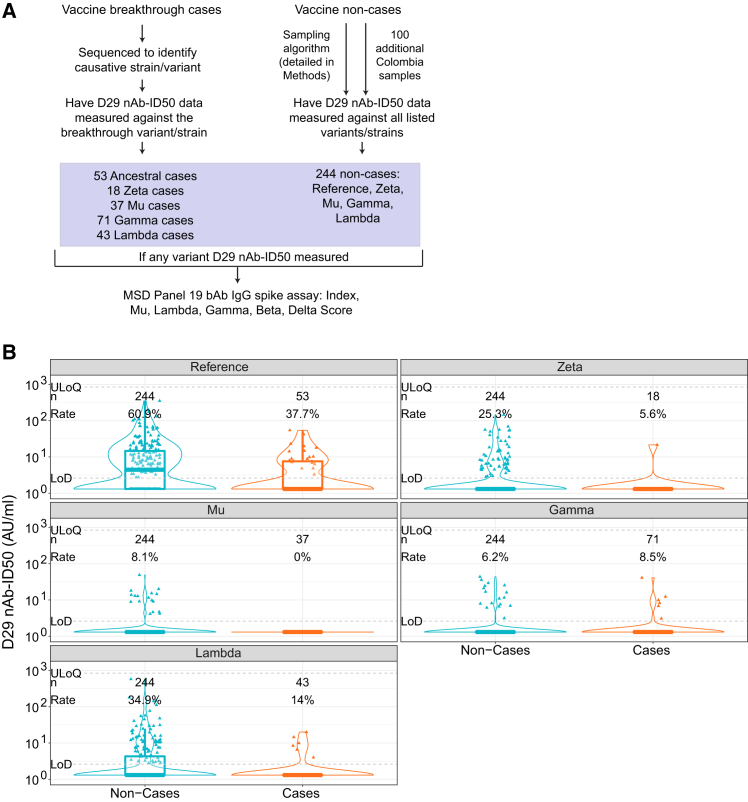
Measurement of variant-specific antibodies and distributions of D29 antibody markers for COVID-19 breakthrough cases vs. non-cases in Latin America (A) Flow diagram showing sampling for the measurement of variant-specific D29 nAb-ID50 titers and variant-specific D29 anti-Spike IgG binding antibody concentration in vaccine breakthrough cases and vaccine non-cases in Latin America. (B) Violin plots of D29 nAb-ID50 in baseline SARS-CoV-2 seronegative per-protocol vaccine recipients in Latin America against each breakthrough case-matched lineage compared to against the same lineage in non-cases. Violin plots contain interior boxplots with upper and lower horizontal edges the 25th and 75th percentiles of antibody level and middle line the 50th percentile, and vertical bars the distance from the 25^th^ (or 75^th^) percentile of antibody level and the minimum (or maximum) antibody level within the 25^th^ (or 75^th^) percentile of antibody level minus (or plus) 1.5 times the interquartile range. Each side shows a rotated probability density (estimated by a kernel density estimator with a default Gaussian kernel) of the data. The limit of detection (LOD) was 2.612 AU/ml (=IU50/mL for Reference) and the minimum upper limit of quantitation (ULOQ) used for truncating readouts was 844.7 AU/ml. n is the number of participants with lineage-specific antibody data, and Rate is the response frequency. For every strain, original nAb-ID50 titers in AU/ml were multiplied by 0.0653, and thus nAb-ID50 Reference titers are equivalently expressed in international units (IU50)/mL. AU, arbitrary units; bAb, binding antibody; IU, international units; nAb-ID50, 50% inhibitory dilution neutralizing antibody titer. See also Figures S9–S12.

With the day of vaccination defined as Day 1 (D1), serum samples were taken on D1 and D29. A case-cohort design^11^ was used to randomly sample participants into an immunogenicity subcohort for antibody marker measurements at D29. The previous studies (ref.^5^ and our unpublished work) measured bAb markers against Index and nAb-ID50 against Reference, for all COVID-19 vaccine breakthrough cases as well as for subcohort members. Based on the same subcohort, the current study measured the Spike IgG markers at D29 against 10 Spike strains including all circulating lineages but Zeta (see the “binding antibody assay” section in STAR Methods) for a stratified random sub-sample of participants and for all COVID-19 vaccine breakthrough cases. Additionally, nAb-ID50 was measured at D29 against (Gamma, Lambda, Mu, Zeta) for a stratified random sub-sample of Latin American participants and against (Delta, Beta) for a stratified random sub-sample of South African participants; moreover, for all COVID-19 vaccine breakthrough cases, if the breakthrough lineage was available, nAb-ID50 titer was measured against the breakthrough lineage. Figure 1A provides a sampling flowchart that shows the numbers of baseline SARS-CoV-2 seronegative per-protocol Latin America vaccine recipient cases and non-cases that were sampled for the measurement of variant-specific D29 nAb-ID50 titers and variant-specific D29 anti-Spike IgG binding antibody concentrations.

### Study cohort, endpoints, and case/non-case definitions

As in our previous work,^5^ the correlates analyses were conducted in per-protocol (defined as in Sadoff et al.^1^) baseline SARS-CoV-2 seronegative participants. Participants with any evidence of SARS-CoV-2 infection up to 6 days post D29 were excluded. The COVID-19 endpoint assessed was moderate to severe-critical COVID-19 (the same primary endpoint of Sadoff et al.^1^^,^^2^) and also requiring the availability of a viral load value. The Statistical Analysis Plan (SAP), publicly available at Figshare,^12^ provides the rationale for this endpoint (Section 13.3). Endpoints with onset both ≥28 days post-vaccination and ≥7 days post-D29 through to the data cut-off date (July 9, 2021) were included. Follow-up was included through 220 days post-vaccination for analyses of Latin America and through 140 days post-vaccination for analyses of South Africa and the United States. Figures S1 and S2 show the SARS-CoV-2 variants causing the COVID-19 endpoints by calendar date of COVID-19 occurrence (Figure S1) and, alternatively, by number of days since D29 (Figure S2), broken down in each plot by geographic region and treatment assignment.

### Correlation of D29 antibody markers

Figures S3 and S4 show pairwise scatterplots of D29 Spike IgG and nAb-ID50 readouts, respectively, against the antigen panels for Latin American participants. Whereas Spike IgG readouts are highly correlated across antigens (r = 0.78 to 0.97), nAb-ID50 titers are only weakly to moderately correlated (r = 0.35 to 0.68). Results are similar for South Africa (Figures S5 and S6). Figures S7 and S8 show correlations between Spike IgG and nAb-ID50 for each common antigen based on Latin America and South Africa data. The readouts are moderately correlated for Index/Reference (r = 0.54 and r = 0.60 for Latin America and South Africa) and weakly or moderately correlated for variants (r = 0.17, 0.23, 0.49 for Gamma, Mu, Lambda in Latin America and r = 0.33, 0.30 for Beta, Delta in South Africa).

### Distributions of D29 antibody markers for COVID-19 breakthrough cases vs. non-cases (Latin America)

Table 1 shows positive response frequencies and geometric mean marker values for vaccine recipient breakthrough cases vs. non-cases for nAb-ID50 and Spike IgG for Latin American participants. nAb-ID50 response frequencies of non-cases are 60.9%, 25.3%, 8.1%, 6.2%, and 34.9% against Reference, Zeta, Mu, Gamma, and Lambda (Figure 1B), with frequencies too low for Mu and Gamma to be able to conduct nAb-ID50 correlates analyses. Response frequencies were much higher for Spike IgG (always exceeding 80%), enabling correlation analyses for Mu and Gamma. For all five lineages evaluated except Gamma, nAb-ID50 levels are lower in lineage-matched COVID-19 breakthrough cases compared to non-cases, with geometric mean ratios (Cases/Non-cases) 0.48 (95% CI 0.32, 0.70), 0.65 (0.44, 0.95), 0.84 (0.74, 0.95), 1.02 (0.85, 1.22), and 0.56 (0.40, 0.78) for Reference, Zeta, Mu, Gamma, and Lambda, respectively. Spike IgG levels follow this pattern for Index and Lambda, but have geometric mean ratios near one for Mu and Gamma. Table S1 shows results for nAb-ID50 and Spike IgG in South Africa and the United States (given the small numbers of variant-specific COVID-19 endpoints in South Africa and the United States, all CoR/CoP statistical inferences are restricted to Latin America, whereas immunogenicity analyses provide results for all three regions). Figure S9 shows results for nAb-ID50 in South Africa, and Figures S10 and S11 show results for Spike IgG in Latin America and South Africa. In both Latin America and South Africa, there is more separation of nAb-ID50 distributions between cases vs. non-cases than for Spike IgG.

**Table 1:** Day 29 nAb-ID50 and spike IgG detectable/positive response frequencies and geometric means by lineage-matched breakthrough cases vs. non-cases in Latin America (Per-Protocol Baseline Seronegative Vaccine Recipients)

D29 Marker	Lineage	Lineage-Matched COVID-19 Cases			Non-Cases			Comparison	
		N	Response frequency	GMT or GMC (AU/ml)	NC	Response frequency	GMT or GMC (AU/ml)	Resp frequency difference (Cases – Non-Cases) (95% CI)	Ratio of GMT or GMC (Cases/Non-Cases) (95% CI)
nAb-ID50	Ref.	53	37.7% (25.5%, 51.7%)	2.88 (2.14, 3.88)	451	60.9% (52.3%, 68.8%)	6.03 (4.70, 7.73)	−0.232 (−0.377, −0.068)	0.48 (0.32, 0.70)
nAb-ID50	Zeta	18	5.6% (0.7%, 34.3%)	1.53 (1.13, 2.05)	244	25.3% (17.8%, 34.7%)	2.35 (1.86, 2.98)	−0.198 (−0.303, 0.099)	0.65 (0.44, 0.95)
nAb-ID50	Mu	37	0.0% (0.0%, 0.0%)	1.31 (1.31, 1.31)	244	8.1% (4.1%, 15.5%)	1.56 (1.38, 1.76)	−0.081 (−0.155, −0.041)	0.84 (0.74, 0.95)
nAb-ID50	Gamma	71	8.5% (3.8%, 17.8%)	1.55 (1.35, 1.78)	244	6.2% (3.0%, 12.2%)	1.51 (1.36, 1.69)	0.023 (−0.053, 0.121)	1.02 (0.85, 1.22)
nAb-ID50	Lambda	43	14.0% (6.3%, 28.3%)	1.72 (1.39, 2.12)	244	34.9% (26.4%, 44.4%)	3.09 (2.39, 4.00)	−0.209 (−0.331, −0.043)	0.56 (0.40, 0.78)
Spike IgG	Index	53	71.7% (57.9%, 82.4%)	23.5 (17.5, 31.5)	451	85.3% (78.2%, 90.3%)	37.6 (31.1, 45.4)	−0.136 (−0.283, −0.008)	0.63 (0.44, 0.88)
Spike IgG	Delta^a^	–	–	–	244	96.0% (89.8%, 98.5%)	23.7 (19.2, 29.2)	–	–
Spike IgG	Beta	–	–	–	413	96.7% (90.3%, 98.9%)	18.2 (15.0, 22.0)	–	–
Spike IgG	Mu	37	83.8% (67.6%, 92.7%)	19.1 (12.3, 29.7)	376	89.4% (82.4%, 93.8%)	17.8 (14.5, 21.9)	−0.056 (−0.224, 0.057)	1.08 (0.66, 1.75)
Spike IgG	Gamma	71	91.6% (82.2%, 96.2%)	16.8 (13.0, 21.7)	342	92.5% (85.4%, 96.3%)	19.4 (16.0, 23.6)	−0.009 (−0.11, 0.075)	0.86 (0.63, 1.19)
Spike IgG	Lambda	43	93.0% (79.9%, 97.8%)	11.3 (7.9, 16.1)	370	95.4% (89.6%, 98.1%)	19.3 (15.5, 23.9)	−0.024 (−0.158, 0.052)	0.58 (0.39, 0.88)

Case = Moderate-to-severe COVID-19 primary endpoint that occurred starting 7 days post D29 through to the end of the blinded phase (220 days for Latin America) with lineage measured/known. N is the number of vaccine recipient breakthrough cases caused by the indicated lineage (measured/known) with D29 marker data against the lineage. Non-case = No acquisition of the moderate-to-severe COVID-19 primary endpoint through to the end of the blinded phase. NC is the number of non-cases with D29 marker data against the lineage. For nAb-ID50, response frequency is the estimated percentage with nAb-ID50 above the lower detection limit (LOD) = 2.612 AU/ml (=IU50/mL for Reference). For spike IgG, response frequency is the estimated percentage with IgG above the minimum lower limit of quantitation (LLOQ) = 1.683 AU/ml (=BAU/ml for Index). GMT is the estimated geometric mean titer of nAb-ID50, and GMC is the estimated geometric mean concentration of Spike IgG. For every strain, original nAb-ID50 titers in AU/ml were multiplied by 0.0653 and thus nAb-ID50 Reference titers (but not any of the variant nAb titers) are equivalently expressed in international units (IU50)/mL; for every strain, all original Spike IgG readouts in AU/ml were multiplied by 0.009 and thus Spike IgG Index concentrations (but not any of the variant Spike IgG concentrations) are equivalently expressed in binding antibody units (BAU)/mL.

See also Figures S1 and S2; Table S1.

a Response frequencies and GMCs are for Delta-score (maximal signal diversity weighted average readouts to the five Delta strains in MSD Panel 19).

Figure S12 presents the results of a post hoc analysis equivalent to that done for Figure 1B (D29 nAb-ID50 Reference, Zeta, Mu, Gamma, and Lambda titers and response frequencies in Latin American participants), except separately in males and females.

### Classification of immune correlates analyses

Table S2 classifies the six different statistical analyses conducted for assessing D29 antibody markers as CoRs and CoPs specific to the SARS-CoV-2 lineage or Spike amino acid sequence distance to the vaccine-strain at a specific D29 marker value or for subgroups defined by a range of D29 marker values.

### Proportional hazards model correlates of risk results (Latin America)

**Figure 2:**
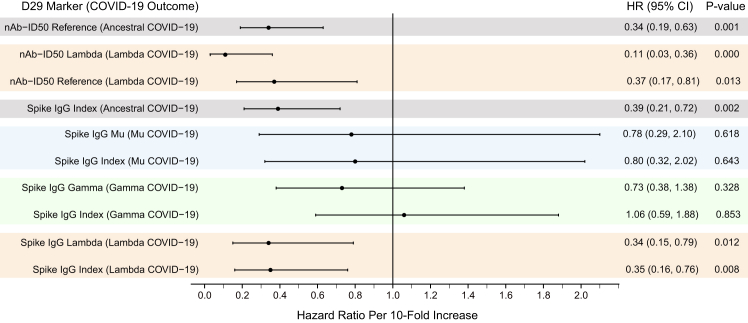
Proportional hazards model correlates of risk results for quantitative antibody markers Analyses were performed in baseline SARS-CoV-2 seronegative per-protocol vaccine recipients in Latin America. The forest plot shows hazard ratio point estimates and 95% confidence intervals of lineage-specific COVID-19 per 10-fold increase in D29 lineage-specific nAb-ID50 or nAb-ID50 Reference. Hazard ratios were estimated using a Cox model with adjustment for baseline risk score. For every strain, original nAb-ID50 titers in AU/ml were multiplied by 0.0653, and thus nAb-ID50 Reference titers are equivalently expressed in international units (IU50)/mL; for every strain, all original Spike IgG readouts in AU/ml were multiplied by 0.009, and thus Spike IgG Index concentrations are equivalently expressed in binding antibody units (BAU)/mL. AU, arbitrary unit; nAb-ID50, 50% inhibitory dilution neutralizing antibody titer. See also Tables S3 and S4.

For each lineage with at least 25 breakthrough cases in a geographic region (only Latin America qualified), a Cox model was used to estimate the hazard ratio (HR) of lineage-specific COVID-19 by level of D29 antibody marker against the specific lineage. To assess if lineage-matching improves the correlate of risk, we also estimated the HRs for each D29 antibody marker measured to the Index or Reference strain. Figure 2 shows results for nAb-ID50 and Spike IgG. Of the two lineages evaluable for nAb-ID50 analysis (Reference, Lambda), lineage-matched titer was a strong inverse CoR for both lineages, with point estimates suggesting a potentially stronger CoR for Lambda [Reference HR = 0.37 (0.17, 0.81) per 10-fold titer increase; Lambda HR 0.11 (0.03, 0.36)]. Of the four lineages evaluable for Spike IgG analysis (Reference, Lambda, Gamma, Mu), the inverse CoR appeared stronger for Lambda [HR 0.34 (0.15, 0.79)] than for Gamma [HR 0.73 (0.38, 1.38) and Mu (HR 0.78 (0.29, 2.10)]. In addition, the nAb-ID50 lineage-matched HR was lower than the Reference antibody HR for Lambda COVID-19, supporting that lineage-matching strengthens the correlate. In contrast, the Spike IgG lineage-matched and Index HRs were similar for both Lambda and Mu COVID-19. Table S3 presents the results of a post hoc analysis estimating the same HRs as in Figure 2, except done separately in males and females.

**Figure 3:**
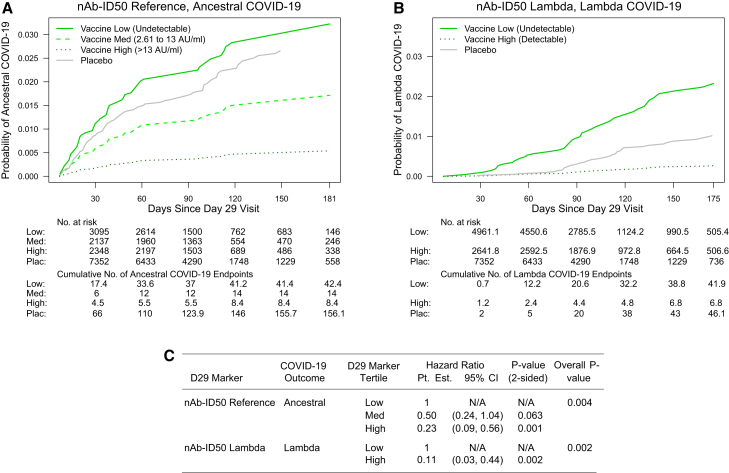
Proportional hazards model correlates of risk results for categorical antibody markers Analyses were performed in baseline SARS-CoV-2 seronegative per-protocol vaccine recipients in Latin America. (A and B) Covariate-adjusted cumulative incidence of lineage-matched COVID-19 by Low, Medium, or High tertile of D29 nAb-ID50 Reference titer or by Undetectable vs. Detectable D29 nAb-ID50 Lambda titer: A) nAb-ID50 Reference, Ancestral COVID-19; B) nAb-ID50 Lambda, Lambda COVID-19. (C) Estimated hazard ratios and 95% confidence intervals of lineage-matched COVID-19 for the Medium versus Low, High versus Low, Detectable vs. Undetectable subgroups. *p*-value (2-sided) is from a Wald test of whether the hazard rate of lineage-matched COVID-19 differed between the subgroup Med or High compared to the Low subgroup. The overall *p* value is from a generalized Wald test of whether the hazard rate of lineage-matched COVID-19 differed across the 3 or 2 subgroups. Analyses adjusted for baseline risk score. For every strain, original nAb-ID50 titers in AU/ml were multiplied by 0.0653, and thus in (A), nAb-ID50 Reference cut-points separating Low vs. Medium vs. High in AU/ml are equivalently expressed in international units (IU50)/mL. AU, arbitrary unit; CI, confidence interval; nAb-ID50, 50% inhibitory dilution neutralizing antibody titer; Pt. Est., point estimate. Non-integer numbers of COVID-19 endpoints are due to the application of the inverse probability of sampling D29 marker weights. See also Figure S13.

To evaluate whether one or both assays, nAb-ID50 and Spike IgG, are independent inverse CoRs, we also fit the Cox models including both assays for each of the two lineages of COVID-19 that could be studied (Ancestral, Lambda) (Table S4). For both lineages, the lineage-matched HR was lower for nAb-ID50 than Spike IgG, supporting that neutralizing antibodies are the independent correlate of risk [e.g., HR of Lambda COVID-19 = 0.13 (0.03, 0.50) per 10-fold increase in nAb-ID50 Lambda vs. 0.74 (0.29, 1.87) per 10-fold increase in Spike IgG Lambda]. Figure 3 and S13 show covariate-adjusted cumulative incidence of lineage-matched COVID-19 for subgroups with Low, Medium, and High tertile D29 lineage-specific nAb-ID50 titer and Spike IgG, respectively, where for Lambda nAb-ID50, more than a third had undetectable responses, such that undetectable vs. detectable subgroups were studied. Cumulative incidence significantly decreased with increasing response subgroup for both assays, with High-tertile (Reference) and nAb-ID50 detectable (Lambda) vaccine recipients having markedly the lowest incidence.

### Nonparametric threshold correlates of risk results (Latin America)

For the same D29 antibody markers lineage-matched to COVID-19 outcomes assessed in Cox models, we applied nonparametric threshold modeling with adjustment for behavioral risk score (Figure S14). For all six CoR analyses, the probability of COVID-19 decreased across subgroups of vaccine recipients with increasing threshold of response. For example, the probability of acquiring Lambda COVID-19 over 7 months was 0.0098 (0.0053, 0.0141) for all vaccine recipients and 0.0042 (0.0007, 0.0079) for vaccine recipients with nAb-ID50 Lambda exceeding 10 arbitrary units (AU)/mL.

### Controlled vaccine efficacy correlates with protection results (Latin America)

**Figure 4:**
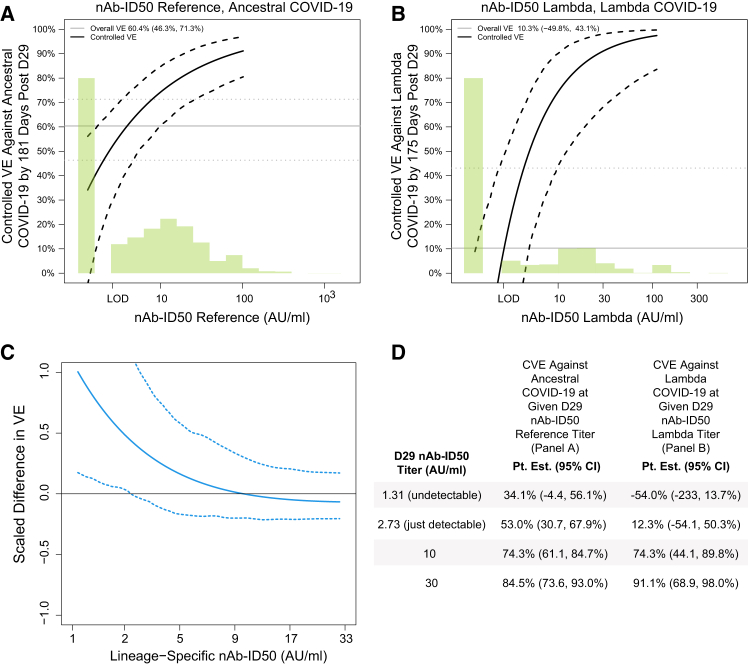
Controlled vaccine efficacy (CVE) curves and the contrast in curves for D29 lineage-matched nAb-ID50 Analyses were performed in baseline SARS-CoV-2 seronegative per-protocol participants in Latin America. (A) CVE for nAb-ID50 Reference, Ancestral COVID-19. (B) CVE for nAb-ID50 Lambda, Lambda COVID-19. (C) Scaled difference between the two CVE curves calculated as: log_10_{CVEcontrast(s)} = log_10_{1- CVE-(Lambda COVID-19)(s)} – log_10_{1 – CVE-(Ancestral COVID-19)(s)}. Solid lines are point estimates, and dashed lines are 95% pointwise confidence intervals. Curves are plotted ranging from the LOD/2 to the 97.5^th^ percentile of nAb-ID50 (LOD = 2.612 AU/ml). (D) For (A) and (B), the table shows point estimates and 95% confidence intervals of CVE against either Ancestral COVID-19 or Lambda COVID-19 at four different D29 nAb-ID50 titer values. For every strain, original nAb-ID50 titers in AU/ml were multiplied by 0.0653, and thus nAb-ID50 Reference titers in (A) are equivalently expressed in international units (IU50)/mL. AU, arbitrary unit; LOD, limit of detection; nAb-ID50, 50% inhibitory dilution neutralizing antibody titer. See also Figures S15 and S16.

Figure 4 shows the lineage-matched controlled VE curves for nAb-ID50 against Ancestral COVID-19 and against Lambda COVID-19, as well as the log_10_-transformed CVEcontrast (j, j’; s) curve. The variant-invariant CoP model across a range of s values for a pair of lineages j vs. j’ would be supported by the log_10_ transformed CVEcontrast (j, j’; s) curve equaling zero at those s values. The results support the fidelity of the variant-invariant CoP model at nAb-ID50 of about 5 AU/ml and above, and some model deviation at the lowest titers. At just-detectable nAb-ID50 titer [2.73 AU/ml, slightly higher than the limit of detection (LOD) of 2.612 AU/ml], VE against Ancestral and against Lambda is 53.0% (30.7, 67.9%) and 12.3% (−54.1, 50.3%), respectively, whereas at a nAb-ID50 titer of 10 AU/ml, VE against Ancestral and against Lambda increases to 74.3% (61.1, 84.7%) and 74.3% (44.1, 89.8%), respectively. Figure S15 shows the Spike IgG lineage-matched controlled VE curves and Figure S16 the corresponding log_10_-transformed Spike IgG CVEcontrast curves. The results support the variant-invariant CoP model for the lineage pairs (Ancestral, Gamma), (Ancestral, Mu), (Gamma, Lambda), (Gamma, Mu), and, consistent with the results for nAb-ID50, departures from the model at lowest antibody levels with VE approximately zero against Lambda COVID-19 at unquantifiable Spike IgG compared to VE approximately 25–40% against Ancestral and Mu COVID-19 at unquantifiable Spike IgG.

### Nonparametric threshold correlates of vaccine efficacy results (Latin America)

**Figure 5:**
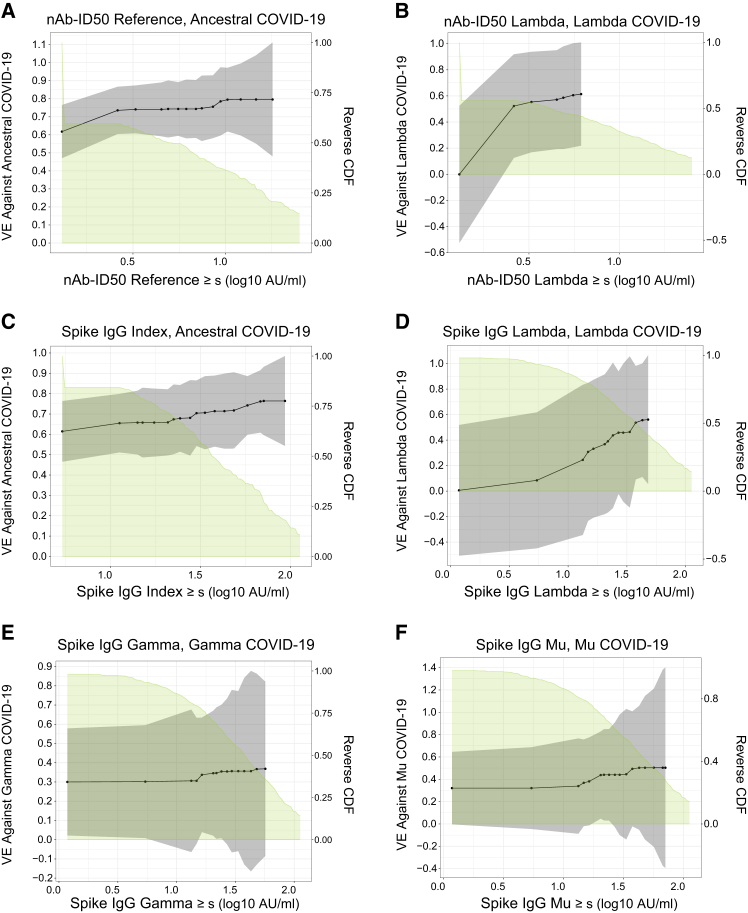
Vaccine efficacy against lineage-specific COVID-19 by D29 lineage-matched nAb-ID50 titer above a threshold or by D29 lineage-matched Spike IgG concentration above a threshold for Latin America circulating lineages Vaccine efficacy is one minus the lineage-specific covariate-adjusted thresholded cumulative incidence in vaccine recipients divided by the lineage-specific covariate-adjusted cumulative incidence in placebo recipients. Dots represent point estimates at given threshold values. (A) VE against Ancestral COVID-19, nAb-ID50 Reference titer. (B) VE against Lambda COVID-19, nAb-ID50 Lambda titer. (C) VE against Ancestral COVID-19, Spike IgG Index concentration. (D) VE against Lambda COVID-19, Spike IgG Lambda concentration. (E) VE against Gamma COVID-19, Spike IgG Gamma concentration. (F) VE against Mu COVID-19, Spike IgG Mu concentration. For every strain, original nAb-ID50 titers in AU/ml were multiplied by 0.0653, and thus nAb-ID50 Reference titers are equivalently expressed in international units (IU50)/mL; for every strain, all original Spike IgG readouts in AU/ml were multiplied by 0.009, and thus Spike IgG Index concentrations are equivalently expressed in binding antibody units (BAU)/mL. To define the set of dots/thresholds for reporting of point estimates for the nAb-ID50 assay [(A) and (B)], first, a common grid of thresholds was obtained by quantile binning the D29 nAb-ID50 Reference titer values at COVID-19 endpoints (including all endpoints regardless of lineage) into 20 equal-frequency bins. Then, for each lineage of COVID-19, black dots at which fewer than 5 lineage-specific COVID-19 endpoints had lineage-matched nAb-ID50 titers above the dot value were excluded. For (C)-(F), the same process was applied based on D29 Spike IgG Index values and on lineage-matched D29 Spike IgG values. The solid black lines are obtained by linearly interpolating the grid points. The gray shaded area indicates pointwise 95% CIs. The estimates and CIs were adjusted using the assumption that the true threshold-response vaccine efficacy is non-increasing. The upper boundary of the green shaded area is the estimate of the reverse cumulative distribution function (CDF) of the D29 antibody marker level in vaccine recipients. Analyses adjusted for baseline behavioral risk score. AU, arbitrary unit; nAb-ID50, 50% inhibitory dilution neutralizing antibody titer. See also Figure S14.

Figure 5 shows the results corresponding to the threshold correlates of risk results in Figure S14. For all six analyses, thresholded vaccine efficacy increases with increasing threshold of response, most strongly for nAb-ID50 Lambda and Spike IgG Lambda with Lambda COVID-19 (VE increasing from about 0% to 60%). In contrast, thresholded vaccine efficacy for nAb-ID50 Reference and Spike IgG Index with Ancestral COVID-19 increases from about 60% to 80%.

### Sequence distance-specific correlates of risk and protection (Latin America)

**Figure 6:**
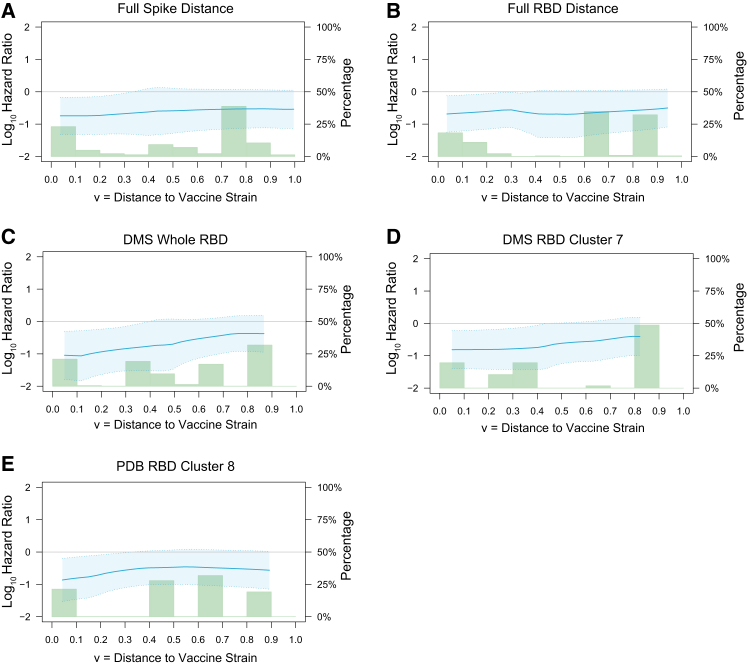
Hazard ratio analysis of the dependency of SARS-CoV-2 amino acid sequence distance-specific COVID-19 risk on D29 nAb-ID50 Reference titer Analyses were performed in baseline seronegative per-protocol vaccine recipients in Latin America. Each panel shows the log_10_ hazard ratio of distance-specific COVID-19 per 10-fold increase in D29 nAb-ID50 (left y axis) for the given distance to the vaccine strain. (A) Weighted Spike Hamming distance. (B) Weighted RBD Hamming distance. (C) DMS whole-RBD escape score. (D) DMS cluster 7-RBD escape score. (E) PDB cluster 8-RBD escape score. Distances are from COVID-19 endpoint sequences to the vaccine-strain sequence and are defined in Magaret et al.^3^ Shaded regions are 95% pointwise confidence intervals. Histograms of the distances are in green (right y axis). See also Table S5 and Figure S17.

**Figure 7:**
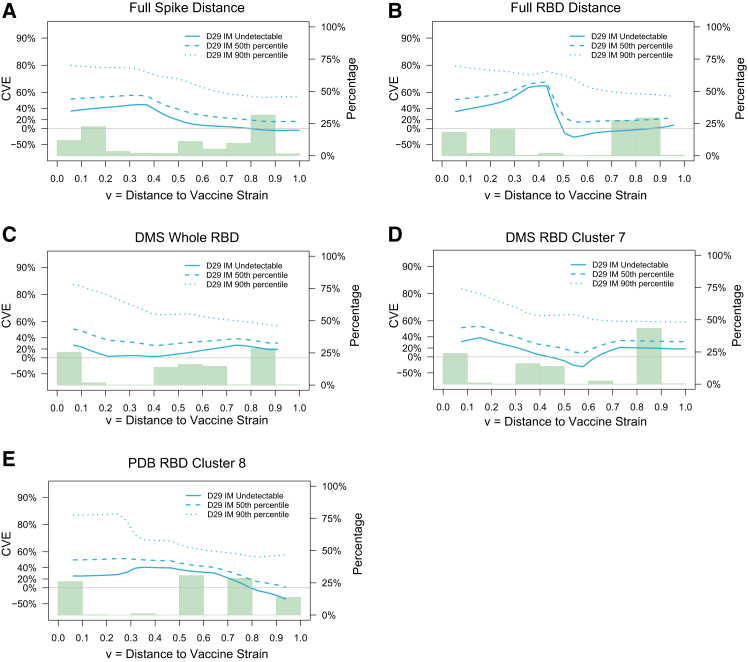
SARS-CoV-2 amino acid sequence distance-specific vaccine efficacy against COVID-19 (including all strains) at three levels of D29 nAb-ID50 Reference titer Analyses were performed in baseline seronegative per-protocol participants in Latin America. Each panel shows the distance-specific cumulative vaccine efficacy at nAb-ID50 values Low, Medium, and High (Low: undetectable = 1.31 AU/ml; Medium: 50^th^ percentile = 6.27 AU/ml; High: 90^th^ percentile = 37.8 AU/ml). (A) Weighted Spike Hamming distance. (B) Weighted RBD Hamming distance. (C) DMS whole-RBD escape score. (D) DMS cluster 7-RBD escape score. (E) PD8 cluster 8-RBD escape score. Distances are from COVID-19 endpoint sequences to the vaccine-strain sequence and are defined in Magaret et al.^3^ Shaded regions are 95% pointwise confidence intervals. Histograms of the distances are in green (right y axis).

Hypothesis testing generally did not show evidence that the D29 nAb-ID50 Reference correlates of risk varied with the distance of the COVID-19 endpoint to the vaccine strain (smallest *p*-value 0.074, Table S5). However, point estimates of distance-specific HRs per 10-fold increase in nAb-ID50 Reference showed consistent trends of weaker inverse CoRs against COVID-19 with farther distances from the vaccine strain (Figure 6). Point estimates of the ratios of the distance-specific cumulative incidence rate function^13^ for 50^th^ percentile vs. undetectable and 90^th^ percentile vs. undetectable D29 marker values showed decreasing ratios with viral distances (Figure S17). In addition, point estimates of distance-specific VE at low, medium, and high D29 nAb-ID50, again defined by undetectable, 50^th^ percentile, 90^th^ percentile, generally decreased with distance (Figure 7). The distance-specific VE curves are lowest and approximately flat for undetectable, highest with a steep decline with distance for the 90^th^ percentile, with intermediate results for the 50^th^ percentile.

### Exposure-proximal correlates of vaccine efficacy results (Latin America)

**Figure 8:**
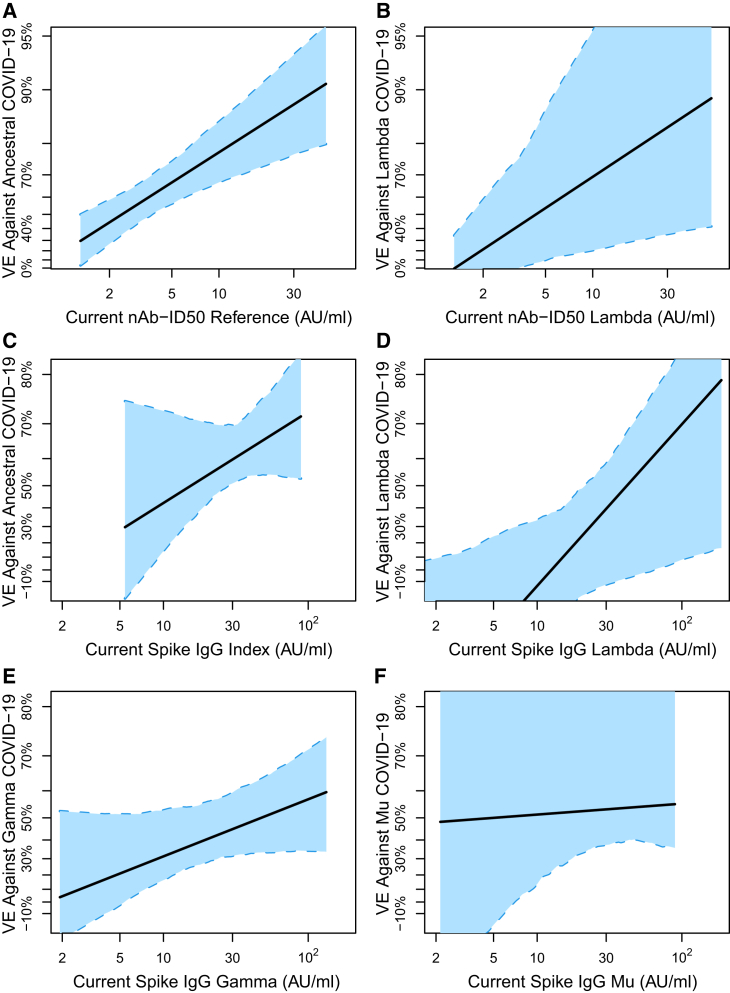
Exposure-proximal vaccine efficacy against lineage-specific COVID-19 by current lineage-matched antibody marker level Analyses were performed in baseline seronegative per-protocol participants in Latin America. Exposure-proximal vaccine efficacy estimates against the given COVID-19 lineage through 220 days post-vaccination by current lineage-specific antibody marker level were obtained using the method of Huang and Follmann,^18^ with “current” referring to the true underlying antibody marker level not subject to technical measurement error, in a hypothetical scenario where the immunoassay was conducted on serum samples drawn on every day of follow-up (see STAR Methods). (A) VE against Ancestral COVID−19, Current nAb−ID50 Reference. (B) VE against Lambda COVID−19, Current nAb−ID50 Lambda. (C) VE against Ancestral COVID−19, Current Spike IgG Index. (D) VE against Lambda COVID−19, Current Spike IgG Lambda. (E) VE against Gamma COVID−19, Current Spike IgG Gamma. (F) VE Against Mu COVID−19, Current Spike IgG Mu. For every strain, original nAb-ID50 titers in AU/ml were multiplied by 0.0653 and thus nAb-ID50 Reference titers are equivalently expressed in international units (IU50)/mL; for every strain, all original Spike IgG readouts in AU/ml were multiplied by 0.009 and thus Spike IgG Index concentrations are equivalently expressed in binding antibody units (BAU)/mL. Each point on the curve represents the vaccine efficacy at the given current antibody marker level: A, B) 50% inhibitory dilution neutralizing antibody (nAb-ID50) titer, and C-F) anti-Spike IgG binding antibody concentration. The dashed lines are bootstrap pointwise 95% CIs. Analyses adjusted for baseline behavioral risk score. AU, arbitrary units/ml; nAb-ID50, 50% inhibitory dilution neutralizing antibody titer.

While antibody levels generally remained steady, with little waning over the follow-up period for capturing COVID-19 endpoints for correlates (Figure S18 and also supported by data in ref.^14^^,^^15^^,^^16^), we nevertheless sought to incorporate this mild waning into our analyses. One alternative approach to assessing the variant-invariant CoP model based on the antibody marker levels at D29 considers the antibody markers over time and assesses exposure-proximal antibody levels as lineage-matched correlates of instantaneous VE. Precedent for this analysis (without the lineage issue) is Follmann et al.^17^ The exposure-proximal results (Figure 8) are similar to those based on the D29 antibody marker models.

## Discussion

The ENSEMBLE trial at Latin American sites provided a unique opportunity to attain insights into correlates of protection against SARS-CoV-2 variants, given the rigor of a randomized, placebo-controlled trial, the large number of enrolled participants, and COVID-19 breakthrough endpoints evaluable for correlates analyses, and the wide diversity of circulating strains. Correlate of risk (CoR) analyses of nAb-ID50 and Spike IgG measured at D29 found that for both these assays, the Reference/Index and Lambda markers were each strongly inversely associated with lineage-matched COVID-19, with point estimates suggesting a stronger CoR for Lambda COVID-19. In contrast, the Gamma-matched and Mu-matched IgG Spike inverse CoRs were weaker and not statistically significant, perhaps due to especially low antibody levels against these variants. Lineage-matching strengthened the nAb-ID50 correlate of Lambda COVID-19 compared to measuring neutralizing antibodies against the Reference strain, whereas, in contrast, for the Spike IgG assay, the correlate was similar when lineage-matching vs. using the vaccine-strain marker. Multivariable models including both nAb-ID50 and Spike IgG supported that nAb-ID50 is the independent CoR, where nAb-ID50 and Spike IgG were less correlated with one another when measured against variants than when measured against Reference/Index. Taken together, these results support that neutralizing antibodies are a stronger CoR of COVID-19 than binding antibodies against Spike and suggest that Lambda has distinct properties compared to the other variants (discussed later in discussion).

In the correlates of protection (CoP) evaluation of the variant-invariant CoP model, there was generally good adherence to the model for the evaluated pairs of lineages [(Reference, Lambda) for nAb-ID50 and (Reference, Mu, Gamma, Lambda) for Spike IgG] for antibody levels well above undetectable/unquantifiable. However, at the lowest antibody levels, the data supported model departures, with substantial vaccine efficacy (25–40%) against Ancestral COVID-19 at undetectable nAb-ID50 and at unquantifiable Spike IgG, as well as against Mu COVID-19 at unquantifiable Spike IgG. Relatedly, both nAb-ID50 and Spike IgG were superior CoPs for Lambda COVID-19 than for Ancestral COVID-19, with superiority defined by a greater change in controlled vaccine efficacy across the span of antibody levels and by a greater proportion of vaccine efficacy mediated through the antibody level, which can be inferred from the controlled vaccine efficacy curves.^6^

These CoP results pose the question as to what is special about Lambda, where undetectable/unquantifiable antibody levels against Lambda successfully marked the absence of protective immunity, a quality of a perfect CoP, whereas these negative responses to Index/Reference (and to Mu for Spike IgG) did not? A potential explanation is that other immune responses, such as Fc-mediated antibody or T cell responses, protected vaccine recipients with undetectable/unquantifiable neutralizing antibody levels for Ancestral and Mu COVID-19 but not for Lambda COVID-19. Another potential explanation is lineage differences in the technical definition of the LOD/lower limit of quantitation (LLOQ) assay limits, where only for Lambda was the limit low enough/sensitive enough to detect the lowest-level protective antibody responses. A third potential explanation is the Lambda variant deletion RSYLTPGD246-253N in the NTD supersite epitope (an offered explanation for why efficacy was especially low against Lambda^3^), or the RBD point mutations F490S and L451Q (which disrupt a hydrophobic domain that is critical for neutralizing antibodies and immune escape),^19^ could imply that an undetectable/unquantifiable response to Lambda constitutes a different immunological phenotype compared to this negative response against the other lineages. Another possibility is the limitation of data/precision, where sampling variability could cause a false positive inference.

The sequence-distance specific CoR and CoP analyses supported that the nAb-ID50 immune correlates were stronger against viruses more proximal to the vaccine strain, although the limited evolution of SARS-CoV-2 during the ENSEMBLE trial follow-up limited the magnitude of this effect and rendered the analysis to have limited statistical power. Had Omicron emerged during ENSEMBLE, we reason the results would have been stronger and statistically significant, given the largely increased range of viral distances to the vaccine strain. Indeed, clinical studies have shown that neutralizing antibody titers induced by Ancestral-based vaccine strains are much more reduced against Omicron strains than against any of the variants that circulated in ENSEMBLE (e.g., COVAIL study,^20^ Tables S6–S11). The fact that ENSEMBLE Ad26.COV2.S vaccine efficacy strongly decreased with increasing spike protein neutralizing antibody escape score distances^3^ implying a neutralizing antibody immune correlate is expected to weaken against SARS-CoV-2 strains with increasing antigenic distance to the vaccine strain.

The current study is unique in our knowledge as the only randomized, placebo-controlled VE trial with enough COVID-19 incidence with multiple circulating lineages to directly test the variant-invariant CoP model via individual-level CoP analysis. One conclusion of the study results is the re-enforcement of the current practice to use variant-matched neutralizing antibody titers for guiding booster insert strain selection in immunobridging studies. [In immunobridging studies, an immunological biomarker that has been supported to provide a basis for predicting vaccine efficacy for a certain set of conditions (e.g., age group, dose level, formulation), is used as the basis for inference of vaccine efficacy in a different set of conditions (e.g., a different age group, dose level, or vaccine formulation).^21^^,^^22^ Immunobridging studies have been performed for COVID-19 vaccines (e.g., Ref.^23^^,^^24^^,^^25^^,^^26^^,^^27^^,^^28^^,^^29^) and have been used to guide regulatory decisions on their use (e.g., ref.^22^^,^^30^).] This conclusion has also been supported by population-level CoP analyses of a series of randomized and test negative design studies (e.g., Khoury et al.^31^).

### Limitations of the study

We conclude with a discussion of the limitations of our study. One limitation is that the variant-invariant CoP model could only be evaluated for lineages prior to Omicron. While extending our results to Omicron variants is of considerable interest, it appears not possible to test the same variant-invariant CoP model hypothesis for Omicron, given widespread vaccination that has altered the addressable question from assessing CoPs for vaccine vs. placebo/unvaccinated to assessing CoPs for booster vs. no booster, as studied by Khoury et al.^31^ Future research of interest would assess booster vs. no booster CoPs for Omicron variants. It is also unknown whether results would differ for other vaccine platforms. Another limitation is that the analyses were conducted in baseline SARS-CoV-2 seronegative participants, and it is unknown whether results would differ in the current high global seropositivity context, where the majority of people have been vaccinated and/or infected repeatedly. Additionally, the two sex-based analyses were performed post hoc, and as such are exploratory and hypothesis-generating in nature. The two post hoc analyses are insufficient to support a conclusion on whether the results of this study apply equally well to males and females.

## Resource availability

### Lead contact

Requests for further information and resources should be directed to and will be fulfilled by the lead contact, Peter B. Gilbert (pgilbert@fredhutch.org).

### Materials availability

There are restrictions on the availability of the pCXAS-SARS-CoV-2-Spike [MN908947.3, Reference (D614G), Beta, Delta, Gamma, Lambda, and Mu] expression vectors because their sequences are proprietary to Monogram.

### Data and code availability

• The data sharing policy of Janssen Pharmaceutical Companies of Johnson & Johnson is available at https://www.janssen.com/clinical-trials/transparency. The data supporting the findings of this study may be obtained from the authors upon reasonable request. Links to the accession codes of the source for the codon-optimized version of the full-length spike gene and the reference sequence for SARS-CoV-2 Spike gene sequence alignments are in the key resources table.
• Code used for SARS-CoV-2 genome assembly,^32^ SARS-CoV-2 lineage assignment,^33^^,^^34^ and distance-specific correlates of risk and protection analyses^13^ has already been described. Additional immune correlates analyses were done reproducibly based on R scripts that have been deposited at GitHub and are publicly available at https://doi.org/10.5281/zenodo.13968569^35^ (reproducible reporting workflow for statistical analyses of candidate immune correlates of risk and protection in vaccine efficacy trials, including exposure-proximal immune correlates analyses; includes multiple modular workflows for correlates of risk/protection analyses and automated reporting of analytic results) and https://doi.org/10.5281/zenodo.14117624^36^ (code to conduct the marker-thresholded correlate of protection analyses) as well as based on R code that is hosted on The Comprehensive R Archive Network (CRAN) and is publicly available at https://doi.org/10.32614/CRAN.package.vaccine^37^ (code to conduct the correlates of protection analyses based on the controlled vaccine efficacy framework).
• Other items: Any additional information required to reanalyze the data reported in this article is available from the lead contact upon request.

## Acknowledgments

This project has been funded in whole or in part with Federal funds from the Department of Health and Human Services (HHS); the 10.13039/100021704Administration for Strategic Preparedness and Response (ASPR); the 10.13039/100012399Biomedical Advanced Research and Development Authority (BARDA), under Other Transaction Number: 75A50123D00005, for RRPV Project Title: RRPV-24-04-NGVxStats-007; NextGen Vaccines: Statistical Support, Correlates of Protection, and Meta-analysis Module 2, as well as under contract numbers HHSO100201700018C with Janssen, 75A50122C00008 with Labcorp—Monogram Biosciences, and 75A50122C00013 with Nexelis Laboratories. This work was also supported by the 10.13039/100000002National Institutes of Health, 10.13039/100000060National Institute of Allergy and Infectious Diseases (NIAID) under the Public Health Service Grants UM1 AI068635 (HVTN SDMC) (PBG), UM1 AI068614 (HVTN LOC) (LC) and R37AI054165 (PBG); the Intramural Research Program of NIAID; and the Scientific Computing Infrastructure at Fred Hutch, under 10.13039/100016958ORIP grant S10OD028685. This work was also supported by Janssen Research and Development, an affiliate of Janssen Vaccines and Prevention, and part of the Janssen pharmaceutical companies of 10.13039/100004331Johnson & Johnson.

The content is solely the responsibility of the authors and does not necessarily represent the official views of the 10.13039/100000002National Institutes of Health. The content also does not necessarily represent the official views of the Department of Health and Human Services, the U.S. government, or any of its components. The contracts and federal funding are not an endorsement of the study results, product or company.

This article is the result of funding in whole or in part by the 10.13039/100000002National Institutes of Health (NIH). It is subject to the NIH Public Access Policy. Through acceptance of this federal funding, NIH has been given the right to make this article publicly available in PubMed Central upon the Official Date of Publication, as defined by NIH.

## Author contributions

Conceptualization: AL, YF, YH, AK, MCarone, JS, and PBG. Curated data: AL, YF, LvdL, FH, YH, YL, CY, SR, MLG, GAVR, DJS, IVD, AK, MCarone, OH, AKR, CAM, PR, ALG, and PBG. Formal analysis: AL, YF, LvdL, FH, YH, YL, CY, SR, MLG, GAVR, DJS, IVD, AK, and PBG. Funding acquisition: PBG. Investigation: OA-A, MB, BF, BCL, CM, MNaisan, MNaqvi, SN, SO’C, AM, SL, MC, JW, GD, JH, CT, FS, HS, MD, JGK, LC, KMN, LGB, NG, SWC, PDF, JV, MCasapia, MHL, SJL, AG, ES, CJP, ABM, JS, GEG, BG, PAG, PR, ALG, and PBG. Methodology: AL, YF, LvdL, FH, YH, AK, MCarone, and PBG. Project administration: VA, KM, LJ, FC, SO’C, AKR, MPA, PR, ALG, RAK, ROD, and PBG. Resources: SR, MLG, GAVR, DJS, MLG, IVD, VA, KM, LJ, FC, AKR, MPA, JH, CT, FS, HS, MD, JGK, LC, KMN, LGB, NG, SWC, PDF, CAM, JV, MCasapia, MHL, SJL, AG, ES, CJP, ABM, JS, GEG, BG, PAG, DF, PR, ALG, RAK, and ROD. Software: AL, YF, LvdL, FH, YH, and AK. Supervision: JGK, LC, KMN, CJP, ABM, MLG, JS, GEG, BG, PAG, DF, PR, ALG, RAK, ROD, and PBG. Visualization: AL, YF, LvdL, FH, YH, YL, CY, LNC, AK, MC, OH, and PBG. Writing – original draft: LNC and PBG. Writing – review and editing: all authors.

Role of the funding source: Biomedical Advanced Research and Development Authority (BARDA) and National Institute of Allergy and Infectious Diseases co-authors co-led assay development and sample testing programs, contributed to the study design and analyses, and revised and approved the final version of the article.

## Declaration of interests

SR, IVD, CT, and JV are employees of Janssen Pharmaceutica NV and have stock and/or stock options in Johnson & Johnson. JH is an employee of Janssen Vaccines & Prevention B.V. and has stock and/or stock options in Johnson & Johnson. At the time of the study, MLG, GAVR, DJS, HS, MD, and JS were employees of Janssen Vaccines & Prevention B.V. and held stocks/options in Johnson & Johnson. JS also has patents on the invention of the Janssen COVID-19 vaccine. FS was an employee of Janssen Pharmaceutica NV and owns shares in Johnson & Johnson. FS also has shares in GlaxoSmithKline as compensation for past employment. PAG has a patent with Aridis Pharmaceuticals for a COVID-19 monoclonal antibody.

## STAR★Methods

### Key resources table

**Table:** 

REAGENT or RESOURCE	SOURCE	IDENTIFIER
**Biological samples**		

Human serum samples from participants in the ENSEMBLE trial	Sadoff et al.^2^	N/A
Nasal swab samples from SARS-CoV-2 RT-PCR confirmed cases in the ENSEMBLE trial	Sadoff et al.^2^	N/A

**Chemicals, peptides, and recombinant proteins**		

Steady Glo reagent	Promega	Promega manufactures custom lots for Monogram

**Critical commercial assays**		

V-PLEX SARS-CoV-2 Panel 19 (IgG) Kit (MSD)	Meso Scale Discovery	K15540U
Swift Normalase Amplicon Panel (SNAP)	Swift Biosciences	Then: Catalog # CovG1 V2-96, Now: xGen SARS-CoV-2 Amplicon Panel

**Experimental models: Cell lines**		

HEK-293	Master Cell Bank established by Monogram Biosciences	LC002749

**Recombinant DNA**		

F-lucP.CNDOΔU3 (backbone vector used to generate pseudovirus stocks, encodes the HIV-1 genome with firefly luciferase replacing the *env* gene)	Richman et al.^38^	Monogram proprietary vector
pCXAS (CMV promoter driven expression vector)	Richman et al.^38^	Monogram proprietary vector
pCXAS-ACE2 expression vector	Huang et al.^39^	Monogram proprietary vector
pCXAS-TMPRSS2 expression vector	Huang et al.^39^	Monogram proprietary vector
pCXAS-SARS-CoV-2-Spike expression vectors: Index D614G	Huang et al.^39^	Monogram proprietary vector
pCXAS-SARS-CoV-2-Spike expression vectors: Beta Delta Gamma Lambda Mu	This paper	Monogram proprietary vectors

**Software and algorithms**		

Luminometer	Thermo-Fischer	Luminoskan
Neutralizing antibody assay data analysis (inhibition curve fitting and ID50 determinations)	Monogram proprietary analysis software	N/A
Methodical Mind vS600MM	Meso Scale Discovery	N/A
Prism	Graphpad	Version 8.0 and higher
Excel	Microsoft	Office 365 MSO
TAYLOR (Trimming Amplicons You LOve Rapidly) pipeline	Addetia et al.^32^	https://github.com/greninger-lab/covid_swift_pipeline
Nextclade	Aksamentov et al.^33^	https://clades.nextstrain.org/
Pangolin	O’Toole et al.^34^	https://cov-lineages.org/resources/pangolin.html
Reproducible reporting workflow for statistical analyses of candidate immune correlates of risk and protection in vaccine efficacy trials, including exposure-proximal immune correlates analyses; includes multiple modular workflows for correlates of risk/protection analyses and automated reporting of analytic results	This paper^35^	https://doi.org/10.5281/zenodo.13968569
vaccine: Statistical Tools for Immune Correlates Analysis of Vaccine Clinical Trial Data (code to conduct the correlates of protection analyses based on the controlled vaccine efficacy framework)	Kenny^37^	https://doi.org/10.32614/CRAN.package.vaccine
Code to conduct sequence distance-specific correlates of risk and protection analyses	Sun et al.^13^	https://github.com/fei-heng/markPH2missing
Code to conduct the marker-thresholded correlate of protection analyses	This paper^36^	https://doi.org/10.5281/zenodo.14117624

**Other**		

Index SARS-CoV-2 sequence (MN908947.3): Source for codon-optimized version of the full-length spike gene	Index Synthetic DNA; MN908947.3	https://www.ncbi.nlm.nih.gov/nuccore/MN908947
Beta, Delta, Gamma, Lambda, Mu, and Zeta variants assayed in nAb assay (lineage designations)	Beta: synthetic DNA Delta: synthetic DNA Gamma: synthetic DNA Lambda: synthetic DNA Mu: synthetic DNA Zeta: synthetic DNA	B.1.351 B.1.617.2 P.1 (B.1.1.28.1) C.37 (B.1.1.1) B.1.621 P.2 (B.1.1.28.2)
Reference sequence for SARS-CoV-2 Spike gene sequence alignments	NC_045512.2 (index)	https://www.ncbi.nlm.nih.gov/nuccore/NC_045512.2
Plate Reader	Meso Scale Discovery	S600
Microplate Washer	BioTek	405 TS
Benchsmart	Rainin	Benchsmart96
Plate Shaker	Heidolph	Titramax 100
High-throughput instrument for automated nucleic acid purification	Roche	MagNA Pure 96 System

### Experimental model and study participant details

#### Human participants

##### Trial design and participant characteristics

The ENSEMBLE trial was a randomized, double-blind, placebo-controlled phase 3 trial of single-dose Ad26.COV2.S vaccine that randomized 44,325 participants in a 1:1 ratio, using randomly permuted blocks, to vaccine or placebo.^1^^,^^2^ The trial enrolled adults ≥ 18 years old who were in good or stable health and who did not have any coexisting conditions or who had stable, well-controlled coexisting conditions. Individuals who had previously received a COVID-19 vaccine were excluded. Further details on trial design are in the primary publications.^1^^,^^2^

##### Ethics

Informed consent was obtained from all participants. All experiments were performed in accordance with the relevant guidelines and regulations. The ENSEMBLE trial was reviewed and approved by all relevant local ethics committees and Institutional Review Boards, listed below:

Argentina: ANMAT - Administración Nacional de Medicamentos, Alimentos y Tecnologia Médica (Capital Federal, La Plata, Ramos Mejia – Buenos Aires; Ciudad Autonoma de Buenos Aires), Comite de Etica Dr Carlos Barclay (Capital Federal, Buenos Aires; Ciudad Autonoma de Buenos Aires), Comision Conjunta de Investigacion en Salud – CCIS (La Plata, Ramos Mejia - Buenos Aires), Comite de Bioetica de Fundacion Huesped (Ciudad Autonoma de Buenos Aires), Comité de Docencia e Investigación DIM Clínica Privada (Ramos Mejia, Buenos Aires), Comité de Ética en Investigación Clínica y Maternidad Suizo Argentina (Ciudad Autonoma de Buenos Aires), Comité de Ética en Investigación de CEMIC (Ciudad Autonoma de Buenos Aires), Comite de Etica en Investigacion DIM Clinica Privada (Ramos Mejia, Buenos Aires), Comite de Etica Hospital Italiano de La Plata (La Plata, Buenos Aires), Comite de Etiica en Investigacion Hospital General de Agudos J.M. Ramos Mejia (Ciudad Autonoma de Buenos Aires), Comitéde ética del Instituto Médico Platense (CEDIMP) (La Plata, Buenos Aires), IBC Fundacion Huesped (Ciudad Autonoma de Buenos Aires), IBC Helios Salud (Ciudad Autonoma de Buenos Aires), IBC Hospital General de Agudos J.M. Ramos Mejia (Ciudad Autonoma de Buenos Aires)

Brazil: ANVISA – Agência Nacional de Vigilância Sanitária (Salvador, Bahia; Barretos, Campinas, São Paulo, São Jose Rio Preto, Ribeirão Preto, São Caetano do Sul – São Paulo; Santa Maria, Porto Alegre – Rio Grande do Sul; Natal, Rio Grande do Norte; Para, Pará; Belo Horizonte, Minas Gerais; Rio de Janeiro, Nova Iguaçu – Rio de Janeiro; Curitiba, Paraná; Brasília, Distrito Federal; Campo Grande, Mato Grosso do Sul; Criciúma, Santa Catarina; Cuiabá, Mato Grosso), CONEP - Comissão Nacional de Ética em Pesquisa (Salvador, Bahia; São Paulo, São Paulo; Santa Maria, Rio Grande do Sul; Para, Pará;), CAPPESq – Comissão de Ética de Análise para Projetos de Pesquisa – HCFMUSP (São Paulo, São Paulo), CEP da Faculdade de Medicina de São José do Rio Preto – FAMERP (São Jose Rio Preto, São Paulo), CEP da Faculdade de Medicina do ABC/SP (São Paulo, São Paulo), CEP da Fundação Pio XII - Hospital do Câncer de Barretos/SP (Barretos, São Paulo), CEP da Liga Norteriograndense Contra o Câncer (Natal, Rio Grande do Norte), CEP da Pontificia Universidade Catolica de Campinas / PUC Campinas (Campinas, São Paulo), CEP da Real Benemérita Associaçao Portuguesa de Beneficência - Hospital São Joaquim (São Paulo, São Paulo), CEP da Santa Casa de Misericórdia de Belo Horizonte (Belo Horizonte, Minas Gerais), CEP da Secretaria Municipal De Saúde do Rio de Janeiro – SMS/RJ (Rio de Janeiro, Rio de Janeiro), CEP da Universidade de São Caetano do Sul (CEP da Universidade de São Caetano do Sul, São Paulo), CEP da Universidade Federal de Mato Grosso do Sul – UFMS (Campo Grande, Mato Grosso do Sul), CEP da Universidade Federal de Minas Gerais (Belo Horizonte, Minas Gerais), CEP do Centro de Referência e Treinamento DST/AIDS (São Paulo, São Paulo), CEP do do INI-Ipec/Fiocruz (Rio de Janeiro, Rio de Janeiro), CEP do Grupo Hospitalar Conceição/RS (Porto Alegre, Rio Grande do Sul), CEP do Hospital das Clínicas da Faculdade de Medicina de Ribeirão Preto/USP (Ribeirão Preto, São Paulo), CEP do Hospital de Clinicas da Universidade Federal do Parana - HCUFPR/PR (Curitiba, Paraná), CEP do Hospital de Clínicas de Porto Alegre/HCPA (Porto Alegre, Rio Grande do Sul), CEP do Hospital Geral de Nova Iguaçu (Nova Iguaçu, Rio do Janeiro), CEP do Hospital Municipal São José (Criciúma, Santa Catarina), CEP do Hospital Pró-Cardíaco/RJ (Rio de Janeiro, Rio de Janeiro), CEP do Hospital Sírio Libanês (São Paulo, Sao Paulo), CEP do Hospital Universitário Júlio Muller/MT (Cuiabá, Mato Grosso), CEP do Hospital Universitário Professor Edgard Santos – UFBA (Salvador, Bahia), CEP do Instituto de Cardiologia do Distrito Federal (Brasília, Distrito Federal), CEP do Instituto de Infectologia Emílio Ribas/SP (São Paulo, Sao Paulo), CEP do Instituto de Saude e Bem Estar da Mulher - ISBEM/SP (São Paulo, Sao Paulo), CEP em Seres Humanos do HFSE - Hospital Federal dos Servidores do Estado (Rio de Janeiro, Rio de Janeiro), CONEP - Comissão Nacional de Ética em Pesquisa (Brasília, Distrito Federal, Salvador, Bahia; Belo Horizonte, Minas Gerais; Cuiabá, Mato Grosso; Campo Grande, Mato Grosso do Sul; Nova Iguaçu, Rio Janeiro – Rio Janeiro; Barretos, Campinas, Sao Jose Rio Preto, São Caetano do Sul, Sao Paulo, Ribeirão Preto – Sao Paulo; Porto Alegre, Rio Grande do Sul; Natal, Rio Grande do Norte; Curitiba, Paraná; Criciúma, Santa Catarina)

Chile: Comité de Ética de Investigación en Seres Humanos (Santiago, Region Met), Comité Ético Científico Servicio de Salud Metropolitano Central (Santiago, Region Met), Instituto de Salud Pública de Chile (Santiago, Region Met; Talca, Temuco), Comité Ético-Científico Servicio de Salud Metropolitano Sur Oriente (Talca, Santiago), Comité de Evaluación Ética Científica Servicio de Salud Araucanía Sur Temuco (Temuco), Comité Ético Científico Servicio de Salud Metropolitano Central (Viña del Mar)

Colombia: CEI de la Fundación Cardiovascular de Colombia (Floridablanca), Comité de Ética en Investigación Clínica de la Costa (Barranquilla), INVIMA - Instituto Nacional de Vigilancia de Medicamentos y Alimentos (Colombia) (Barranquilla), Comite de Etica en Investigacion de la E.S.E. Hospital Mental de Antioquia (Santa Marta), Comite de Etica en la Investigacion CAIMED (Bogotá), INVIMA - Instituto Nacional de Vigilancia de Medicamentos y Alimentos (Colombia) (Bogotá), Comite Corporativo de Etica en Investigacion de la Fundacion Santa Fe de Bogota (Bogotá), Comité de Ética e Investigación Biomédica de la Fundación Valle del Lili (Cali), Comite de Etica e Investigacion IPS Universitaria (Medellin), Comite de Etica en Investigacion Asustencial Cientifica de Alta Complejidad (Bogotá), Comite de Etica en Investigacion Biomedica de la Corporacion Cientifica Pediatrica de Cali (Cali), Comité de Ética en Investigación Clínica de la Costa (Barranquilla), Comite de Etica en Investigacion de la E.S.E. Hospital Mental de Antioquia (Barrio Barzal Villavicencio), Comite de Etica en Investigacion del area de la Salud de la Universidad del Norte (Barranquilla), Comite de Etica en Investigacion Medplus Centro de Recuperación Integral S.A.S (Bogotá), Comité de Ética en Investigaciones CEI-FOSCAL (Floridablanca), Comite de Etica en la Investigacion CAIMED (Bogotá), Comite de Etica para Investigacion Clinica(CEIC) de la Fundacion Centro de Investigacion Clinica CIC (Medellin), Comite de Investigaciones y Etica en Investigaciones Hospital Pablo Tobon Uribe (Medellin), INVIMA - Instituto Nacional de Vigilancia de Medicamentos y Alimentos (Colombia) (Barranquilla, Bogotá, Cali, Floridablanca, Medellin.

Mexico: CEI del Hospital Civil de Guadalajara Fray Antonio Alcalde (Guadalajara, Jalisco), CEI Hospital La Mision (Tijuana, Baja California Norte), CI del Hospital Civil de Guadalajara Fray Antonio Alcalde (Guadalajara, Jalisco), CI Hospital La Mision (Tijuana, Baja California Norte), Comite de Bioseguridad del Instituto Nacional de Salud Publica (Mexico, Distrito Federal; Cuernavaca, Morelos), Comite de Etica en Investigacion del Instituto Nacional de Salud Publica (Mexico, Distrito Federal; Cuernavaca, Morelos), Comité de Bioseguridad del Hospital La Misión S.A. de C.V. (Tijuana, Baja California Norte; Oaxaca, Oaxaca; Merida, Yucatán; Tijuana, Baja California Norte), Comité de Bioseguridad de la Coordinación de Investigación en Salud (IMSS) (Mexico, Estado de Mexico), Comité de Bioseguridad de Médica Rio Mayo (CLINBOR) (Mexico, Distrito Federal), Comité de Bioseguridad del Hospital Universitario “Dr. José Eleuterio González” (Monterrey, Nuevo León), COFEPRIS (Comisión Federal para la Protección contra Riesgos Sanitarios) (Cuernavaca, Morelos; Mexico, Distrito Federal; Monterrey, Nuevo León; Oaxaca, Oaxaca; Merida, Yucatán), Comite de Etica de la Fac de Med de la UANL y Hospital Universitario “Dr. Jose Eleuterio Gonzalez” (Monterrey, Nuevo León), Comite de Etica en Investigacion de la Unidad de Atencion Medica e Investigacion en Salud S.C. (Merida, Yucatán), Comite de Etica en Investigacion de Medica Rio Mayo S.C. (Mexico, Distrito Federal), Comite de Etica en Investigacion de Oaxaca Site Management Organization, S.C. (Oaxaca, Oaxaca), Comite de Etica en Investigacion del Centro Medico Nacional Siglo XXI (IMSS) (Mexico, Estado do Mexico), Comité de Investigación de la Coordinación de Investigación en Salud (IMSS) (Mexico, Estado do Mexico), Comite de Investigacion de la Unidad de Atencion Medica e Investigacion en Salud S.C. (Merida, Yucatán), Comite de Investigacion de Oaxaca Site Management Organization, S.C. (Oaxaca, Oaxaca), Comité de Investigación del Hospital Universitario José Eleuterio González (Monterrey, Nuevo León), Comite de Investigacion Medica Rio Mayo, S.C. (Mexico, Distrito Federal)

Peru: Comite Nacional Transitorio de Etica en Invest. de los Ensayos Clinicos de la enfermedad COVID-19 (Iquitos - Maynas, Loreto; Lima, San Miguel – Lima), INS - Instituto Nacional de Salud (Peru) (Lima, San Miguel – Lima; Callao; Iquitos – Maynas, Loreto)

South Africa: Department Agriculture, Forestry and Fisheries (DAFF) (Port Elizabeth, Mthatha – Eastern Cape; Cape Town, Worcester – Western Cape; Durban, Ladysmith, Vulindlela – KwaZulu-Natal; Johannesburg, Pretoria, Mamelodi East, Soweto, Tembisa – Gauteng; Rustenburg, Klerksdorp – North West; Bloemfontein, Free State; Middelburg, Mpumalanga; Dennilton, Limpopo), Pharma Ethics (Port Elizabeth, Eastern Cape; Durban, Ladysmith – KwaZulu-Natal; Cape Town, Western Cape; Pretoria, Mamelodi East, Johannesburg, Tembisa – Gauteng; Rustenburg, Klerksdorp – North West; Bloemfontein, Free State; Middelburg, Mpumalanga; Dennilton, Limpopo), SAHPRA - South African Health Products Regulatory Authority (Port Elizabeth, Mthatha – Eastern Cape; Cape Town, Worcester – Western Cape; Durban, Ladysmith, Vulindlela – KwaZulu-Natal; Johannesburg, Pretoria, Mamelodi East, Soweto, Tembisa – Gauteng; Rustenburg, Klerksdorp – North West; Bloemfontein, Free State; Middelburg, Mpumalanga; Dennilton, Limpopo), WIRB (Mamelodi East, Pretoria – Gauteng; Ladysmith, KwaZulu-Natal; Bloemfontein, Free State; Cape Town, Western Cape; Dennilton, Limpopo), Wits Health Consortium (Soweto, Johannesburg – Gauteng; Ladysmith, KwaZulu-Natal; Mthatha, Eastern Cape), Wits Institutional Biosafety Committee (Soweto, Pretoria, Johannesburg, Tembisa – Gauteng; Rustenburg, Klerksdorp – North West; Mthatha, Eastern Cape), University of Cape Town HREC (Cape Town, Worcester – Western Cape); University of Cape Town Institute of Infectious Disease & Molecular Medicine (Cape Town, Worcester – Western Cape), University of Cape Town Institutional Biosafety Committee (Cape Town, Worcester – Western Cape), SAMRC Human Research Ethics Committee Scientific Review (Durban, KwaZulu-Natal), Sefako Makgatho University Research Ethics Committee (SMUREC) (Pretoria, Gauteng), University of KwaZulu Natal Institutional Biosafety Committee (Durban, KwaZulu-Natal), University of KwaZulu-Natal Ethics (Durban, Vulindlela – KwaZulu-Natal), University of Stellenbosch Ethics Committee (Cape Town, Western Cape), University of KwaZulu Natal Institutional Biosafety Committee (Vulindlela, KwaZulu-Natal)

United States: Advarra IBC (Detroit, MI; Chapel Hill, NC; Boston, MA; Seattle, WA; Winston-Salem, NC; Austin, TX; Peoria, IL; Huntsville, AL; Long Beach, CA; Tucson, AZ), Biomedical Institute of New Mexico - IBC (Albuquerque, NM), Birmingham VA Medical Center - Alabama- IBC (Birmingham, AL), Clinical Biosafety Services (Hollywood, FL), Columbia University IBC (New York, NY), Copernicus Group IRB (Austin, Dallas, Houston, San Antonio – TX; Rochester, New York, Bronx, Binghamton – NY; Hillsborough, Hackensack, Newark, New Brunswick – NJ; West Palm Beach, Coral Gables, Hollywood, Miami, Orlando, Gainesville, Tampa, Hallandale Beach, Pinellas Park, The Villages, Jacksonville, Deland – FL; Fort Worth, Dallas, San Antonio – TX; Norfolk, Charlottesville – VA; Matairie, New Orleans – LA; Nashville, Knoxville, Memphis, Bristol – TN; Cincinnati, Cleveland, Columbus, Akron – OH; Detroit, Ann Arbor, Grand Rapids – MI; Philadelphia, Pittsburgh – PA; Stanford, San Diego, San Francisco, Oakland, Long Beach, Anaheim, Sacramento, West Hollywood – CA, Las Vegas, Reno – NV; Chicago, Peoria – IL; Omaha, NE; Mobile, Birmingham, Huntsville – AL; St Louis, Greer, Kansas City – MO; Boston, MA; Harrisburg, SD; Decatur, Atlanta, Savannah – GA; Baltimore, Rockville, Annapolis – MD; New Haven, Hartford – CT; Chapel Hill, Raleigh, Fayetteville, Charlotte, Durham, Winston-Salem – NC; Indianapolis, Valparaiso, Evansville – IN; Seattle, WA; Aurora, CO; Lexington, Louisville – KY; Murray, West Jordan, Salt Lake City – UT; Phoenix, Tucson, Glendale – AZ; Spartanburg, Columbia, North Charleston, Anderson, Charleston, Mount Pleasant – SC; Portland, Medford, Corvallis – OR; Albuquerque, Gallup – NM; Little Rock, AR; Jackson, MS; Newport News, VA, Minneapolis, MN; Lenexa, KS), WIRB (Hackensack, NJ; Dallas, TX; Baltimore, MD; Chicago, IL; Aurora, CO; Winston-Salem, NC; Minneapolis, MN; Orlando, Miami, Gainesville – FL; Philadelphia, Pittsburgh – PA; Boston, MA; St Louis, MO; Bronx, New York, NY; New Brunswick, NJ; Phoenix, AZ; Birmingham, AL; Louisville, KY; Albuquerque, NM; New Orleans, LA; Baltimore, MD; San Francisco, CA; Tampa, FL; Aurora, CO; Columbia, SC; Decatur, GA; Reno, NV; Raleigh, NC; Little Rock, AS), Clinical Biosafety Services (Dallas, San Antonio – TX; San Diego, CA; Lexington, KY; Murray, UT; Greer, Kansas City, St Louis – MO; Rockville, MD; Las Vegas, NV; Cincinnati, Columbus, Akron – OH; Phoenix, Tucson, Glendale – AZ; North Charleston, Anderson – SC; Orlando, Pinellas Park, The Villages, Miami – FL; Birmingham, AL; Valparaiso, Evansville – IN; Lenexa, KS), Columbia University IBC (Bronx, New York), Durham VA Medical Center-IBC (Raleigh, NC), Emory University IRB (Decatur, GA), Environmental Health and Safety Office (Atlanta, GA), Institutional Biosafety Committee (New Orleans, LA), James A. Haley Veterans Hospital_IBC (Tampa, FL), Jesse Brown VA Medical Center- IBC (Chicago, IL), Mass General Brigham IBC (Boston, MA), Mount Sinai- Icahn School of Medicine IBC (New York, NY), New York Blood Center IBC (New York, NY), OHSU IBC (Portland, OR), Partners Institutional Biosafety Committee (Boston, MA), Rocky Mountain Regional VA Medical Center-IBC (Aurora, CO), Rush University Medical Center (Chicago, IL), Rush University Medical Center IBC (Chicago, IL), Rutgers Institutional Biosafety Committee (New Brunswick, NJ), Saint Louis University IBC (St Louis, MO), Saint Michael’s Medical Center IRB (Newark, NJ), Southeast Louisiana Veterans Health Care System IBC (New Orleans, LA), St. Jude Children’s Research Hospital IBC Committee (Memphis, TN), St. Jude Children’s Research Hospital IRB (Memphis, TN), Stanford University Administrative Panel on Human Subjects in Medical Research (Stanford, CA), Temple University – IBC (Philadelphia, PA), The University of Chicago Institutional Biosafety Committee (Chicago, IL), UAMS IBC (Little Rock, AS), UIC IBC (Chicago, IL), University of Alabama at Birmingham Institutional Biosafety Committee (Birmingham, AL), University of Arkansas IRB (Little Rock, AS), University of Kentucky Biological Safety (Lexington, KY), University of Kentucky IRB (Lexington, KY), University of Louisville IRB (Louisville, KY), University of Miami-IBC (Miami, FL), University of Mississippi Medical Center IRB (Jackson, MI), University of Pennsylvania Institutional Biosafety Committee (Philadelphia, PA), University of Pittsburgh IBC (Pittsburgh, Pennsylvania), University of South Florida IRB (Tampa, FL), University of Utah Institutional Biosafety Committee (Salt Lake City, UT), University of Utah IRB (Salt Lake City, UT), UTHealth – IBC (Houston, TX), VA Baltimore Research & Education Foundation (BREF)- IBC (Baltimore, MD), VA Central Arkansas Veterans Healthcare System-IBC (Little Rock, AS), VA James J. Peters Department of VA Medical Center-IBC (Bronx, NY), VA Medical Center - Atlanta-IBC (Decatur, GA), VA Medical Center San Francisco- IBC (San Francisco, CA), VA North Florida/South Georgia IBC (Gainesville, FL), VA North Texas Health Care System IBC (Dallas, TX), VA San Diego Healthcare System IBC (Phoenix, AZ), VA Sierra Nevada Health Care System-IBC (Reno, NV), Vanderbilt University Instituitional Review Board (Nashville, TN), Washington University IBC (St Louis, MO), WCG IBCS (Houston, TX; Orlando, FL), Western Institutional Review Board (San Diego, CA; Detroit, MI; New Orleans, LA; New York, NY), WIRB - IBCS Services (Chicago, IL; New Orleans, LA; Oakland, CA; Minneapolis, MN; Columbus, OH; Lexington, KY), WJB Dorne VA Medical Center IBC (Columbia, SC).

##### Immunogenicity subcohort characteristics

Information on participant age, sex, ethnicity, and race for baseline SARS-CoV-2 seronegative per-protocol trial participants in the immunogenicity subcohort is provided in Table S2 (all geographic regions pooled), Table S3 (Latin America), Table S4 (South Africa), and Table S5 (United States) in Carpp, Hyrien, and Fong et al.^40^

In the immunogenicity subcohort (all regions pooled), 416 participants were female and 514 were male; mean age was 55.6 years (range 18.0, 90.0 years). In the Latin America subset, 91 participants were female and 154 were male; mean age was 55.3 years (range 19.0, 88.0 years). In the South Africa subset, 109 participants were female and 112 were male; mean age was 56.1 years (range 21.0, 84.0 years). In the United States subset, 216 participants were female and 248 were male; mean age was 55.6 years (range 18.0, 90.0 years). See Tables S2–S5 in Carpp, Hyrien, and Fong et al.^40^

Data on participant sex was self-reported, solicited and collected by predefined options.

##### Post hoc analyses by sex

Two post hoc analyses were performed to investigate the potential influence (or association) of sex on the results of the study. Figure S12 shows violin plots of D29 nAb-ID50 titers against each breakthrough case-matched lineage compared to against the same lineage in non-cases, shown separately in Latin America males and females. In males, neutralizing antibody titers against each of Reference, Mu, Lambda, Zeta, and Gamma are shown for 150 non-cases, and against Reference, Mu, Lambda, Zeta, and Gamma for 35, 24, 27, 8, and 41 lineage-matched cases. In females, neutralizing antibody titers against each of Reference, Mu, Lambda, Zeta, and Gamma are shown for 94 non-cases, and against Reference, Mu, Lambda, Zeta, and Gamma for 18, 13, 16, 10, and 30 lineage matched cases. Table S3 shows hazard ratio point estimates and 95% confidence intervals of lineage-specific COVID-19 per 10-fold increase in D29 lineage-specific nAb-ID50 or nAb-ID50 Reference, separately in Latin America males and females.

#### Cell lines

The pseudovirus neutralization assay used the HEK 293 cell line,^41^ sourced from the Master Cell Bank (LC0027490) established by Monogram Biosciences in 2001. No formal authentication was performed. The HEK293 cell line has been in continuous use at Monogram Biosciences since 1996 and Mycoplasma testing is routinely performed per Monogram Standard Operating Procedure. Cells were grown at 37°C in an incubator with 7% CO_2_. The HEK 293 cell line was derived from a human fetus, with Lin et al.^42^ having reported evidence that HEK 293 cells are of female provenance (i.e. the complete absence of any Y-chromosome-derived sequence in high-coverage genomic sequencing data). It is unknown whether different results would be obtained if a HEK cell line derived from a male fetus were to be used in the pseudovirus neutralization assay.

### Method details

#### Binding antibody assay

Serum anti-Spike bAbs were quantitated using a Meso Scale Discovery (MSD) assay with variant Spike antigens. This multiplex serology assay uses a V-PLEX SARS-CoV-2 Panel 19 (IgG) Kit (MSD) to detect antibodies to the following Spike antigens: Index (GenBank accession number: MN908947), Gamma (P.1), Lambda (C.37), Mu (B.1.621), Beta (B.1.351), Delta (AY.1), Delta (AY.2), Delta (AY.12), Delta (B.1.617.2), Delta (B.1.617.2; AY.4). The IgG readouts to the 5 Delta strains were combined into a single maximal signal diversity weighted^43^ Delta cross-reactivity score (Delta-score).

The Panel 19 MSD assay was performed according to the manufacturer’s instructions and all reagents were provided with the kit (K15540U). Microplate washing was performed with a BioTek 405TS instrument. In brief, for the assay, all plates, diluents, reagents, and samples were equilibrated to room temperature before loading into the plates. Blocker A solution was added and plates were incubated for at least 30 minutes. After washing, samples, calibrators, and controls were added. Plates were incubated for 2 hours and washed. Detection Antibody solution was added, after which plates were incubated for 1 hour and washed. Read Buffer was then added and plates were read on a Meso Sector S 600MM ultra-sensitive plate imager (with Methodical Mind software). A current is applied to the plate and areas of well surface which form a full complex of capture antigen/anti-spike antibodies/Sulfo-tag detection antibody will emit light in the presence of the enhanced chemiluminescence (ECL) substrate. The MSD Sector instrument quantitates the amount of light emitted and reports this ECL unit response as a result for each sample and standard of the plate. Assuming the standard and test material are biologically similar, the responses generated from both materials can be compared to quantitate the concentration of antibody in the test samples. Results were presented as dilution-adjusted interpolated values from a standard curve with assigned AU/ml. GraphPad Prism (version 8.0 and higher) and Microsoft Excel (Office 365 MSO) were used for data analysis.

Next, index-strain assay readouts were converted to the WHO 20/136 anti-SARS-CoV-2 immunoglobulin international standard^44^ scale by multiplication by the conversion constant 0.009 as described in section 2 in the SAP of Gilbert et al.^45^ While readouts to non-Index strains could not be converted to International Units (IUs), they were multiplied by the same conversion constant (0.009) as for the Index-strain readouts to create a comparable scale (as in Fong et al., submitted), and reported as AU/ml (equal to BAU/ml for the Index strain). With the exception of Spike IgG Index, strain-specific LLOQs were used to define quantifiable IgG concentration (Table S6), with values below the LLOQ assigned LLOQ/2. For Spike IgG Index, values below the positivity cut-off (Pos.Cut) were assigned Pos.Cut/2, for harmonization with the previous Index-strain correlates analyses.^5^^,^^9^^,^^45^^,^^46^^,^^47^ Strain-specific ULOQs were applied (Table S6), with values above the ULOQ assigned ULOQ.

#### Neutralizing antibody assay

Neutralizing antibodies were measured using the PhenoSense SARS CoV-2 Assay (Monogram Biosciences). This assay was derived from a recombinant virus assay initially developed to quantitate HIV antiretroviral drug resistance^48^ and later adapted to measure HIV-targeting neutralizing antibody responses in plasma samples from people living with HIV.^38^ The PhenoSense SARS CoV-2 Assay was validated to CLIA/CAP standards in 2020 and detailed methods have been published.^39^ In the current study the assay was performed using lentiviral particles pseudotyped with full-length SARS-CoV-2 Spike of the following lineages: D614G (Reference; Index strain MN908947.3 with the D614G mutation), Beta (B.1.351), Delta (B.1.617.2), Gamma [P.1 (B.1.1.28.1)], Lambda [C.37 (B.1.1.1)], Mu (B.1.621), and Zeta [P.2 (B.1.1.28.2)]. Expression constructs were made using synthetic DNA. The performance of each of the Spike variant pseudoviruses was qualified prior to routine testing (records on file in the Monogram Document Control System). Lentiviral particles were produced in HEK 293 cells (Master Cell Bank established by Monogram Biosciences, LC002749) using calcium phosphate co-transfection of spike plasmid (pCXAS-SARS-CoV-2-D614G or an equivalent plasmid expressing a variant spike of Beta, Delta, Gamma, Lambda, Mu) and lentiviral backbone plasmid (F-lucP.CNDOΔU3,^38^ which encodes the HIV-1 genome with firefly luciferase replacing the *env* gene). Pseudovirus stock was collected at 2 days post-transfection, filtered, and frozen in single-use aliquots at  < − 70°C. Human serum samples were heat-inactivated for 60 min at 56°C prior to assay. Assays were performed in white 96-well plates by incubating pseudovirus with 10 serial threefold dilutions of serum samples for one hour at 37°C. Next, cell suspension (HEK293 expressing ACE2 and TMPRSS2, transfected 24 h prior to assay day with expression plasmids pCXAS-ACE2 and pCXAS-TMPRSS2, generated using the pCXAS CMV promoter-driven expression vector^38^) was added to the serum-virus mixtures (10,000 cells per well) and assay plates were incubated at 37°C in 7% CO2 for 3 days. Luciferase activity was quantitated on assay read day through the addition of Steady Glo reagent (Promega) to each well. After a brief incubation, luciferase signal (relative luminescence units, RLUs) was measured using a luminometer (Thermo-Fischer Luminoskan). Neutralization titers represent the inhibitory dilution (ID) of serum samples at which RLUs were reduced by 50% (ID50) compared to virus control wells (no serum wells). Data analysis (inhibition curve fitting and ID50 determination) was done using Monogram proprietary analysis software.

nAb-ID50 values against Reference were converted to IUs (IU50/ml) as described.^45^ While nAb-ID50 values against other strains cannot be converted to IUs, they were multiplied by the same conversion constant (0.0653) to create a comparable scale (as in Fong et al., submitted), and reported as AU/ml, which equate to IU50/ml for the Reference strain. The limit of detection (LOD) for all strains was 2.612 AU/ml (= IU50/ml for Reference), which was used to define detectable responses. nAb-ID50 values below LOD were assigned LOD/2. ULOQs varied by strain; the minimum ULOQ = 844.7208 AU/ml (in IU50/ml for Reference) was applied to all strains, assigning values above the minimum ULOQ to the minimum ULOQ.

#### SARS-CoV-2 sequencing and lineage assignment

Next-generation sequencing of SARS-CoV-2 Spike from COVID-19 endpoint cases in the ENSEMBLE trial and variant typing based on Spike amino acid sequences has already been described,^3^ with additional details in refs.^1^^,^^49^ Sequencing was performed at the Virology Laboratory at the University of Washington, Department of Laboratory Medicine and Pathology.

In brief, RNA was extracted from nasal swab specimens from SARS-CoV-2 RT-PCR confirmed cases using a MagNA Pure 96 System (Roche). Next, 11 μL of extracted RNA was incubated with 1μL 50mM random hexamer (Thermo Fisher) and 1μL 10mM dNTP (Thermo Fisher) at 65°C for 5 min and cooled to 10°C. A SuperScript IV First-Strand Synthesis System kit (Thermo Fisher) was for generation of cDNA. SARS-CoV-2 Spike sequences were obtained using a Swift Biosciences Swift Normalase Amplicon Panel (SNAP) (then Catalog # CovG1 V2-96, now xGen SARS-CoV-2 Amplicon Panel) using workflow version 2.0 on Illumina platforms. Genome assembly was done using the TAYLOR (Trimming Amplicons You LOve Rapidly) pipeline.^32^ Lineage assignment was done using Nextclade^33^ and Pangolin.^34^

### Quantification and statistical analysis

#### Statistical analysis

##### Graphical descriptive analyses in vaccine recipients

Scatterplots of D1 and D29 antibody marker data from immunogenicity subcohort participants were generated and annotated with Spearman rank correlations. See Figures S3–S8.

##### Correlates of risk in vaccine recipients

Cox proportional hazards models were applied for univariable or multivariable D29 marker lineage-specific hazard ratio estimation with adjustment for baseline risk score, using cause-specific hazards.^50^ D29 marker-specific cumulative incidence of lineage-specific COVID-19 through to a fixed follow-up time (noted in Results) marginalizing over the distribution of baseline risk score was calculated based on Cox model fits and g-computation (see SAP,^12^ Section 13.2). Cumulative incidence for j-lineage COVID-19 by a time t_0_ is the probability that the first COVID-19 event happens by time t_0_ and this first event is with lineage j. Code to conduct the above correlate of risk analyses is available at Zenodo.^35^ D29 marker-specific thresholded (above a fixed value) cumulative incidence of lineage-specific COVID-19 marginalizing over the distribution of baseline risk score was calculated with targeted minimum loss-based estimation (see SAP,^12^ Section 13.4). Code to conduct the marker-thresholded correlate of risk analyses is available at https://github.com/Larsvanderlaan/npthreshold/tree/main/R. See also Table S2.

##### Correlates of protection

The controlled vaccine efficacy statistical framework is reviewed in ref.^6^ Lineage-specific COVID-19 controlled vaccine efficacy (CVE) curves as a function of each D29 antibody marker were estimated as one minus the marginalized marker-specific cumulative incidence of lineage-specific COVID-19 in vaccine recipients divided by the marginalized cumulative incidence of lineage-specific COVID-19 in placebo recipients (not using D29 marker data) estimated by a cause-specific Cox model^50^ marginalizing over the baseline risk score. For each pair of qualifying lineages (j, j’) in Latin America, point and 95% pointwise confidence interval estimates were generated for pairwise contrasts in CVE curves: CVEcontrast(j, j’; s) = (1 − CVE(j; s))/(1 − CVE(j’; s)). An estimate of CVEcontrast(j, j’; s) curves being near one at a given value s supports the variant-invariant CoP model at antibody level s comparing the two lineages j and j’. The model would perfectly hold if CVEcontrast(j, j’; s) = 1 across all s values and all lineage pairs (j, j’): our analysis seeks to understand ranges of s, and the lineage pairs, for which the model is supported or refuted. For hypothesis testing of whether CVEcontrast(j, j’; s) departs from one, we report bootstrap-based 95% pointwise confidence intervals at four specified s values unquantifiable/undetectable, just barely quantifiable/detectable, a central value, and a high value. Code to conduct the above correlates of protection analyses based on the controlled vaccine efficacy framework is provided in the vaccine R package, publicly available on CRAN.^37^

In addition, point and 95% confidence interval estimates, calculated with targeted minimum loss-based estimation (TMLE) (SAP,^12^ Section 13.4), are produced for lineage-specific D29 marker thresholded vaccine efficacy curves. This parameter is one minus the ratio of the marginalized thresholded cumulative incidence of lineage-specific COVID-19 (as described above) and the marginalized cumulative incidence of lineage-specific COVID-19 in placebo recipients estimated by TMLE. Unlike the Cox model analyses, those based on TMLE are valid in a nonparametric model where the proportional hazards assumption may not hold. This added robustness can come at the cost of wider confidence intervals, though these intervals are the narrowest possible among methods that achieve valid coverage in this larger model.^51^ Code to conduct the marker-thresholded correlate of protection analyses is publicly available.^36^ See also Table S2.

##### Sequence distance-specific correlates of risk and protection

The sieve analysis of ENSEMBLE showed that VE significantly decreased with several metrics for comparing Spike amino acid sequences of COVID-19 endpoints to the vaccine-strain sequence.^3^ To assess whether D29 nAb-ID50 against the Reference strain was a stronger inverse CoR of COVID-19 with viruses closer to the vaccine strain, including all COVID-19 endpoints regardless of lineage, we applied the two-phase sampling virus-distance-specific proportional hazards method^52^ that was previously applied to HIV-1^13^^,^^53^^,^^54^ and dengue phase 3 vaccine trials.^13^ Physico-chemical weighted Hamming distances in whole Spike and in whole RBD were studied, as each distance strongly associated with VE in the sieve analysis [family-wise error-rate (FWER) adjusted p < 0.001].^3^ We also studied each antibody epitope escape distance that significantly modified VE in the sieve analysis (FWER p < 0.001), two defined by Deep Mutational Scanning (DMS)^55^ and one by the Protein Data Bank (PDB) (labeled DMS, DMS7, PDB8 defined in Tables S12 and S15 of Magaret et al.^3^). For each of the five distances, the distance-specific hazard ratio (HR) per 10-fold increment in the D29 nAb-ID50 level was estimated across the range of distances, with pointwise 95% CIs and a one-sided p-value for testing whether the HR attenuated toward one with increasing viral distance. Moreover, we computed estimates of ratios of the distance-specific cumulative incidence rate function^13^ for 50^th^ percentile vs. undetectable and 90^th^ percentile vs. undetectable nAb-ID50 values, and point and 95% CI estimates of distance-specific nAb-ID50-conditional VE based on the virus-distance-specific proportional hazards model (SAP^12^ Section 14). Code to conduct these sequence distance-specific correlates analyses has been previously described.^13^ See also Table S2.

##### Missing viral lineages

To accommodate missing lineages for some COVID-19 outcome cases, each statistical method was implemented using hotdeck multiple imputation.^13^ This approach identifies for each COVID-19 endpoint with a missing lineage the 5 nearest neighbor endpoints with observed lineages, with nearest neighborhoods defined by z-scores of the calendar time of COVID-19 event, D29 nAb-ID50, and log10 viral load. Moreover, nearest neighborhoods are matched on randomized treatment arm and by coarsened geographic region (Latin America Colombia, Latin America not Colombia, South Africa, United States). Ten data sets with complete lineage information were generated. Each method was applied to each data set, and results aggregated using Rubin’s standard rules for multiple imputation to obtain confidence intervals and p-values.

##### Exposure-proximal correlates of vaccine efficacy modeling

For exposure-proximal immune correlates analyses among baseline seronegative per-protocol participants from Latin America, a regression calibration-based approach was adopted as described.^18^ A hazards model was considered for time to event specific to a given lineage (Ancestral, Lambda, Gamma, or Mu COVID-19):

(Equation 1) λ(s) = λ _0_ (s) exp (Z [β0 + *β*_1_*x* (*s* - *τ*)] + β2W) I(τ < s),

where s is calendar time (number of days since first person enrolled), τ is the number of days between first person enrolled and ∼peak (D29), Z is treatment indicator (0 and 1 for the placebo and vaccine arm, respectively), x(t) is the true underlying antibody marker at time t post-peak (in a hypothetical set-up where the immunoassay was conducted on serum samples drawn on every day of follow-up), and W is the baseline risk score. For each antibody marker against the ancestral strain (nAb-ID50 Reference and IgG Spike Index), a linear mixed effects model was used to model the antibody marker trajectory over time, with fixed effect for time since D29, age, sex and random intercept and slope (for time since D29) for individuals, including the case-cohort sampling weights. As these analyses restricted to data during the blinded phase, the linear mixed effects model with random intercepts and slopes is based on three time points: D29, D71, and M6. Only participants with two or three measurements contributed to model fitting (measurements from n=901 participants were used to fit the bAb Spike trajectory model and from n=266 participants were used to fit the nAb-ID50 trajectory model) (Figure S18). Based on the linear mixed effects model fit, the expected value of the antibody marker at every day post D29 was estimated conditional on age, sex, and observed history of antibody marker measures. The antibody trajectories estimated based on nAb-ID50 Reference and IgG Spike Index are applied to the corresponding antibody markers for Lambda, Gamma, and Mu COVID-19, using D29 variant antibody levels. Cox model parameters were estimated by maximizing the partial likelihood based on the induced hazard. The instantaneous-hazard vaccine efficacy curve conditional on the antibody marker at its current value x, VE(x) = 1 − exp(β0 + β1x), was then estimated based on the β estimates. The nonparametric bootstrap with 500 samples was used to construct the 95% pointwise confidence interval for VE(x). Code to conduct the exposure-proximal immune correlates analyses is available at Zenodo.^34^

### Additional resources

Clinical trial registry number for the ENSEMBLE trial: NCT04505722.

ClinicalTrials.gov website for the ENSEMBLE trial: https://clinicaltrials.gov/study/NCT04505722.

Protocol for the ENSEMBLE trial: https://www.nejm.org/doi/suppl/10.1056/NEJMoa2117608/suppl_file/nejmoa2117608_protocol.pdf.

(Provided with Sadoff et al.,^2^ https://doi.org/10.1056/NEJMoa2117608).

## Supplemental information

Document S1. Figures S1–S18 and Tables S1–S6

Data S1. PDF with names and affiliations of the Immune Assays Team, the Coronavirus Vaccine Prevention Network (CoVPN)/ENSEMBLE Team, and the United States Government (USG)/CoVPN Biostatistics Team
