# Supplementary material for: Immune correlates analysis of antibody responses against SARS-CoV-2 variants in the ENSEMBLE vaccine efficacy trial
Source: iScience. 2025 Sep 29;28(11):113660. doi: 10.1016/j.isci.2025.113660 (PMC12589887; doi:10.1016/j.isci.2025.113660)
Supplement: Document S1. Figures S1–S18 and Tables S1–S6 [file mmc1.pdf]

iScience, Volume *28*

Supplemental information

Immune correlates analysis of antibody responses against SARS-CoV-2 variants in the ENSEMBLE vaccine efficacy trial

Alex Luedtke, Youyi Fong, Lars van der Laan, Fei Heng, Ying Huang, Yiwen Lu, Chenchen Yu, Lindsay N. Carpp, Sanne Roels, Mathieu Le Gars, Griet A. Van Roey, Daniel J. Stieh, Ilse Van Dromme, Avi Kenny, Marco Carone, Ollivier Hyrien, Victor Ayala, Lakshmi Jayashankar, Flora Castellino, Obrimpong Amoa-Awua, Manjula Basappa, Britta Flach, Bob C. Lin, Christopher Moore, Mursal Naisan, Muhammed Naqvi, Sandeep Narpala, Sarah O'Connell, Allen Mueller, Leo Serebryannyy, Mike Castro, Jennifer Wang, Gabrielle Dziubla, April K. Randhawa, Michele P. Andrasik, Jenny Hendriks, Carla Truyers, Frank Struyf, Hanneke Schuitemaker, Macaya Douoguih, James G. Kublin, Lawrence Corey, Kathleen M. Neuzil, Linda-Gail Bekker, Nigel Garrett, Sandra W. Cardoso, Patrice DelaFontaine, Craig A. Magaret, Johan Vingerhoets, Martin Casapia, Marcelo H. Losso, Susan J. Little, Aditya Gaur, Edith Swann, Christos J. Petropoulos, Adrian B. McDermott, Jerald Sadoff, Glenda E. Gray, Beatriz Grinsztejn, Paul A. Goepfert, Dean Follmann, Pavitra Roychoudhury, Alexander L. Greninger, Richard A. Koup, Ruben O. Donis, Peter B. Gilbert, on behalf of the Immune Assays Team, the Coronavirus Vaccine Prevention Network (CoVPN)/ENSEMBLE Team, and the United States Government (USG)/CoVPN Biostatistics Team

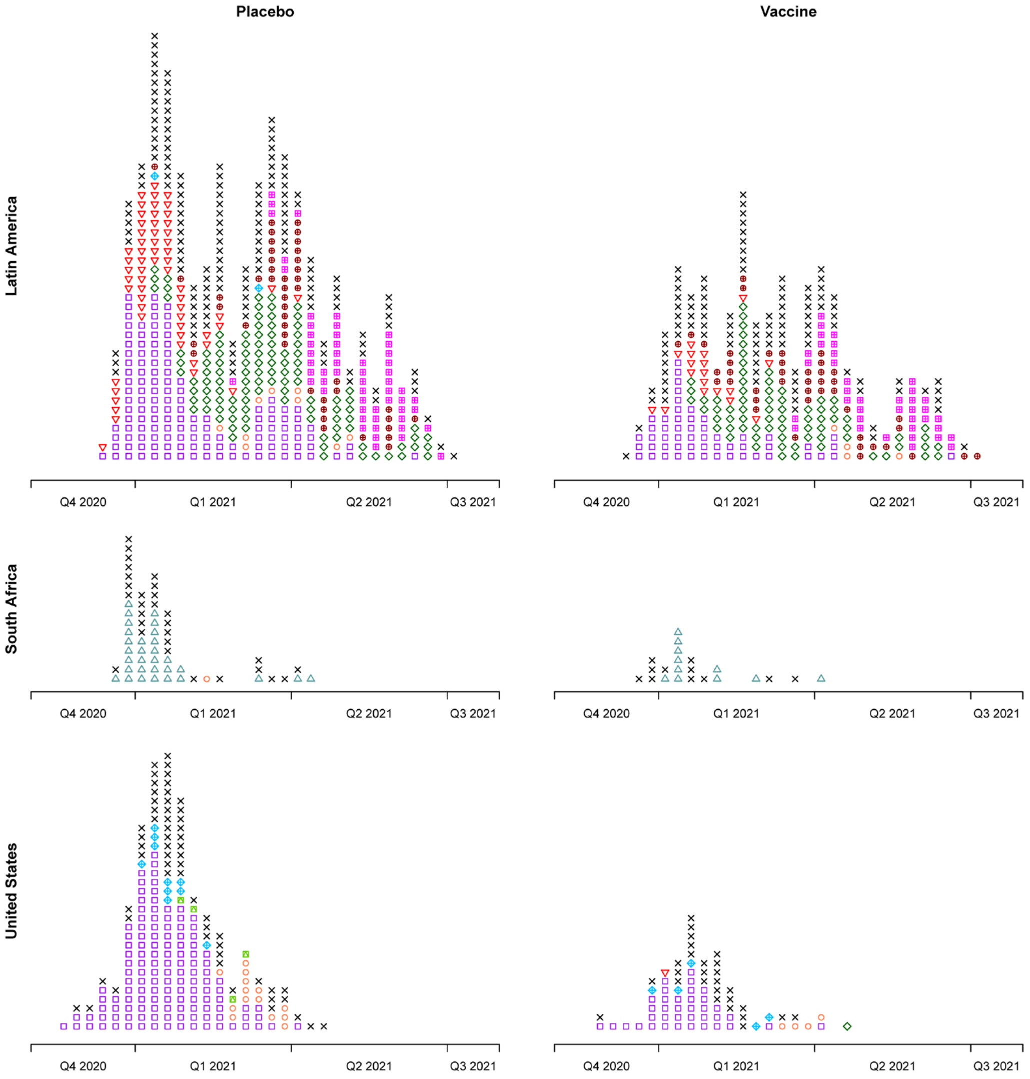

	Placebo	Vaccine	All
Ancestral	131 (23.4%)	54 (16.5%)	185 (20.9%)
Alpha	9 (1.6%)	4 (1.2%)	13 (1.5%)
Gamma	110 (19.7%)	73 (22.3%)	183 (20.7%)
Zeta	59 (10.6%)	18 (5.5%)	77 (8.7%)
Epsilon	2 (0.4%)	0 (0%)	2 (0.2%)
Lambda	45 (8.1%)	43 (13.1%)	88 (9.9%)
Mu	56 (10%)	38 (11.6%)	94 (10.6%)
Missing	147 (26.3%)	97 (29.7%)	244 (27.5%)
**Total**	559 (63.1%)	327 (36.9%)	886

	Placebo	Vaccine	All
Alpha	1 (1.6%)	0 (0%)	1 (1.2%)
Beta	31 (50.8%)	11 (50%)	42 (50.6%)
Missing	29 (47.5%)	11 (50%)	40 (48.2%)
**Total**	61 (73.5%)	22 (26.5%)	83

	Placebo	Vaccine	All
Ancestral	129 (60%)	37 (49.3%)	166 (57.2%)
Alpha	14 (6.5%)	4 (5.3%)	18 (6.2%)
Gamma	0 (0%)	1 (1.3%)	1 (0.3%)
Zeta	0 (0%)	1 (1.3%)	1 (0.3%)
Iota	4 (1.9%)	0 (0%)	4 (1.4%)
Epsilon	10 (4.7%)	5 (6.7%)	15 (5.2%)
Missing	58 (27%)	27 (36%)	85 (29.3%)
**Total**	215 (74.1%)	75 (25.9%)	290

Figure S1. SARS-CoV-2 variants causing the COVID-19 endpoints, shown by calendar date of COVID-19 occurrence, broken out by geographic region (top, Latin America; middle, South Africa; bottom, United States) and treatment assignment, Related to Table 1. Endpoint counts do not require having D1 and D29 antibody marker data.

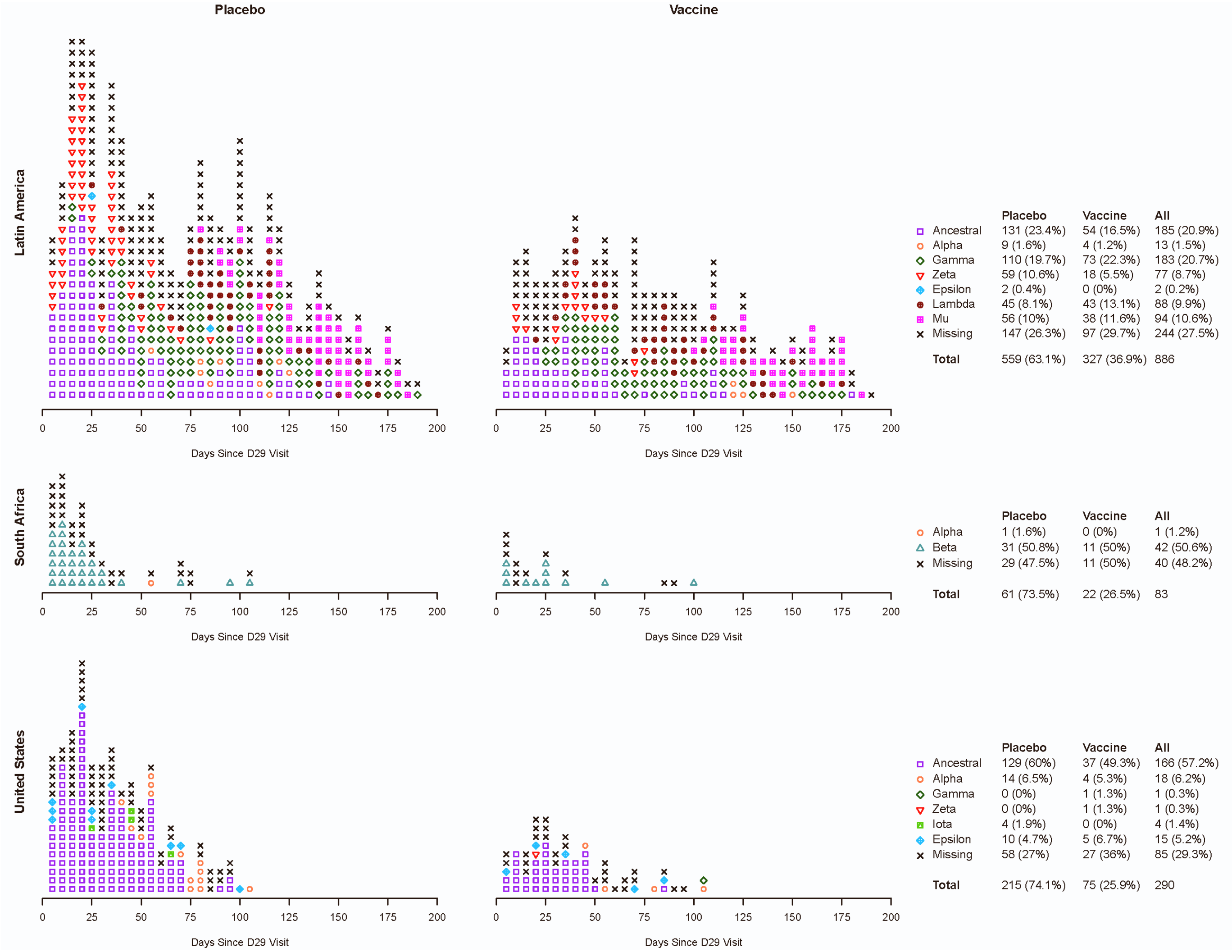

Figure S2. SARS-CoV-2 variants causing the COVID-19 endpoints, shown by numbers of days since the Day 29 visit until COVID-19 endpoint occurrence, broken out by geographic region (top, Latin America; middle, South Africa; bottom, United States) and treatment assignment, Related to Table 1. Endpoint counts do not require having D1 and D29 antibody marker data.

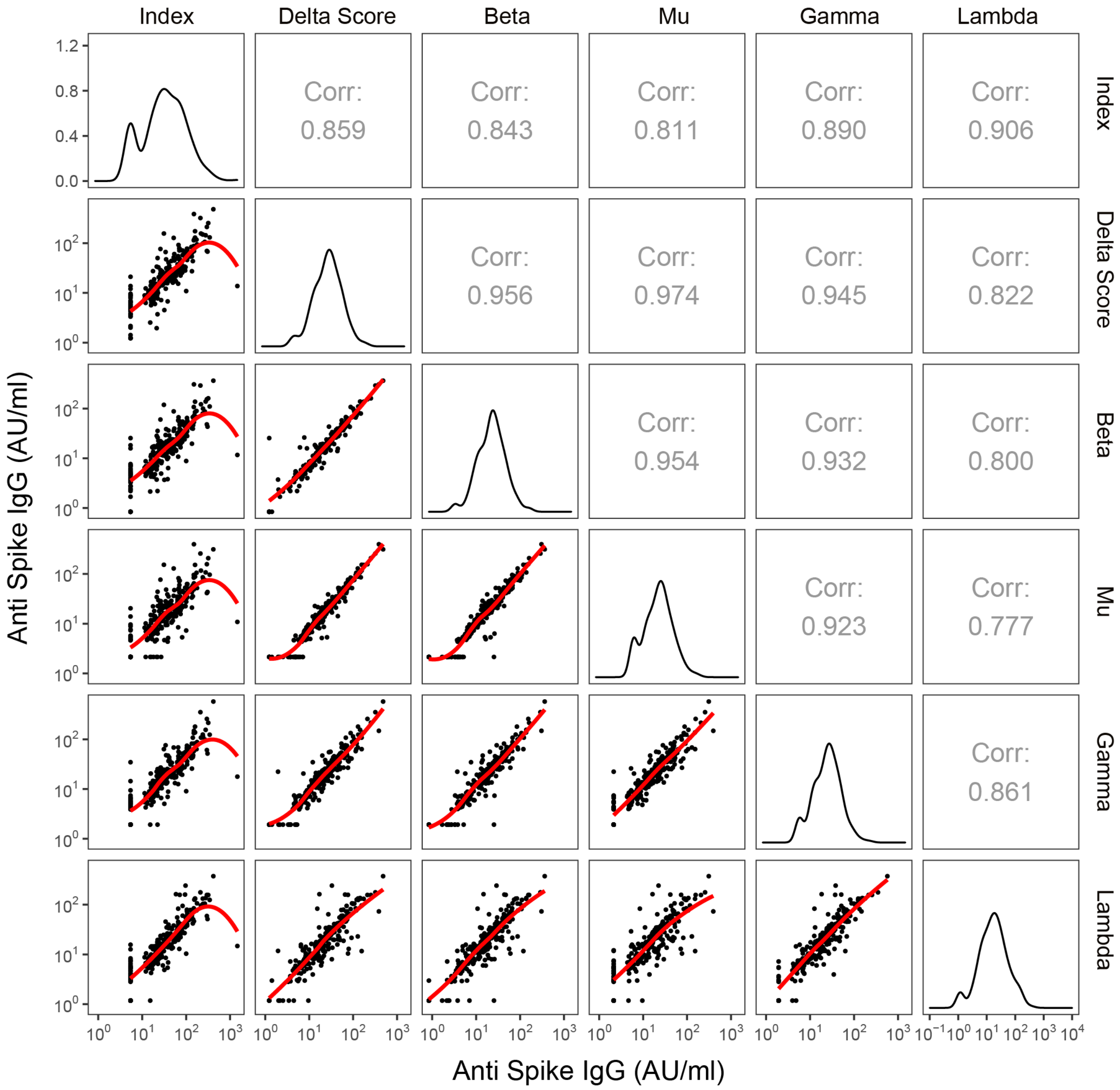

Figure S3. Pairwise scatterplots of D29 Spike IgG concentration against the antigen panel of circulating lineages in Latin America based on Latin American vaccine recipients, Related to STAR Methods. Corr = covariate-adjusted Spearman rank correlation.

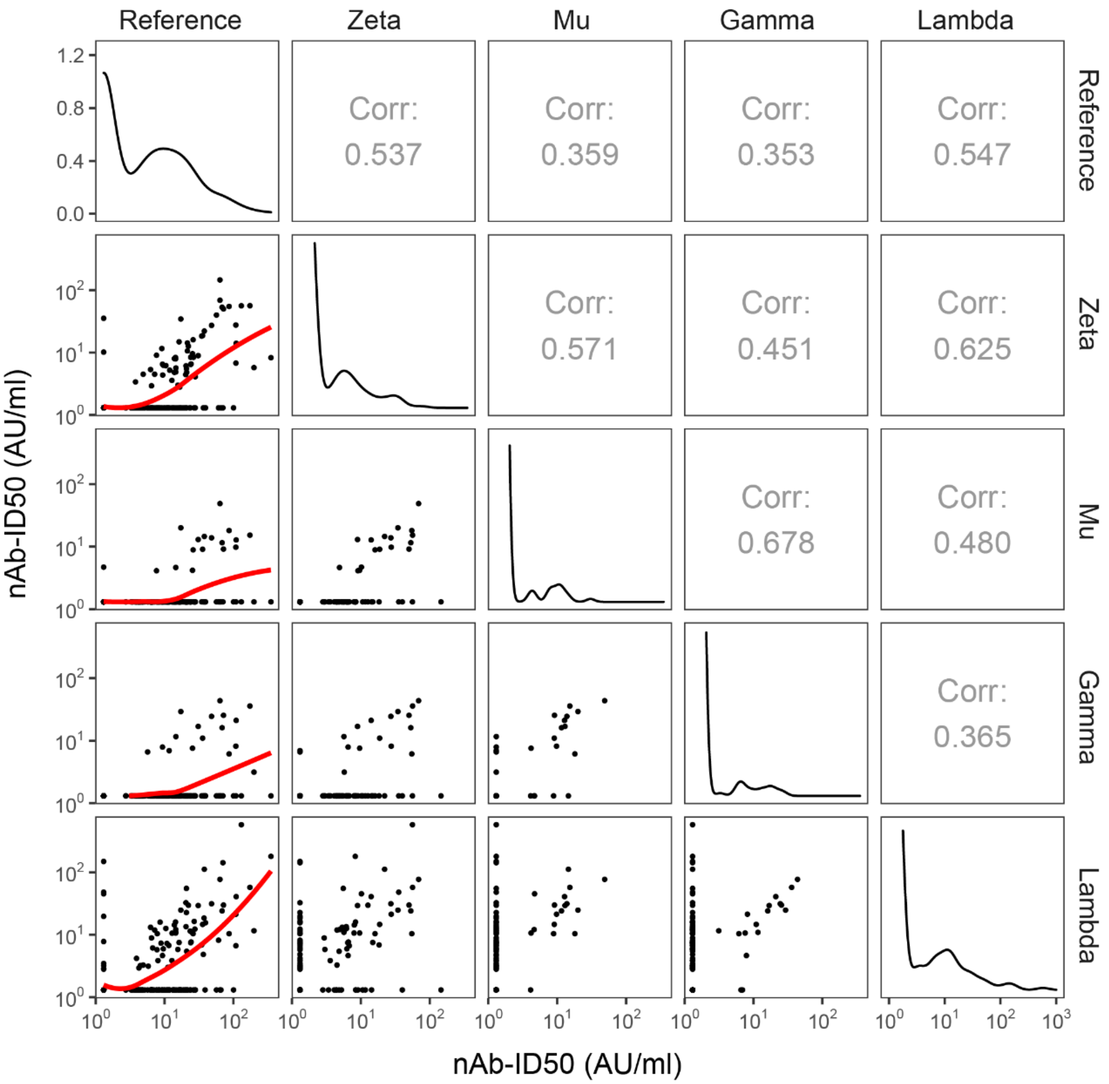

Figure S4. Pairwise scatterplots of D29 nAb-ID50 titers against the antigen panel of circulating lineages in Latin America based on Latin American vaccine recipients, Related to STAR Methods. Corr = covariate-adjusted Spearman rank correlation.

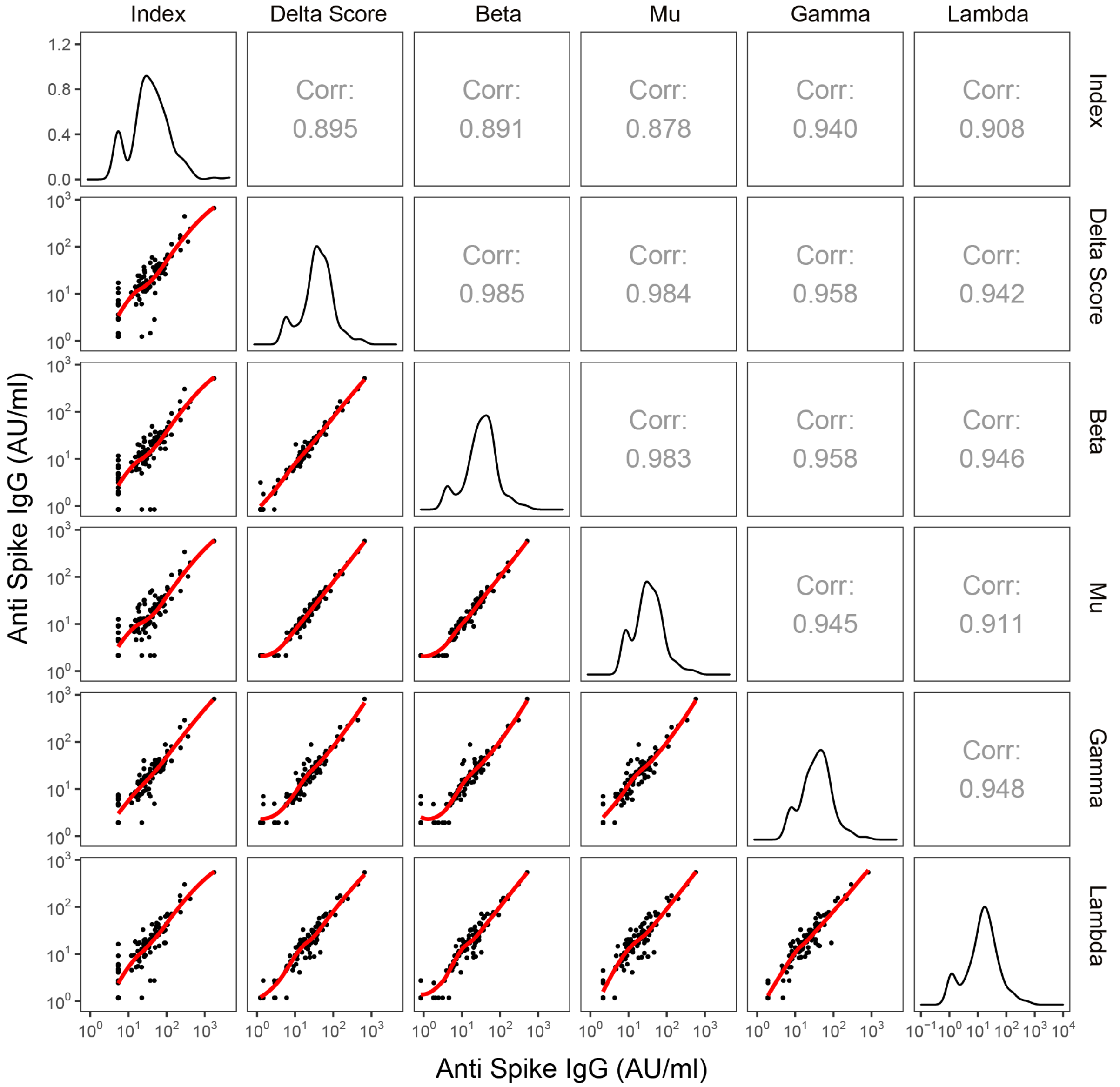

Figure S5. Pairwise scatterplots of D29 Spike IgG concentration against the antigen panel of circulating lineages in South Africa based on South Africa vaccine recipients, Related to STAR Methods. Corr = covariate-adjusted Spearman rank correlation.

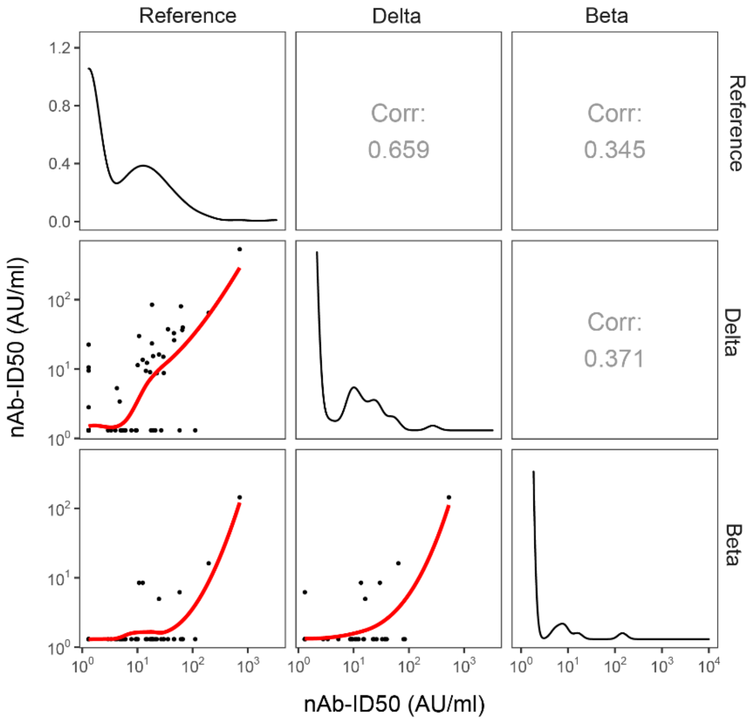

Figure S6. Pairwise scatterplots of D29 nAb-ID50 titers against the antigen panel of circulating lineages in South Africa based on South Africa vaccine recipients, Related to STAR Methods. Corr = covariate-adjusted Spearman rank correlation.

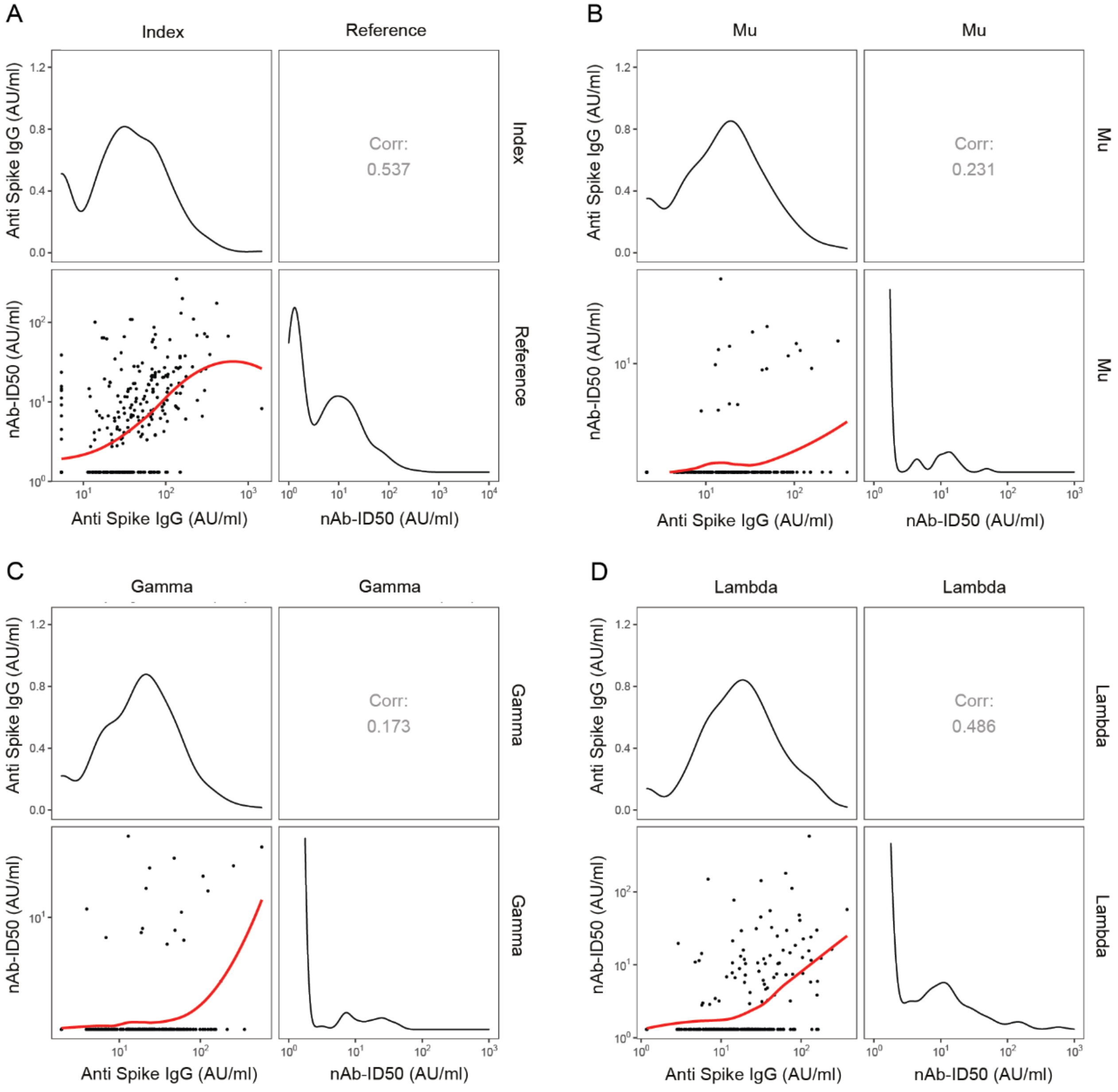

Figure S7. For each common antigen across the two assays (binding and neutralizing antibodies), pairwise scatterplots of D29 Spike IgG concentration vs. D29 nAb-ID50 titer in Latin America based on Latin America vaccine recipients, Related to STAR Methods. A) Index/Reference, B) Mu, C) Gamma, D) Lambda. Corr = covariate-adjusted Spearman rank correlation.

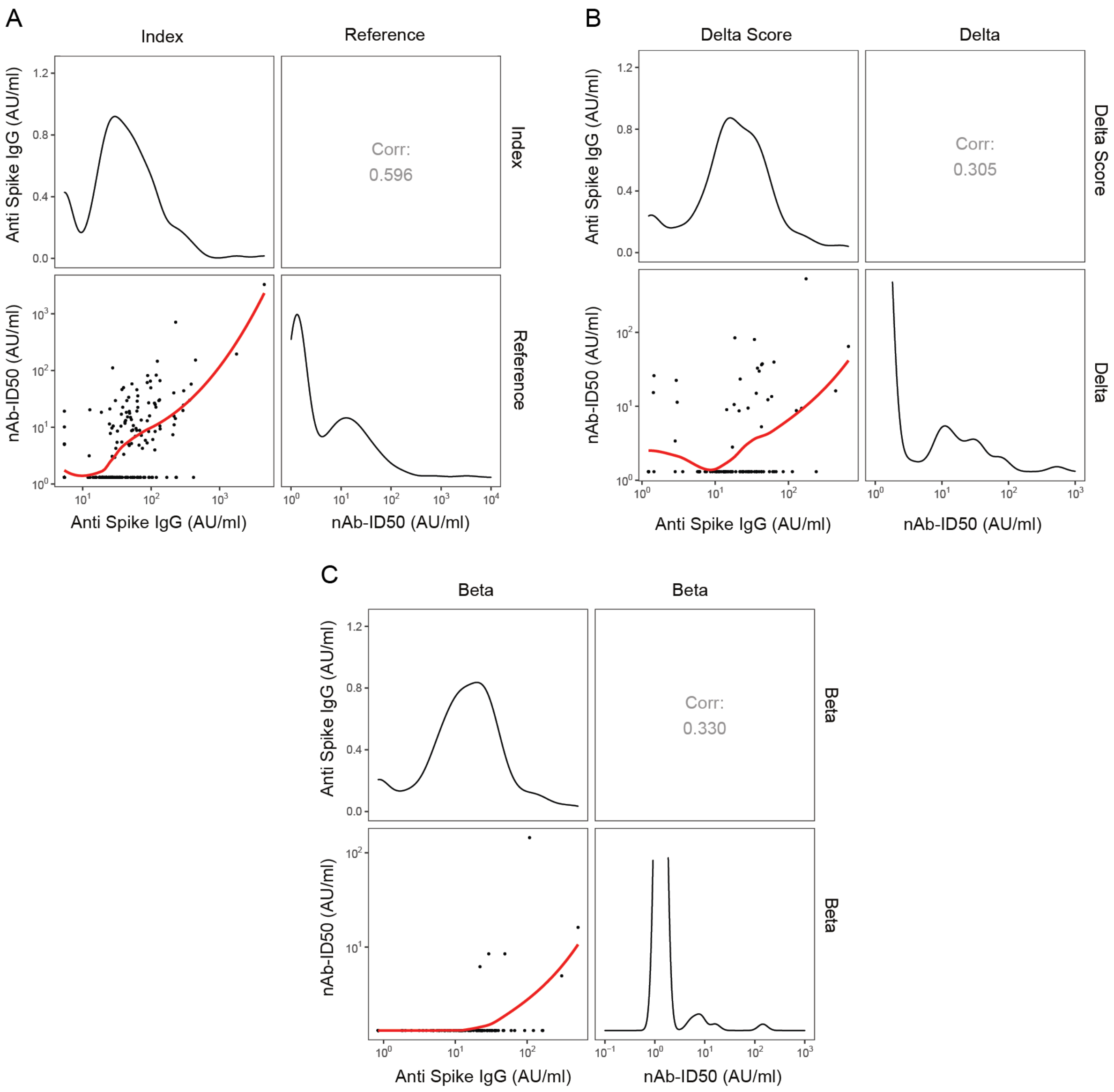

Figure S8. For each common antigen across the two assays (binding and neutralizing antibodies), pairwise scatterplots of D29 Spike IgG concentration vs. D29 nAb-ID50 titer in South Africa based on South Africa vaccine recipients, Related to STAR Methods. A) Index/Reference, B) Delta, C) Beta. Corr = covariate-adjusted Spearman rank correlation.

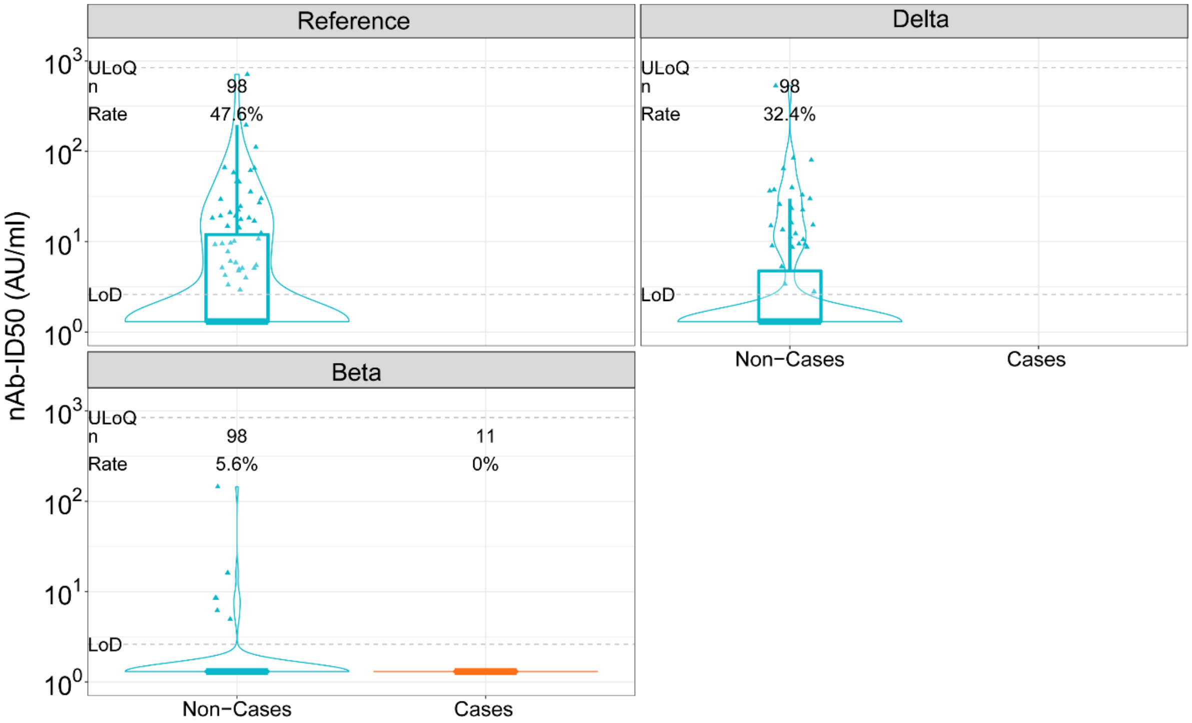

Figure S9. Violin plots of D29 nAb-ID50 in baseline SARS-CoV-2 seronegative per-protocol vaccine recipients in South Africa against each breakthrough case-matched lineage compared to against the same lineage in non-cases, Related to Figure 1. For every strain, original nAb-ID50 titers in AU/ml were multiplied by 0.0653 and thus nAb-ID50 Reference titers are equivalently expressed in international units (IU50)/ml. The limit of detection (LOD) was 2.612 AU/ml (= IU50/ml for Reference) and minimum upper limit of quantitation (ULOQ) used for truncating readouts was 844.7 AU/ml. n is number of participants with lineage-specific antibody data, Rate is response frequency.

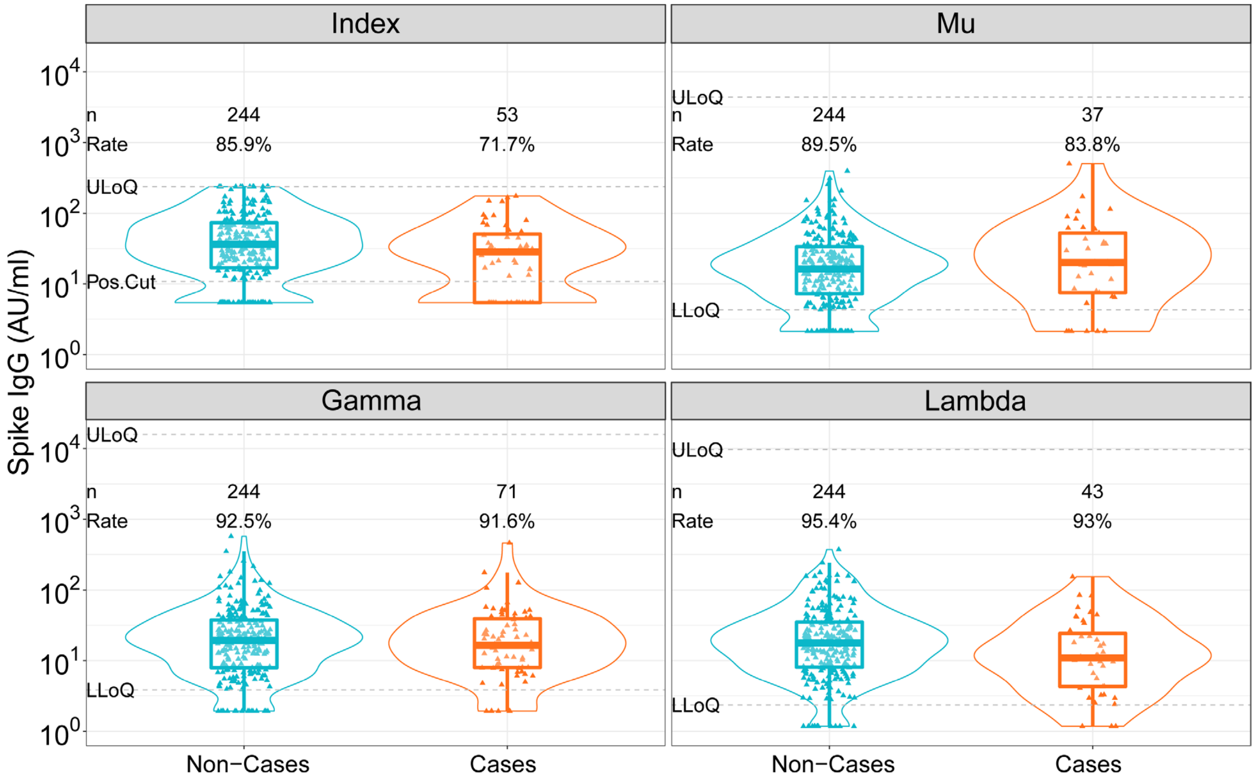

Figure S10. Violin plots of D29 Spike IgG in baseline SARS-CoV-2 seronegative per-protocol vaccine recipients in Latin America against each breakthrough case-matched lineage compared to against the same lineage in non-cases, Related to Figure 1. For every strain, all original Spike IgG readouts in AU/ml were multiplied by 0.009 and thus Spike IgG Index concentrations are equivalently expressed in binding antibody units (BAU)/ml. Pos.Cut, positivity cut-off (10.8424 AU/ml for Index). LLoQ, lower limit of quantification: Mu, 4.284 AU/ml; Gamma, 3.852 AU/ml; Lambda, 2.358 AU/ml. ULoQ, upper limit of quantification: Index, 238.1165 AU/ml; Mu, 4387.635 AU/ml; Gamma, 15788.42 AU/ml; Lambda, 9675.18 AU/ml. n is number of participants with lineage-specific antibody data, Rate is response frequency.

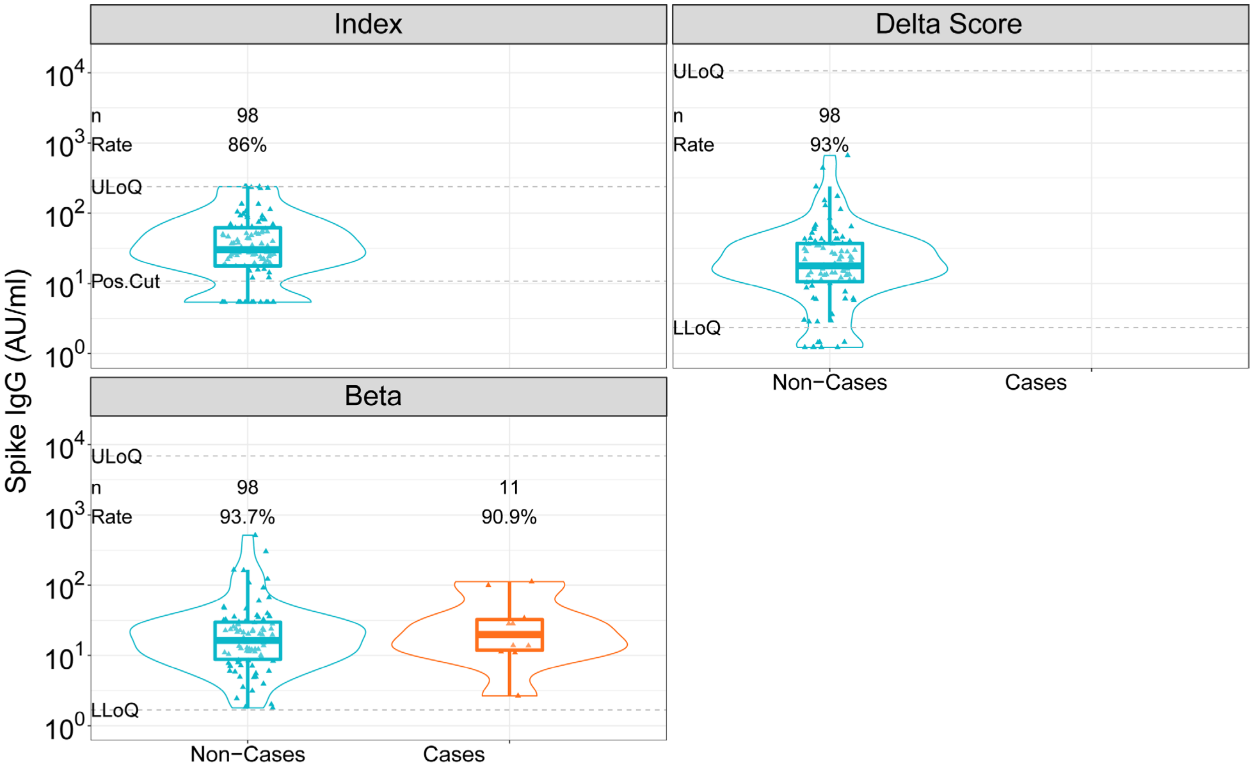

Figure S11. Violin plots of D29 Spike IgG in baseline SARS-CoV-2 seronegative per-protocol vaccine recipients in South Africa against each breakthrough case-matched lineage compared to against the same lineage in non-cases, Related to Figure 1. For every strain, all original Spike IgG readouts in AU/ml were multiplied by 0.009 and thus Spike IgG Index concentrations are equivalently expressed in binding antibody units (BAU)/ml. Pos.Cut, positivity cut-off (10.8424 AU/ml for Index). LLoQ, lower limit of quantification: Delta Score, 2.358 AU/ml; Beta, 1.683 AU/ml. ULoQ, upper limit of quantification: Index, 238.1165 AU/ml; Delta Score, 10572.41 AU/ml; Beta, 6889.743 AU/ml. n is number of participants with lineage-specific antibody data, Rate is response frequency.

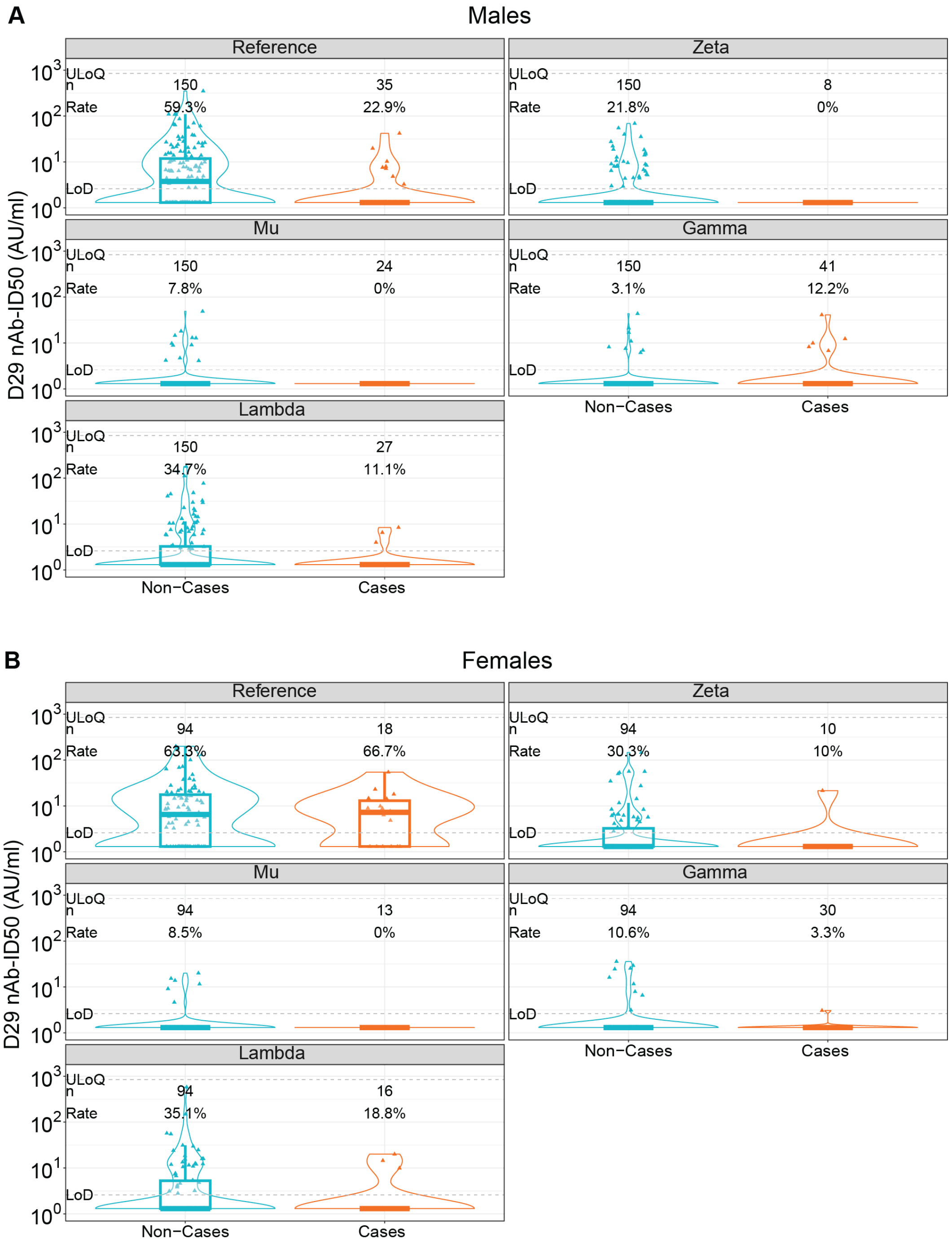

Figure S12. Violin plots of D29 nAb-ID50 in baseline SARS-CoV-2 seronegative per-protocol vaccine recipients in Latin America against each breakthrough case-matched lineage compared to against the same lineage in non-cases, shown separately in A) males and B) females, Related to Figure 1. This analysis was done post hoc. For every strain, original nAb-ID50 titers in AU/ml were multiplied by 0.0653 and thus nAb-ID50 Reference titers are equivalently expressed in international units (IU50)/ml. The limit of detection (LOD) was 2.612 AU/ml (= IU50/ml for Reference) and minimum upper limit of quantitation (ULOQ) used for truncating readouts was 844.7 AU/ml. n is number of participants with lineage-specific antibody data, Rate is response frequency. AU, arbitrary units; bAb, binding antibody; IU, international units; nAb-ID50, 50% inhibitory dilution neutralizing antibody titer.

A

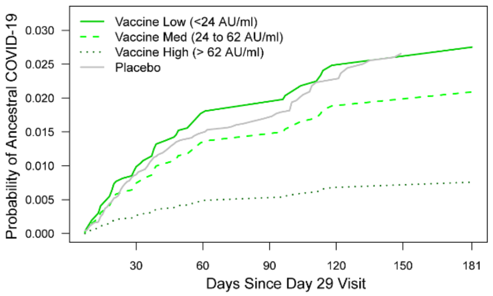

No. at risk						
Low:	2575	2156	1189	623	518	140
Med:	2431	2093	1410	545	434	233
High:	2573	2522	1767	837	687	358
Plac:	7352	6433	4290	1748	1229	558
**Cumulative No. of Ancestral COVID-19 Endpoints**						
Low:	14.4	22.2	23.9	29.6	29.8	30.8
Med:	6	17.4	19.1	21.5	21.5	21.5
High:	7.5	10.5	10.5	12.5	12.5	12.5
Plac:	66	110	123.9	146	155.7	156.1

B

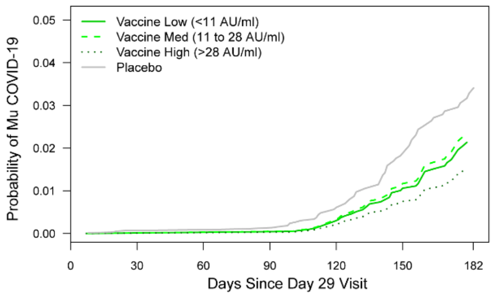

No. at risk						
Low:	2447.6	2316.1	1261.1	688.3	588.9	131.3
Med:	2587.5	2313.5	1766.2	505.6	399.6	64.2
High:	2567.1	2513.7	1634.7	904.1	667.5	388.5
Plac:	7352	6433	4290	1748	1229	534
**Cumulative No. of Mu COVID-19 Endpoints**						
Low:	0	0	0	1.6	7.9	13.5
Med:	0	0	0	3.8	7.1	9.3
High:	0	0	0	2.3	8.4	16.4
Plac:	5.3	5.6	8	23.3	47	67

C

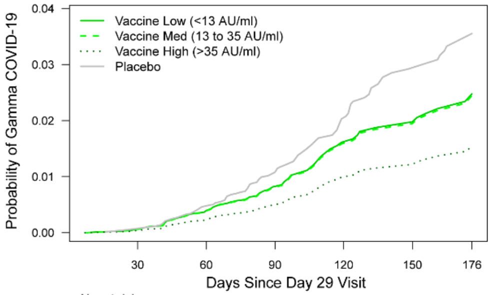

No. at risk						
Low:	2473.6	2343.2	1318.2	701.7	600.6	391
Med:	2526.4	2214.9	1476.7	519.6	388.9	146.5
High:	2602.6	2585.9	1867.1	876.7	666.5	440.5
Plac:	7352	6433	4290	1748	1229	702
**Cumulative No. of Gamma COVID-19 Endpoints**						
Low:	1.3	12.6	22.9	29.6	31.9	31.9
Med:	1.6	9.9	18.4	22.9	24	27.5
High:	0.7	4.7	8.1	16.5	18.7	20.7
Plac:	5.3	32.7	68.5	96.6	113.2	119.7

D

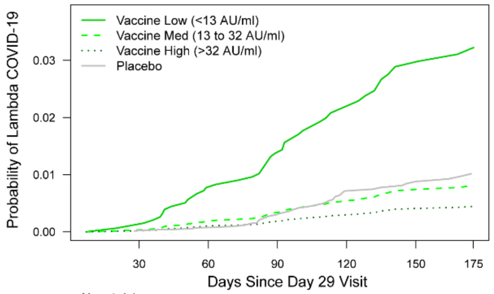

No. at risk						
Low:	2469.1	2346.6	1364.3	772	645.7	397.9
Med:	2602.8	2285.8	1369.3	537.1	455.5	252.3
High:	2531	2510.6	1928.4	789	554.8	361.8
Plac:	7352	6433	4290	1748	1229	736
**Cumulative No. of Lambda COVID-19 Endpoints**						
Low:	0.2	5.5	13.6	21.3	26	28.8
Med:	1.4	4.7	4.9	7.2	9.3	9.3
High:	0.3	4.4	6.5	7.5	9.5	10.5
Plac:	2	5	20	38	43	46.1

E

D29 Marker	COVID-19 Outcome	D29 Marker Tertile	Hazard Ratio Pt. Est.	Hazard Ratio 95% CI	P-value (2-sided)	Overall P-value
Spike IgG Index	Ancestral	Low	1	N/A	N/A	0.023
		Med	0.69	(0.34, 1.39)	0.298	
		High	0.32	(0.14, 0.72)	0.006	
Spike IgG Mu	Mu	Low	1	N/A	N/A	0.754
		Med	1.08	(0.33, 3.53)	0.900	
		High	0.68	(0.18, 2.54)	0.562	
Spike IgG Gamma	Gamma	Low	1	N/A	N/A	0.360
		Med	0.98	(0.48, 2.01)	0.952	
		High	0.60	(0.28, 1.31)	0.199	
Spike IgG Lambda	Lambda	Low	1	N/A	N/A	0.004
		Med	0.24	(0.08, 0.69)	0.008	
		High	0.13	(0.03, 0.51)	0.004	

Figure S13. A-D) Covariate-adjusted cumulative incidence of lineage-matched COVID-19 by Low, Medium or High tertile of D29 lineage-specific Spike IgG concentration in baseline SARS-CoV-2 seronegative per-protocol Latin America vaccine recipients, Related to Figure 3. A) Spike IgG Index, Ancestral COVID-19; B) Spike IgG Mu, Mu COVID-19; C) Spike IgG Gamma, Gamma COVID-19; D) Spike IgG Lambda, Lambda COVID-19; E) Estimated hazard ratios of lineage-matched COVID-19 for the Medium versus Low and for the High versus Low tertiles of D29 lineage-specific Spike IgG concentration. The overall P value is from a generalized Wald test of whether the hazard rate of lineage-matched COVID-19 differed across the Low, Medium, and High subgroups. Analyses adjusted for baseline risk score. For every strain, all original Spike IgG readouts in AU/ml were multiplied by 0.009, and thus in (A), Spike IgG Index cut-points separating Low vs. Medium vs. High in AU/ml are equivalently expressed in binding antibody units (BAU)/ml. AU, arbitrary unit; CI, confidence interval; Pt. Est., point estimate.

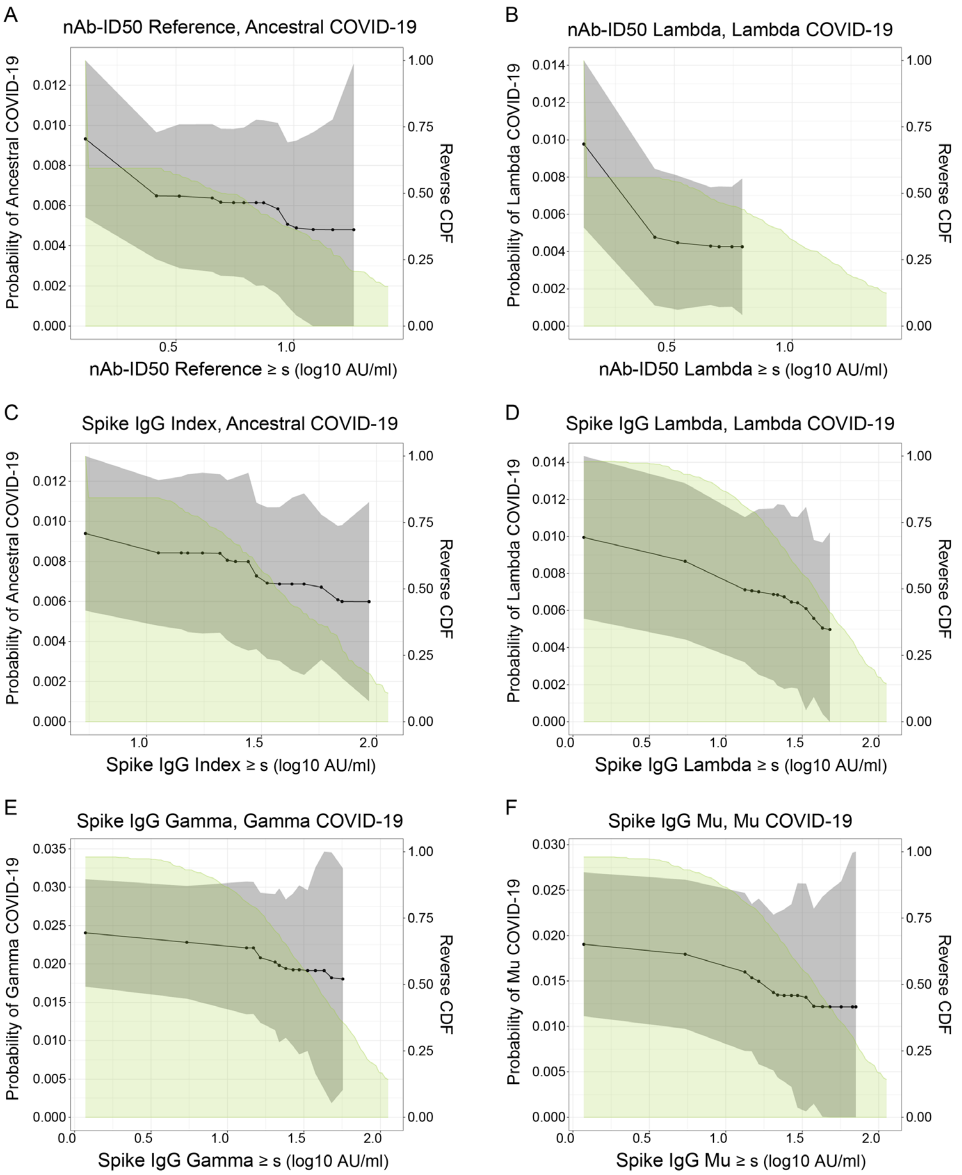

Figure S14. Cumulative incidence of lineage-specific COVID-19 by D29 lineage-matched nAb-ID50 titer above a threshold or by D29 lineage-matched Spike IgG concentration above a threshold for Latin America circulating lineages and vaccine recipients, Related to Figure 5. Incidence measured through 220 days post-vaccination. Dots represent point estimates at given threshold values. For every strain, original nAb-ID50 titers in AU/ml were multiplied by 0.0653 and thus nAb-ID50 Reference titers are equivalently expressed in international units (IU50)/ml; for every strain, all original Spike IgG readouts in AU/ml were multiplied by 0.009 and thus Spike IgG Index concentrations are equivalently expressed in binding antibody units (BAU)/ml. To define the set of dots/thresholds for reporting of point estimates for the nAb-ID50 assay [(A) and (B)], first a common grid of thresholds was obtained by quantile binning the D29 nAb-ID50 Reference titer values at COVID-19 endpoints (including all endpoints regardless of lineage) into 20 equal-frequency bins. Then, for each lineage of COVID-19, black dots at which fewer than 5 lineage-specific COVID-19 endpoints had lineage-matched nAb-ID50 titers above the dot value were excluded. For (C)-(F), the same process was applied based on D29 Spike IgG Index values and on lineage-matched D29 Spike IgG values. The grey shaded area indicates pointwise 95% CIs. The estimates and CIs were adjusted using the assumption that the true threshold-response is non-increasing. The upper boundary of the green shaded area is the estimate of the reverse cumulative distribution function (CDF) of D29 antibody marker level. Analyses adjusted for baseline behavioral risk score.

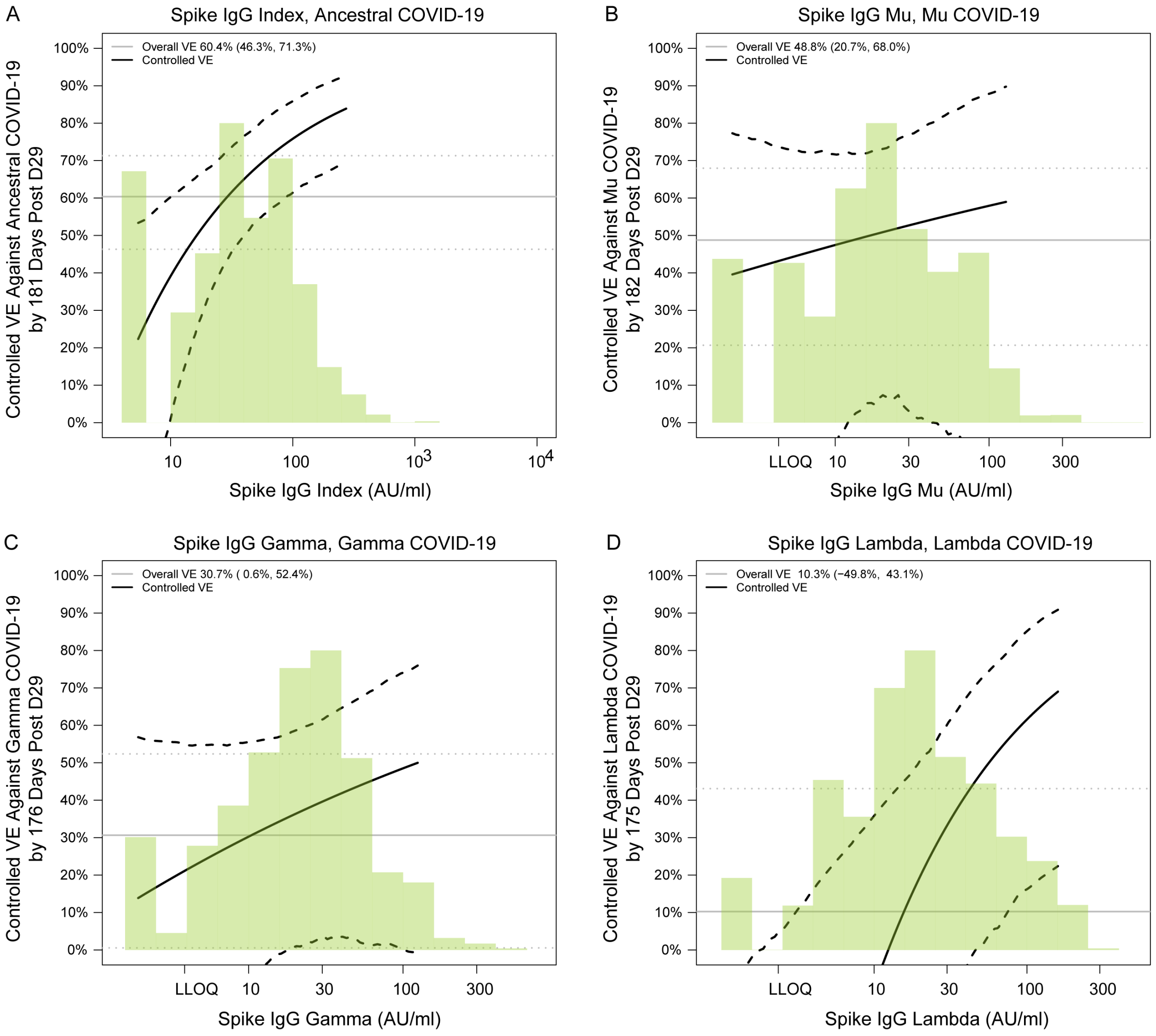

Figure S15. Lineage-matched controlled vaccine efficacy (CVE) curves in baseline SARS-CoV-2 seronegative per-protocol participants in Latin America, Related to Figure 4. Solid lines are point estimates and dashed lines are 95% pointwise confidence intervals. Curves are plotted ranging from the LLOQ/2 to the 97.5th percentile of Spike IgG. For every strain, all original Spike IgG readouts in AU/ml were multiplied by 0.009 and thus Spike IgG Index concentrations are equivalently expressed in binding antibody units (BAU)/ml. AU, arbitrary unit.

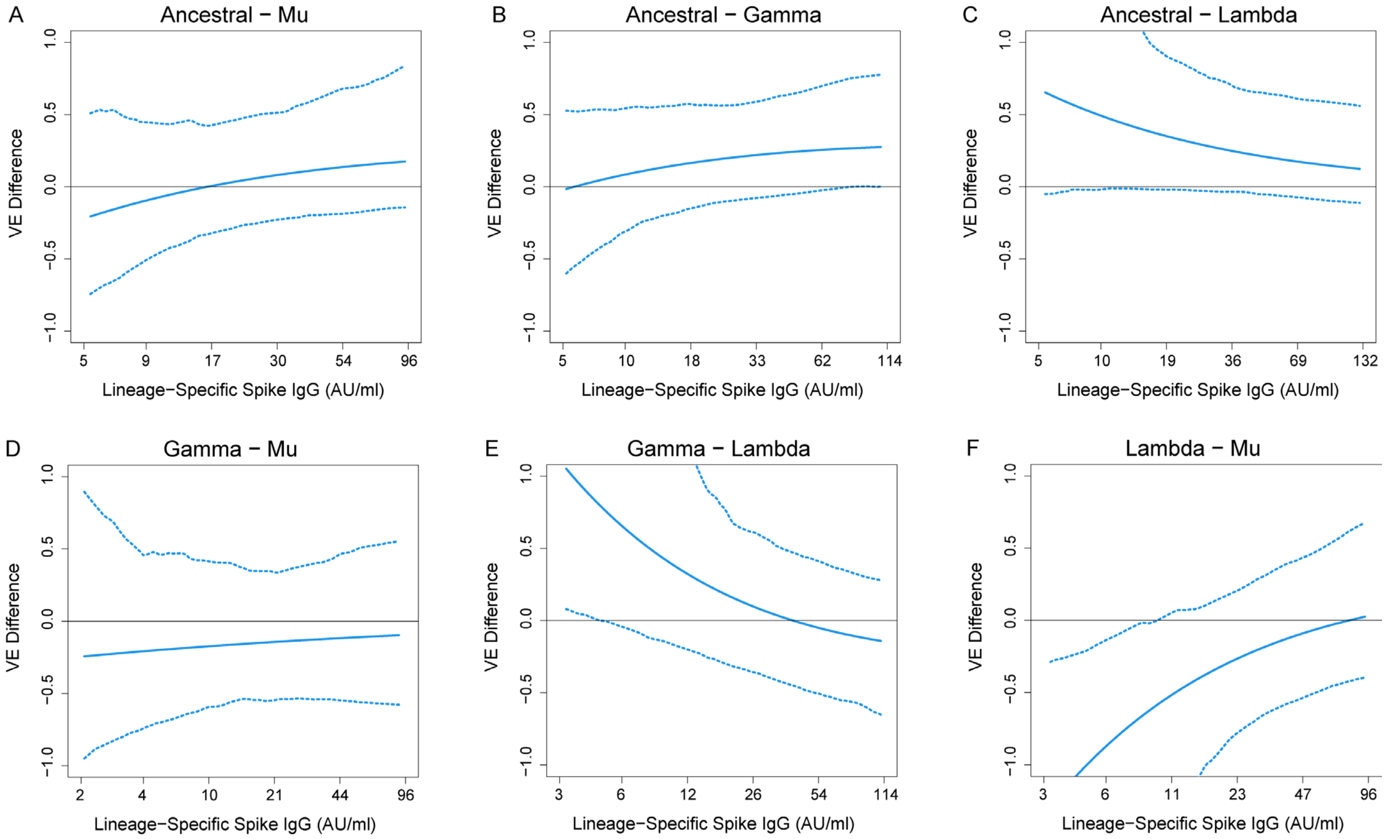

Figure S16. Controlled vaccine efficacy (CVE) difference curves for D29 lineage-matched Spike IgG in baseline SARS-CoV-2 seronegative per-protocol participants in Latin America, Related to Figure 4. Each solid blue line shows the scaled difference between two different curves of lineage-specific CVE by lineage-specific D29 Spike IgG concentration, calculated as: log10{CVEcontrast(s)} = log10{1- CVE-(lineage-specific COVID-19)(s)} – log10{1 – CVE-(a different lineage-specific COVID-19)(s)}. The dashed lines are 95% pointwise confidence intervals. Curves are plotted ranging from the LLOQ/2 to the 97.5th percentile of Spike IgG. For every strain, all original Spike IgG readouts in AU/ml were multiplied by 0.009 and thus Spike IgG Index concentrations are equivalently expressed in binding antibody units (BAU)/ml. AU, arbitrary unit.

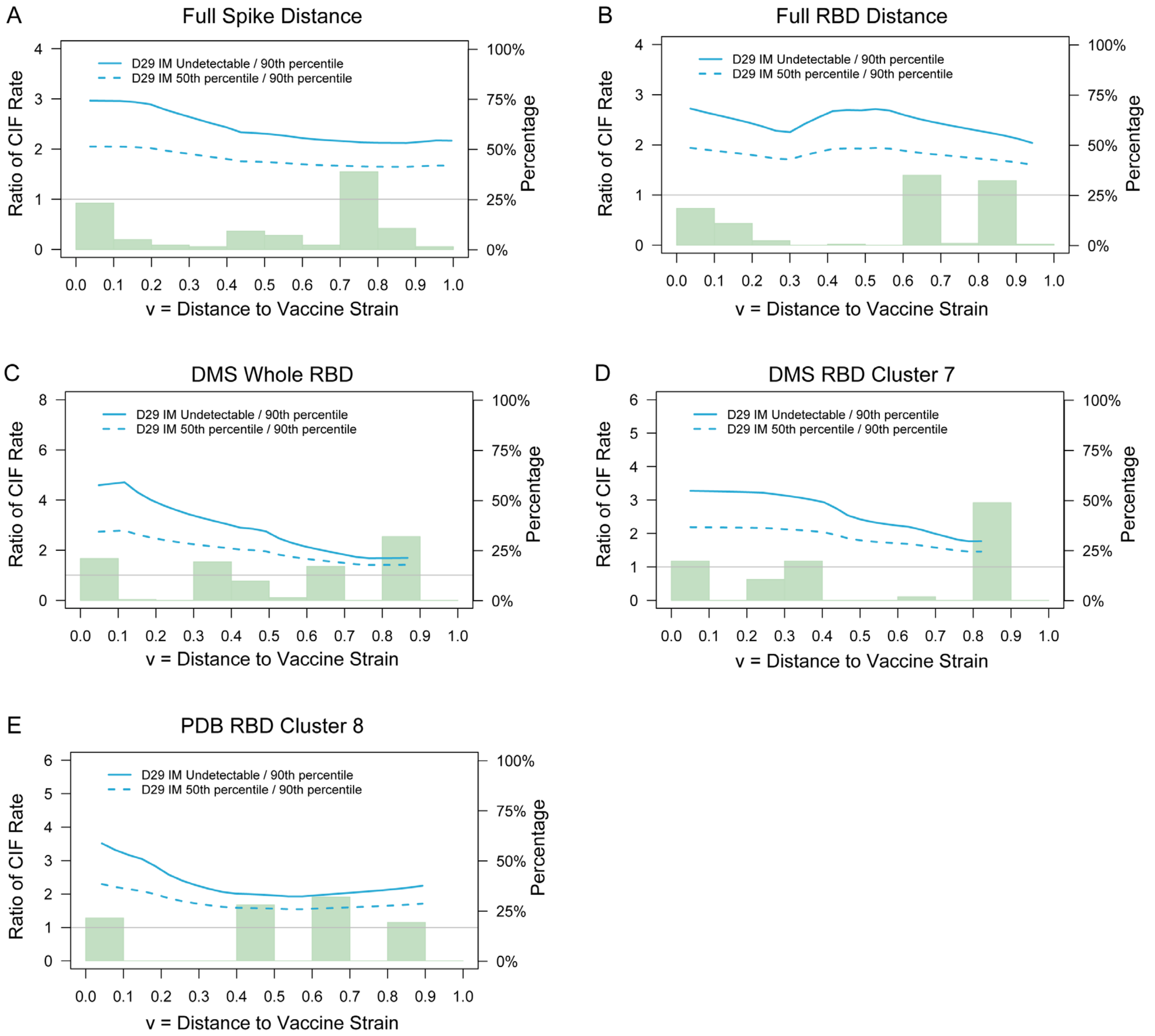

Figure S17. Cumulative incidence analysis of the dependency of SARS-CoV-2 amino acid sequence distance-specific COVID-19 risk on D29 nAb-ID50 Reference titer in baseline seronegative per-protocol vaccine recipients in Latin America, Related to Figure 6. Each panel shows the distance-specific cumulative incidence failure rate ratio for Medium vs. Low and High vs. Low values of D29 nAb-ID50 Reference titer (Low: undetectable = 1.31 AU/ml; Medium: 50th percentile = 6.27 AU/ml; High: 90th percentile = 37.8 AU/ml). Distances assessed are A) weighted Spike Hamming distance; B) weighted RBD Hamming distance; C) DMS whole-RBD escape score; D) DMS cluster 7-RBD escape score; E) PDB8 cluster 8-RBD escape score. Distances are from COVID-19 endpoint sequences to the vaccine-strain sequence and are defined in Magaret et al. [1]. Shaded regions are 95% pointwise confidence intervals. Histograms of the distances are in green (right y-axis).

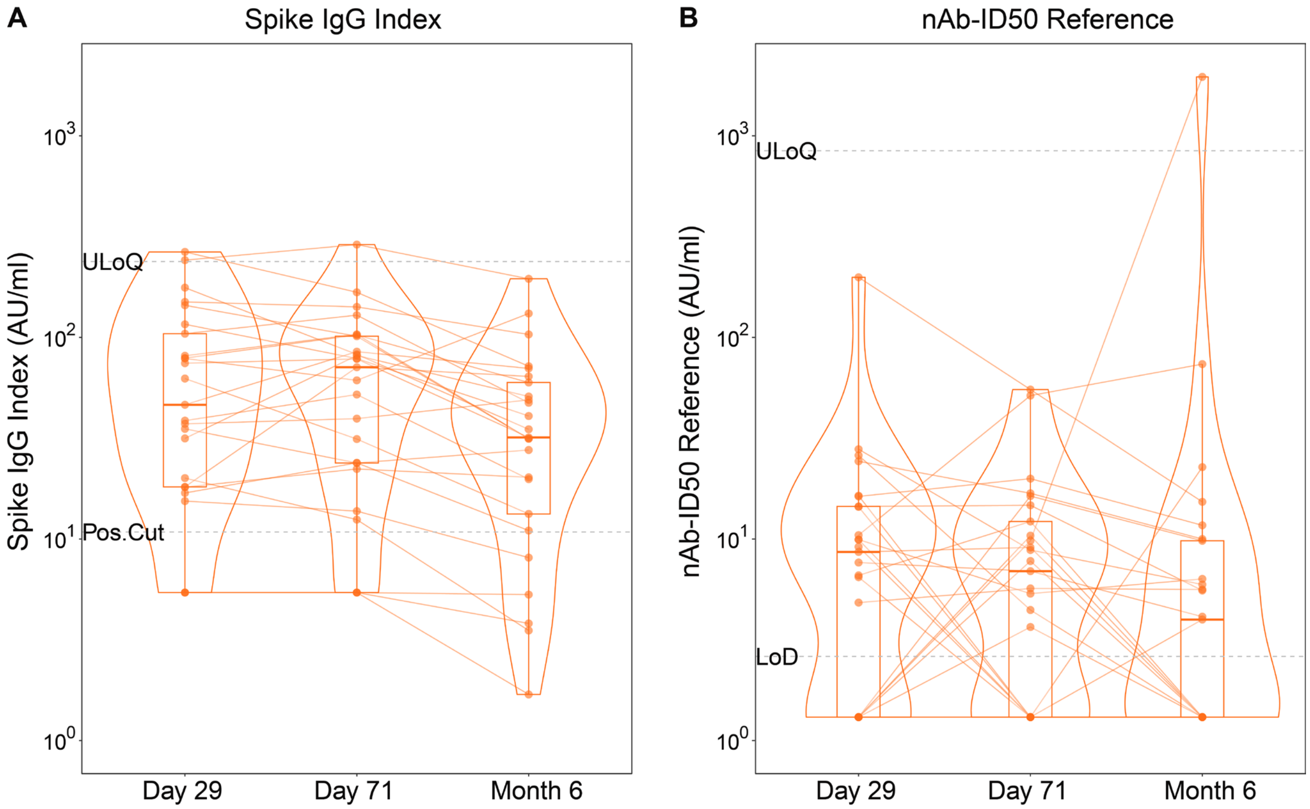

Figure S18. For a random sample of 25 per-protocol baseline SARS-CoV-2 seronegative vaccine recipients without acquisition of the moderate-to-severe COVID-19 primary endpoint through to the end of the blinded phase, A) anti-Spike IgG concentration (AU/ml) against Index and B) 50% inhibitory dilution neutralizing antibody titer (AU/ml, nAb-ID50) against Reference measurements at Day 29 (D29), Day 71 (D71), and Month 6 (M6) are shown. The 25 participants were randomly sampled from among eligible participants that had both Spike IgG Index and nAb-ID50 Reference measurements at all three time points D29, D71, and M6. These plots are shown as examples of D29 to M6 antibody trajectories; D29, D71, and M6 antibody data were used in the exposure-proximal correlates analysis as described in the STAR Methods. Each line connects the D29, D71, and M6 measurements from a single participant. Original nAb-ID50 titers in AU/ml were multiplied by 0.0653 and thus nAb-ID50 Reference titers are equivalently expressed in international units (IU50)/ml; original Spike IgG readouts in AU/ml were multiplied by 0.009 and thus Spike IgG Index concentrations are equivalently expressed in binding antibody units (BAU)/ml. AU, arbitrary units; LOD, lower limit of detection; Pos.Cut, positivity cutoff; ULOQ, upper limit of quantitation.

Table S1. Day 29 nAb-ID50 and Spike IgG detectable/positive response frequencies and geometric means by lineage-matched breakthrough cases vs. non-cases in South Africa and the United States (Per-Protocol Baseline Seronegative Vaccine Recipients), Related to Table 1.

			**Lineage-Matched COVID-19 Cases**			**Non-Cases**			**Comparison**	
Geographic Region	D29 Marker	Lineage	N	Response Frequency	GMC (AU/ml)	NC	Response Frequency	GMC (AU/ml)	Resp frequency difference (Cases – Non-Cases) (95% CI)	Ratio of GMC (Cases/Non-Cases) (95% CI)
South Africa	nAb-ID50	Reference	-	-	-	113	45.8% (35.0%, 57.0%)	4.41 (3.13, 6.21)	-	-
South Africa	nAb-ID50	Beta	11	0.0% (0.0%, 0.0%)	1.31 (1.31, 1.31)	100	5.5% (2.2%, 13.1%)	1.49 (1.31, 1.68)	-0.055 (-0.131, -0.022)	0.88 (0.78, 1.00)
United States	nAb-ID50	Reference	37	40.5% (25.7%, 57.4%)	2.86 (2.05, 3.98)	429	51.8% (46.1%, 57.5%)	4.05 (3.52, 4.66)	-0.113 (-0.272, 0.065)	0.70 (0.49, 1.01)
South Africa	Spike IgG	Index	-	-	-	113	83.2% (74.1%, 89.5%)	33.9 (26.3, 43.7)	-	-
South Africa	Spike IgG	Delta*	-	-	-	98	93.0% (85.7%, 96.7%)	20.9 (15.7, 27.8)	-	-
South Africa	Spike IgG	Beta	-	-	-	100	93.9% (86.8%, 97.3%)	15.6 (11.7, 20.8)	-	-
South Africa	Spike IgG	Mu	-	-	-	110	88.0% (80.0%, 93.0%)	16.3 (12.4, 21.4)	-	-
South Africa	Spike IgG	Gamma	-	-	-	110	90.8% (84.2%, 94.8%)	18.3 (13.9, 23.9)	-	-
South Africa	Spike IgG	Lambda	-	-	-	110	92.3% (86.3%, 95.9%)	16.9 (12.7, 22.5)	-	-
United States	Spike IgG	Index	37	83.5% (67.2%, 92.6%)	27.5 (20.4, 36.9)	429	84.5% (79.8%, 88.3%)	33.9 (30.0, 38.4)	-0.01 (-0.178, 0.092)	0.81 (0.59, 1.11)

Case = Moderate-to-severe COVID-19 primary endpoint that occurred starting 7 days post D29 through to the end of the blinded phase (140 days for South Africa and the United States). N is the number of vaccine recipient breakthrough cases caused by the indicated lineage with D29 marker data against the lineage. Non-case = No acquisition of the moderate-to-severe COVID-19 primary endpoint through to the end of the blinded phase. NC is the number of Non-cases with D29 marker data against the lineage. For nAb-ID50, response frequency is the estimated percentage with nAb-ID50 above the lower detection limit (LOD) = 2.612 AU/ml (= IU50/ml for Reference). For Spike IgG, response frequency is the estimated percentage with IgG above the minimum lower limit of quantitation (LLOQ) = 1.683 AU/ml (= BAU/ml for Index). GMT is the estimated geometric mean titer of nAb-ID50 and GMC is the estimated geometric mean concentration of Spike IgG. For every strain, original nAb-ID50 titers in AU/ml were multiplied by 0.0653 and thus nAb-ID50 Reference titers (but not any of the variant nAb titers) are equivalently expressed in international units (IU50)/ml; for every strain, all original Spike IgG readouts in AU/ml were multiplied by 0.009 and thus Spike IgG Index concentrations (but not any of the variant Spike IgG concentrations) are equivalently expressed in binding antibody units (BAU)/ml.
*Response frequencies and GMCs are for Delta-score (maximal signal diversity weighted average readouts to the five Delta strains in MSD Panel 19).

Table S2. Classification of six statistical analyses conducted for assessing Day 29 antibody markers as correlates of risk and correlates of protection specific to the SARS-CoV-2 lineage or Spike amino acid sequence distance to the vaccine-strain at a specific Day 29 antibody marker value (D29 marker) or for subgroups defined by a range of D29 marker values. All analyses adjust for baseline risk score, Related to STAR Methods.

<table>
<tr><th></th><th></th><th>Correlate of Risk Analyses Conducted
(Vaccine arm Only)</th><th>Correlate of Protection Analyses Conducted
[Vaccine and Placebo arms – involving vaccine efficacy (VE)]</th></tr>
<tr><td rowspan="3">Virus Characteristic: SARS-CoV-2 Spike feature defined by Lineage or Distance to vaccine-strain

Host Antibody Characteristic: D29 marker subgroup defined by a Specific value or Range of values</td><td>Viral lineage

Specific marker value</td><td>• Cox models: Hazard ratios of lineage-specific COVID-19 per 10-fold increase in D29 marker value (Figure 2, Table S3, Table S4)</td><td>• Cox models: Cumulative VE against lineage-specific COVID-19 at specific vaccinee D29 marker values (Figure 4, Figure S15).
• Based on the above, compare two lineages by the ratios of one minus cumulative VEs (Figure 4, Figure S16).</td></tr>
<tr><td>Viral lineage

Range of marker values</td><td>• Cox models: Hazard ratios of lineage-specific COVID-19 for D29 marker tertile subgroups Medium vs. Low and High vs. Low (Figure 3, Figure S13)
• Nonparametric models: Cumulative incidence of lineage-specific COVID-19 for subgroups defined by D29 marker value above a threshold (Figure S14)</td><td>• Nonparametric models: Cumulative VE against lineage-specific COVID-19 for subgroups defined by vaccinee D29 marker values above a threshold (Figure 5)</td></tr>
<tr><td>Spike distance to vaccine-strain

Specific marker value</td><td>• Cox models: Hazard ratios of distance-specific COVID-19 per 10-fold increase in D29 marker value (Figure 6, Table S5)
• Cox models: Ratios of distance-specific COVID-19 cumulative incidence rate function comparing two subgroups defined by specific D29 marker values (Figure S17).</td><td>• Cox models: Cumulative VE against distance-specific COVID-19 at specific vaccinee D29 marker values (Figure 7)</td></tr>
</table>

Table S3. Post hoc analysis of hazard ratio point estimates and 95% confidence intervals of lineage-specific COVID-19 per 10-fold increase in D29 lineage-specific nAb-ID50 or nAb-ID50 Reference in baseline SARS-CoV-2 seronegative per-protocol A) male or B) female vaccine recipients in Latin America, Related to Figure 2. Hazard ratios were estimated using a Cox model with adjustment for baseline risk score. For every strain, original nAb-ID50 titers in AU/ml were multiplied by 0.0653 and thus nAb-ID50 Reference titers are equivalently expressed in international units (IU50)/ml; for every strain, all original Spike IgG readouts in AU/ml were multiplied by 0.009 and thus Spike IgG Index concentrations are equivalently expressed in binding antibody units (BAU)/ml. AU, arbitrary unit; nAb-ID50, 50% inhibitory dilution neutralizing antibody titer. No p values are presented since this analysis was not prespecified.

**A. Males**	
**D29 Marker (COVID-19 Outcome)**	**Hazard Ratio Point Estimate (95% CI)**
nAb-ID50 Reference (Ancestral COVID-19)	0.57 (0.27, 1.23)
nAb-ID50 Lambda (Lambda COVID-19)	0.06 (0.01, 0.45)
nAb-ID50 Reference (Lambda COVID-19)	0.25 (0.07, 0.83)
Spike IgG Index (Ancestral COVID-19)	0.99 (0.42, 2.36)
Spike IgG Mu (Mu COVID-19)	0.08 (0.00, 2.78)
Spike IgG Index (Mu COVID-19)	0.33 (0.07, 1.49)
Spike IgG Gamma (Gamma COVID-19)	0.20 (0.06, 0.70)
Spike IgG Index (Gamma COVID-19)	0.93 (0.38, 2.29)
Spike IgG Lambda (Lambda COVID-19)	0.04 (0.01, 0.12)
Spike IgG Index (Lambda COVID-19)	0.29 (0.08, 0.98)
**B. Females**	
**D29 Marker (COVID-19 Outcome)**	**Hazard Ratio Point Estimate (95% CI)**
nAb-ID50 Reference (Ancestral COVID-19)	0.23 (0.09, 0.61)
nAb-ID50 Lambda (Lambda COVID-19)	0.10 (0.02, 0.43)
nAb-ID50 Reference (Lambda COVID-19)	0.48 (0.17, 1.34)
Spike IgG Index (Ancestral COVID-19)	0.22 (0.09, 0.52)
Spike IgG Mu (Mu COVID-19)	1.17 (0.34, 4.08)
Spike IgG Index (Mu COVID-19)	1.46 (0.35, 6.05)
Spike IgG Gamma (Gamma COVID-19)	0.84 (0.36, 1.98)
Spike IgG Index (Gamma COVID-19)	1.15 (0.48, 2.75)
Spike IgG Lambda (Lambda COVID-19)	0.46 (0.17, 1.27)
Spike IgG Index (Lambda COVID-19)	0.35 (0.12, 1.04)

Table S4. Comparison of hazard ratios of lineage-specific COVID-19 per 10-fold increase in D29 lineage-specific nAb-ID50 vs. D29 lineage-specific Spike IgG (in vaccine recipients), shown for Latin America, Related to Figure 2. Binding antibody and neutralizing antibody measurements are expressed in AU/ml. Hazard ratios were estimated using multivariable Cox models including a pair of nAb-ID50 and Spike IgG variables against the same lineage with adjustment for baseline risk score.

	D29 Marker (AU/ml)	Lineage-Specific COVID-19	HR per 10-Fold Increase Pt. Est. (95% CI)	P value
**Latin America**				
	nAb-ID50 Reference	Ancestral	0.42 (0.22, 0.80)	0.009
	Spike IgG Index		0.64 (0.32, 1.29)	0.210
Generalized Wald Test				0.002
	nAb-ID50 Reference	Lambda	0.49 (0.19, 1.28)	0.147
	Spike IgG Index		0.53 (0.19, 1.43)	0.207
Generalized Wald Test				0.007
	nAb-ID50 Lambda	Lambda	0.13 (0.03, 0.50)	0.003
	Spike IgG Lambda		0.74 (0.29, 1.87)	0.520
Generalized Wald Test				0.001

Table S5. Hypothesis tests for whether the Spike-distance specific D29 nAb-ID50 hazard ratio of COVID-19 in SARS-CoV-2 baseline seronegative per-protocol vaccine recipients attenuates toward one with increasing Spike protein distance to the vaccine-insert sequence*, Related to Figure 6.

Day 29 Antibody Marker	Spike Amino Acid Sequence Distance	P-value
nAb-ID50 Reference Strain	Spike weighted Hamming distance	0.29
nAb-ID50 Reference Strain	RBD weighted Hamming distance	0.45
nAb-ID50 Reference Strain	DMS whole-RBD escape score	0.074
nAb-ID50 Reference Strain	DMS cluster 7-RBD escape score	0.19
nAb-ID50 Reference Strain	PDB cluster 8-RBD escape score	0.30

*The Spike amino acid sequence distances are defined in Magaret et al. [1], all of which correlated with vaccine efficacy against moderate to severe-critical COVID-19 with FWER $p < 0.05$. DMS = deep mutation scanning experiments that defined neutralization-relevant RBD features, with escape scores defined for whole-RBD and for each of 10 epitope-specific clusters of amino acid sites, labeled DMS (whole-RBD) and DMS1 through DMS10 in Magaret et al. PDB = Protein Data Bank derived putative antibody footprint site sets on structures of SARS-CoV-2 in complex with antibodies available from the PDB. Escape scores were defined based on a class of epitopes referred to as PDB1 through PDB14, with the first 12 clusters in the RBD and PDB13 and PDB14 in the NTD.

Table S6. Assay limits of the V-PLEX SARS-CoV-2 Panel 19 (IgG) Kit IgG binding antibody assay, Related to STAR Methods.

SARS-CoV-2 Spike antigen	Positivity Cutoff (AU/ml)	LLOQ (AU/ml)	ULOQ (AU/ml)
D614/Index	10.8424*	1.683*	238.1165*
Spike Delta Score	2.358	2.358	10572.41
Spike Beta (B.1.351)	1.683	1.683	6889.743
Spike Mu (B.1.621)	4.284	4.284	4387.635
Spike Gamma (P.1)	3.852	3.852	15788.42
Spike Lambda (C.37)	2.358	2.358	9675.18

*For D614/Index, the original assay readouts in arbitrary units/ml (AU/ml) were multiplied by 0.009 such that AU/ml = 20/136 D614/Index strain WHO International Unit Scale BAU/ml. Original assay readouts in AU/ml for the other 9 antigens were also multiplied by 0.009.

Antigen-specific LLOQs and ULOQs are used, with values less than the LLOQ assigned to LLOQ/2 and values above the ULOQ set to the ULOQ. However, the Spike Index antigen was an exception, with values less than the positivity cutoff (Pos.Cut) assigned to Pos.Cut/2.

Supplemental References

1. Magaret CA, Li L, deCamp AC, Rolland M, Juraska M, Williamson BD, et al. Quantifying how single dose Ad26.COV2.S vaccine efficacy depends on Spike sequence features. Nat Commun. 2024;15(1):2175. Epub 2024/03/12. doi: 10.1038/s41467-024-46536-w. PubMed PMID: 38467646; PubMed Central PMCID: PMCPMC10928100.
