# Supplementary material for: Immune correlates analysis of antibody responses against SARS-CoV-2 variants in the ENSEMBLE vaccine efficacy trial
Source: iScience. 2025 Sep 29;28(11):113660. doi: 10.1016/j.isci.2025.113660 (PMC12589887; doi:10.1016/j.isci.2025.113660)
Supplement: Data S1. PDF with names and affiliations of the Immune Assays Team, the Coronavirus Vaccine Prevention Network (CoVPN)/ENSEMBLE Team, and the United States Government (USG)/CoVPN Biostatistics Team [file mmc2.pdf]

Immune Assays Team

Affiliation	Team Members
Biomedical Advanced Research and Development Authority (BARDA), Washington, DC	Oleg Borisov, Flora Castellino, Brett Chromy, Mark Delvecchio, Ruben O. Donis, Tremel Faison, Corey Hoffman, Christopher Houchens, Tom Hu, Pennie Hylton, Lakshmi Jayashankar, Aparna Kolhekar, James Little, Karen Martins, Jeanne Novak, Azhar Ravji, Carol Sabourin, Evan Sturtevant, Kimberly Taylor, Xiaomi Tong, John Treanor, Danielle Turley, Leah Watson, Daniel Wolfe
Boston Consulting Group, Boston, MA	Gian King, Andrew Li, Najaf Shah, Smruthi Suryaprakash, Jue Xiang Wang
Division of AIDS, NIAID, NIH, Bethesda, MD	Patricia D'Souza
Division of MID (Microbiology and Infectious Diseases), NIAID, NIH, Bethesda, MD	Janie Russell
Duke University, Durham, NC	David Beaumont, Kendall Bradley, Jiayu Chen, Xiaoju Daniell, Thomas Denny, Elizabeth Domin, Amanda Eaton, Kelsey Engel, Wenhong Feng, Juanfei Gao, Hongmei Gao, Kelli Greene, Sarah Hiles, Leihua Liu, Kristy Long, Kellen Lund, Charlene McDanal, David C. Montefiori, Marcella Sarzotti- Kelsoe, Francesca Suman, Haili Tang, Jin Tong, Olivia Widman
LabCorp-Monogram Biosciences, South San Francisco, CA, USA	Christos J. Petropoulos, Terri Wrin
The Tauri Group, an LMI company - Contract Support for U.S. Department of Defense (DOD) Joint Program Executive Office for Chemical, Biological, Radiological and Nuclear Defense (JPEO-CBRND) Joint Project Manager for Chemical, Biological, Radiological, and Nuclear Medical (JPM CBRN Medical), Fort Detrick, Maryland, USA	Christopher S. Badorrek, Gregory E. Rutkowski
Vaccine Research Center, NIAID, NIH, Bethesda, MD	Obrimpong Amoa-Awua, Manjula Basappa, Robin Carroll, Britta Flach, Suprabhath Gajjala, Nazaire Jean-Baptiste, Richard A. Koup, Bob C. Lin, Adrian McDermott, Christopher Moore, Mursal Naisan, Muhammed Naqvi, Sandeep Narpala, Sarah O’Connell, Clare Whittaker, Weiwei Wu, Allen Mueller, Martin Apgar, Tommy Bruington, Joe Stashick, Leo Serebryannyy, Mike Castro, Jennifer Wang

Coronavirus Vaccine Prevention Network (CoVPN)/ENSEMBLE Team

Study Group Member	Affiliation	Location
Jerald Sadoff, M.D.	Janssen Vaccines and Prevention	Leiden, the Netherlands
Glenda Gray, M.B., B.Ch.	South African Research Council	Cape Town, South Africa
An Vandebosch, Ph.D.	Janssen Research and Development	Beerse, Belgium
Vicky Cárdenas, Ph.D.	Janssen Research and Development	Spring House, PA, USA
Georgi Shukarev, M.D.	Janssen Vaccines and Prevention	Leiden, the Netherlands
Beatriz Grinsztejn, M.D.	Evandro Chagas National Institute of Infectious Diseases–Fiocruz	Rio de Janeiro, Brazil
Paul A. Goepfert, M.D.	University of Alabama at Birmingham	Birmingham, AL, USA
Carla Truyers, Ph.D.	Janssen Research and Development	Beerse, Belgium
Hein Fennema, Ph.D.	Janssen Research and Development	Beerse, Belgium
Bart Spiessens, Ph.D.	Janssen Research and Development	Beerse, Belgium
Kim Offergeld, M.Sc.	Janssen Research and Development	Beerse, Belgium
Gert Scheper, Ph.D.	Janssen Vaccines and Prevention	Leiden, the Netherlands
Kimberly L. Taylor, Ph.D.	National Institute of Allergy and Infectious Diseases	Rockville, MD, USA
Merlin L. Robb, M.D.	Walter Reed Army Institute of Research	Silver Spring, MD, USA
John Treanor, M.D.	Biomedical Advanced Research and Development Authority	Washington, DC, USA
Dan H. Barouch, M.D.	Center for Virology and Vaccine Research, Beth Israel Deaconess Medical Center	Boston, MA, USA
Jeffrey Stoddard, M.D.	Janssen Research and Development	Raritan, NJ, USA
Martin F. Ryser, M.D.	Janssen Research and Development	Beerse, Belgium
Mary A. Marovich, M.D.	National Institute of Allergy and Infectious Diseases	Rockville, MD, USA
Kathleen M. Neuzil, M.D.	Center for Vaccine Development and Global Health, University of Maryland School of Medicine	Baltimore, MD, USA
Lawrence Corey, M.D.	Vaccine and Infectious Disease Division, Fred Hutchinson Cancer Research Center	Seattle, WA, USA
Nancy Cauwenberghs, Ph.D.	Janssen Research and Development	Beerse, Belgium

Tamzin Tanner, Ph.D.	Janssen Research and Development	Beerse, Belgium
Karin Hardt, Ph.D.	Janssen Research and Development	Beerse, Belgium
Javier Ruiz-Guiñazú, M.D.	Janssen Research and Development	Beerse, Belgium
Mathieu Le Gars, Ph.D.	Janssen Vaccines and Prevention	Leiden, the Netherlands
Hanneke Schuitemaker, Ph.D.	Janssen Vaccines and Prevention	Leiden, the Netherlands
Johan Van Hoof, M.D.	Janssen Vaccines and Prevention	Leiden, the Netherlands
Frank Struyf, M.D.	Janssen Research and Development	Beerse, Belgium
Macaya Douoguih, M.D.	Janssen Vaccines and Prevention	Leiden, the Netherlands
Richard Gorman, MD	Biomedical Advanced Research andDevelopment Authority (BARDA)	Washington, DC, USA
Carmen A. Paez, MD, MBA	Fred Hutchinson Cancer Research Center	Seattle, WA, USA
Edith Swann, PhD	National Institute of Allergy and Infectious Diseases (NIAID)	Rockville, MD, USA
James Kublin, MD, MPH	Fred Hutchinson Cancer Research Center	Seattle, WA, USA
Simbarashe G. Takuva, MBChB,MSc	Fred Hutchinson Cancer Research Center;University of the Witwatersrand; University of Pretoria	Seattle, WA, USA; Johannesburg, South Africa; Pretoria, South Africa
Alex Greninger, MD, PhD, MS, MPhil	University of Washington; Fred Hutchinson Cancer Research Center	Seattle, WA, USA
Pavitra Roychoudhury, PhD	University of Washington; Fred Hutchinson Cancer Research Center	Seattle, WA, USA
Robert W. Coombs, MD, PhD	University of Washington	Seattle, WA, USA
Keith R. Jerome, MD, PhD	University of Washington; Fred Hutchinson Cancer Research Center	Seattle, WA, USA
Flora Castellino, MD	Biomedical Advanced Research andDevelopment Authority (BARDA)	Washington, DC, USA
Xiaomi Tong, PhD	Biomedical Advanced Research andDevelopment Authority (BARDA)	Washington, DC, USA
Corrina Pavetto, MS, RAC	Biomedical Advanced Research andDevelopment Authority (BARDA)	Washington, DC, USA
Teletha Gipson, PhD, MS	Biomedical Advanced Research andDevelopment Authority (BARDA)	Washington, DC, USA

Tina Tong, DrPH(c), MS,RAC(US)	National Institute of Allergy and Infectious Diseases (NIH/NIAID)	Rockville, MD, USA
Marina Lee, PhD	National Institute of Allergy and Infectious Diseases (NIH/NIAID)	Rockville, MD, USA
James Zhou, PhD, MS	Biomedical Advanced Research andDevelopment Authority (BARDA)	Washington, DC, USA
Michael Fay, PhD	National Institute of Allergy and Infectious Diseases (NIH/NIAID)	Rockville, MD, USA
Kelly McQuarrie, BSN	Janssen Research & Development	Horsham, PA, USA
Chimeremma Nnadi, MD, PhD	Janssen Pharmaceuticals	Titusville, NJ, USA
Obiageli Sogbetun, MD, MPH	Janssen Infectious Disease and Vaccines	Titusville, NJ, USA
Nina Ahmad, MD	Janssen Pharmaceuticals	Titusville, NJ, USA
Ian De Proost, PhD	Johnson & Johnson	Beerse, Belgium
Cyrus Hoseyni, PhD	Janssen Research & Development	Spring House, PA, USA
Paul Coplan, ScD, MS, MBA	Johnson & Johnson Epidemiology	New Brunswick, NJ, USA
Najat Khan, PhD	Janssen Research & Development	New Brunswick, NJ, USA
Peter Ronco, BA	Janssen Research & Development	Raritan, NJ, USA
Dawn Furey, BA	Janssen Research & Development	Titusville, NJ, USA
Jodi Meck, MHA	Janssen Research & Development	Titusville, NJ, USA
Johan Vingerhoets, PhD	Janssen Pharmaceutica NV	Beerse, Belgium
Boerries Brandenburg, PhD	Janssen Vaccines & Prevention B.V.	Leiden, The Netherlands
Jerome Custers, PhD	Janssen Vaccines & Prevention B.V.	Leiden, The Netherlands
Jenny Hendriks, PhD	Janssen Vaccines & Prevention B.V.	Leiden, The Netherlands
Jarek Juraszek, PhD	Janssen Vaccines & Prevention B.V.	Leiden, The Netherlands
Anne Marit de Groot, PhD	Janssen Vaccines & Prevention B.V.	Leiden, The Netherlands
Griet Van Roey, PhD	Janssen Vaccines & Prevention B.V.	Leiden, The Netherlands
Dirk Heerwegh, PhD	Janssen Research & Development; Janssen	Beerse, Belgium
Ilse Van Dromme, PhD	Johnson & Johnson	Beerse, Belgium

CoVPN/ENSEMBLE Team, Cont'd: Participating Investigators
The following Principal Investigators participated in the ENSEMBLE study:

Name	Institute	Location
Aberg, Judith	Icahn School of Medicine at Mount Sinai	New York, NY, USA
Adams, Mark	Central Kentucky Research Associates, Inc.	Lexington, KY, USA
Adams, Michael	Synexus Clinical Research US, Inc.	Murray, UT, USA
Aguayo, Samuel	VA Medical Center	Phoenix, AZ, USA
Ahsan, Habibul	The University of Chicago Medicine	Chicago, IL, USA
Aizenberg, Diego	Centro Médico Viamonte SRL	Buenos Aires, Argentina
Alonto, Augusto	Jesse Brown VAMC Department of Surgery	Chicago, IL, USA
Alzogaray, Maria Fernanda	Instituto Médico Platense	Buenos Aires, Argentina
Anderson, Evan	Emory University School of Medicine	Atlanta, GA, USA
Andrade Pinto, Jorge	Hospital das Clínicas da Universidade Federal de Minas Gerais	Belo Horizonte, Brazil
Arns da Cunha, Clóvis	Hospital Nossa Senhora das Graças	Paraná, Brazil
Avelino Silva, Vivian	Hospital das Clínicas da Faculdade de Medicina da USP	São Paulo, Brazil
Badal-Faesen, Sharlaa	University of Witwatersrand – Helen Joseph Hospital – Themba Lethu HIV Research Centre	Johannesburg, South Africa
Baden, Lindsey	Brigham and Women's Hospital, Inc.	Boston, MA, USA
Barnabas, Shaun	Family Clinical Research Unit FAM-CRU	Western Cape, South Africa
Bedimo, Roger	AIDS Arms Incorporated Trinity Health and Wellness Center	Dallas, TX, USA
Bekker, Linda-Gail	Desmond Tutu HIV Centre, University of Cape Town	Cape Town, South Africa
Berhe, Mezgebe	North Texas Infectious Diseases Consultants	Dallas, TX, USA
Bessesen, Mary	Rocky Mountain Regional VA Medical Center	Aurora, CO, USA
Blaser, Martin J	Rutgers Robert Wood Johnson Medical School	New Brunswick, NJ, USA
Bonvehi, Pablo Eduardo	CEMIC Saavedra	Buenos Aires, Argentina
Borger, Judith	Carolina Institute for Clinical Research	Fayetteville, NC, USA
Brites Alves, Carlos Roberto	Fundação Bahiana de Infectologia	Bahia, Brazil
Brown, Sheldon	Bronx Veterans Affairs Medical Center	Bronx, NY, USA
Brumskine, William	The Aurum Institute Rustenburg Clinical Research Centre	Rustenburg, South Africa
Brune, Daniel	Optimal Research, LLC	Peoria, IL, USA
Buynak, Robert	Buynak Clinical Research	Valparaiso, IN, USA
Cabrera May, Carlos Antonio	Unidad de Atención Médica e Investigación en Salud (UNAMIS)	Yucatán, Mexico

Cadena Bonfanti, Andres Angelo	Clínica de la Costa	Barranquilla, Colombia
Cahn, Pedro	Fundación Huésped	Buenos Aires, Argentina
Carson, Jeffrey L.	Rutgers Robert Wood Johnson Medical School	New Brunswick, NJ, USA
Casapia Morales, Wilfredo Martin	Asociación Civil Selva Amazónica (ACSA)	Loreto, Peru
Cassetti, Lidia Isabel	Helios Salud S.A.	Buenos Aires, Argentina
Castex, Julie	Ochsner Medical Center	New Orleans, LA, USA
Chu, Laurence	Benchmark Research	Austin, TX, USA
Cotugno, Michael	Benchmark Research	Metairie, LA, USA
Creech, Clarence	Vanderbilt University Medical Center	Nashville, TN, USA
Crofoot, Gordon	CrofootMD Clinic and Research Center	Houston, TX, USA
Curtis, Brian	Corvallis Clinic PC	Corvallis, OR USA
da Silva Pilotto, José Henrique	Hospital Geral de Nova Igauçu	Rio de Janeiro, Brazil
Dal Ben Corradi, Mirian de Freitas	Hospital Sírio-Libanês	São Paulo, Brazil
Dal Pizzol, Felipe	Hospital São José	Santa Catarina, Brazil
Davis, Matthew	Rochester Clinical Research, Inc.	Rochester, NY, USA
De Carvalho Santana, Rodrigo	Hospital das Clínicas da Faculdade de Medicina de Ribeirão Preto daUSP	São Paulo, Brazil
de Faria Freire, Antônio Tarcísio	Santa Casa de Misericórdia de Belo Horizonte	Belo Horizonte, Brazil
DeJesus, Edwin	Orlando Immunology Center	Orlando, FL, USA
Delafontaine, Patrice	New Orleans Adolescent Trials Unit CRS	New Orleans, LA, USA
Delano Bronstein, Marcello	CPQuali Pesquisa Clínica LTDA ME	São Paulo, Brazil
Dell'Italia, Louis	University of Alabama at Birmingham	Birmingham, AL, USA

Deluca, Mercedes	Clinical Trials Division – Stamboulian Servicios de Salud	Buenos Aires, Argentina
Denham, Douglas	Clinical Trials of Texas, Inc.	San Antonio, TX, USA
Diacon, Andreas	TASK Central	Western Cape, South Africa
Dubula, Thozama	Nelson Mandela Academic Clinical Research Unit (NeMACRU)	Mthatha, South Africa
Eder, Frank	Meridian Clinical Research, LLC	Endwell, NY, USA
Edupuganti, Srilatha	The Hope Clinic at Emory University	Decatur, GA, USA
Ervin, John	The Center for Pharmaceutical Research	Kansas City, MO, USA
Esteves Coelho, Lara	FIO CRUZ – Fundação Oswaldo Cruz – Inst de Pesquisa ClínicaEvandro Chagas	Rio de Janeiro, Brazil
Eudes Leal, Fabio	Universidade Municipal de São Caetano do Sul	São Paulo, Brazil
Fairlie, Lee	Shandukani Research Centre	Gauteng, South Africa
Fierro, Carlos	Johnson County Clin-Trials	Lenexa, KS, USA

Fogarty, Charles	Spartanburg Medical Research	Spartanburg, SC, USA
Fragoso, Veronica	Texas Center for Drug Development, Inc.	Houston, TX, USA
Frank, Ian	University of Pennsylvania	Philadelphia, PA, USA
Frey, Sharon	Saint Louis University	St Louis, MO, USA
Gallardo Cartagena, Jorge	Centro de Investigaciones Tecnológicas, Biomédicas y Medioambientales (CITBM)	Lima, Peru
Gamarra Ayarza, Cesar Augusto	Centro de Investigaciones Médicas	Callao, Peru
Garcia Diaz, Julia	Ochsner Medical Center	New Orleans, LA, USA
Gaur, Aditya	St Jude Children’s Research Hospital	Memphis, TN, USA
Gentile, Nina	Temple University Hospital	Philadelphia, PA, USA
Gill, Katherine	Masiphumelele Research Centre	Cape Town, South Africa
Gonzalez, Alexander	MedPlus Medicina Prepagadá S.A.	Bogotá, Colombia
Gottlieb, Robert	Baylor Scott & White Research Institute	Dallas, TX, USA
Grant, Philip	Stanford University Medical Center	Palo Alto, CA, USA
Greenberg, Richard	University of Kentucky	Lexington, KY, USA
Greiwe, Cathy	Synexus Clinical Research US, Inc.	Columbus, OH, USA

Guedes Barbosa, Luiz Sergio	Oncovida – Centro de Onco-Hematologia de Mato Grosso	Mato Grosso, Brazil
Han-Conrad, Laurie	WR-MCCR, LCC	San Diego, CA, USA
Hidalgo Vidal, Jose Alfredo	Asociación Civil Vía Libre	Lima, Peru
Higuera Cobos, Juan Diego	Fundación Oftalmológica de Santander – FOSCAL	Santander, Colombia
Hong, Matthew	Wake Research Associates	Raleigh, NJ, USA
Innes, James Craig	The Aurum Institute Klerksdorp Clinical Research Centre	Klerksdorp, South Africa
Jackson, Lisa	Kaiser Permanente Washington Health Research Institute	Seattle, WA, USA
Jackson-Booth, Peta-Gay	Optimal Research, LLC	Rockville, MD, USA
Jaller Raad, Juan Jose	Centro de Reumatología y Ortopedia	Barranquilla, Colombia
Jayaweera, Dushyantha	University of Miami – Miller School of Medicine	Miami, FL, USA
Jennings, William	Synexus Clinical Research US, Inc.	San Antonio, TX, USA
João Filho, Esaú Custódio	Hospital Federal dos Servidores do Estado	Rio de Janeiro, Brazil
Kassim, Sheetal	Desmond Tutu HIV Foundation – University of Cape Town	Cape Town, South Africa
Kennelly, Christina	Tryon Medical Partners	Charlotte, NC, USA
Khetan, Shishir	Meridian Clinical Research, LLC	Rockville, MD, USA
Kilgore, Paul E.	Henry Ford Health System	Detroit, MI, USA
Kim, Kenneth	Ark Clinical Research	Long Beach, CA, USA
Kirby, William	Synexus Clinical Research US, Inc.	Birmingham, AL, USA

Kopp, James	Synexus Clinical Research US, Inc.	Anderson, SC, USA
Kotze, Philip	Qhakaza Mbokodo Research Clinic	KwaZulu-Natal, South Africa
Kotze, Sheena	Stanza Clinical Research Centre: Mamelodi	Gauteng, South Africa
Kriesel, John	University of Utah	Salt Lake City, UT, USA
Kutner, Mark	Suncoast Research Group	Miami, FL, USA
Lacerda Nogueira, Maurício	Fundação Faculdade Regional de Medicina de São José do Rio Preto	São Paulo, Brazil
Laher, Fatima	Perinatal HIV Research Unit, Chris Hani Baragwanath Hospital	Gauteng, South Africa
Lama Valdivia, Javier Ricardo	Asociación Civil Impacta Salud y Educación – Barranco	Lima, Peru
Lazarus, Erica	Perinatal HIV Research Unit (PHRU), Kliptown	Soweto, South Africa

Lazcano Ponce, Eduardo Cesar	Instituto Nacional de Salud Pública	Morelos, Mexico
Leibman, Daniel	VA Medical Center	Columbia, SC, USA
Levin, Michael	Clinical Research Center of Nevada	Las Vegas, NV, USA
Levin, Myron	Children’s Hospital Colorado	Aurora, CO, USA
Little, Susan	UCSD AntiViral Research Center (AVRC)	San Diego, CA, USA
Lombaard, Johannes	Josha Research	Bloemfontein, South Africa
Lopez Medina, Eduardo	Centro de Investigaciones Clínicas S.A.S.	Cali, Colombia
Losso, Marcelo	Hospital J.M. Ramos Mejía	Buenos Aires, Argentina
Luabeya, Angelique	SATVI, Brewelskloof Hospital	Western Cape, South Africa
Lucksinger, Gregg	Clinical Research Institute of Southern Oregon, P.C.	Medford, OR, USA
Lugogo, Njira	University of Michigan Neurosurgery A. Alfred Taubman Health CareCenter	Ann Arbor, MI, USA
Luz, Kleber Giovanni	Centro de Estudos e Pesquisas em Moléstias Infecciosas	Rio Grande do Norte, Brazil
Maboa, Rebone	Ndlovu Elandsdoorn Site	Limpopo, Dennilton, South Africa
Macareno Arroyo, Hugo Andres	Hospital Universidad del Norte	Barranquilla, Colombia
Makhaza, Disebo	CAPRISA Vulindlela Clinic	KwaZulu-Natal, South Africa
Malahleha, Mookho	Setshaba Research Centre	Soshanguve, South Africa
Malan, Daniel	PHOENIX Pharma (Pty) Ltd.	Eastern Cape, South Africa
Mamba, Musawenkosi	CRISMO Bertha Gxowa Research Centre	Gauteng, South Africa
Manning, Mary Beth	Rapid Medical Research	Cleveland, OH, USA
Martin, Judith	University of Pittsburgh	Pittsburgh, PA, USA
Mauricio da Silva, Cesar	Faculdade de Medicina Barretos – FACISB	Barretos, SP, Brazil

McGettigan, John	Quality of Life Medical & Research Center, LLC	Tucson, AZ, USA
Medrano Allende, Juan	Clínica y Maternidad Suizo Argentina	Buenos Aires, Argentina
Mena, Leandro	University of Mississippi Medical Center	Jackson, MS, USA
Messer, William	Oregon Health & Science University	Portland, OR, USA
Middleton, Randle	Optimal Research, LLC	Huntsville, AL, USA
Mills, Anthony	Anthony Mills Medical, Inc.	Los Angeles, CA, USA
Mills, Richard	PMG Research of Charleston, LLC	Mount Pleasant, SC, USA
Mngadi, Kathryn	The Aurum Institute: Tembisa – Clinic 4	Tembisa, South Africa
Moanna, Abeer	VA Medical Center – Atlanta	Decatur, GA, USA
Mofsen, Ricky	Massachusetts General Hospital	Boston, MA, USA
Moncada Vilela, Zandra	Hospital Nacional Arzobispo Loayza	Lima, Peru
Montaña, Oscar Romano	DIM Clínica Privada	Buenos Aires, Argentina
Moreno Hoyos Abril, Juan	Hospital Universitario de Nuevo León ‘Dr. José Eleuterio González’	Nuevo León, Mexico
Morse, Caryn	Wake Forest Baptist Medical Center	Winston-Salem, NC, USA
Muñoz Reyes, Manuel	Hospital Dr. Hernán Henríquez Aravena	Araucanía, Chile
Murray, Linda	Synexus Clinical Research US, Inc.	Pinellas Park, FL, USA
Naicker, Nivashnee	Centre for the AIDS Programme of Research in South Africa	KwaZulu-Natal, South Africa
Naicker, Vimla	South Africa Medical Research Council	KwaZulu-Natal, South Africa
Naidoo, Logashvari	South African Medical Research Council Chatsworth Clinical ResearchSite	KwaZulu-Natal, South Africa
Nchabeleng, Maphoshane	MeCRU Clinical Research Unit	Gauteng, South Africa
Newman Lobato Souza, Tamara	Instituto de Infectologia Emilio Ribas	São Paulo, Brazil
Novak, Richard	University of Illinois at Chicago	Chicago, IL, USA
Nugent, Paul	Synexus Clinical Research US, Inc.	Cincinnati, OH, USA
O'Ryan Gallardo, Miguel Luis	Facultad de Medicina, Universidad de Chile	Santiago, Chile
Oyanguren Miranda, Martin	Hospital Nacional Edgardo Rebagliati Martins	Lima, Peru
Padala, Kalpana	Central Arkansas Veterans Healthcare System	Little Rock, AR, USA
Panettieri, Jr., Reynold A.	Rutgers Robert Wood Johnson Medical School	New Brunswick, NJ, USA
Panjwani, Sameer G.	Rush University Medical Center	Chicago, IL, USA
Patelli Juliani Souza Lima, Maria	Hospital e Maternidade Celso Pierro	São Paulo, Brazil
Pelkey, Leslie	Cherry Street Services, Inc.	Grand Rapids, MI, USA
Petrick, Friedrich	Mzansi Ethical Research Centre	Middelburg, South Africa
Pounds, Kevin	Synexus Clinical Research US, Inc.	Tucson, AZ, USA

Powell, Richard	New Horizons Clinical Research	Cincinnati, OH, USA
Pragalos, Antoinette	CTI Clinical Trial and Consulting Services	Cincinnati, OH, USA
Pratley, Richard E.	AdventHealth Orlando	Orlando, FL, USA
Presti, Rachel	Washington University School of Medicine	St Louis, MO, USA
Ramalho Madruga, José Valdez	Centro de Referência e Treinamento DST/AIDS	São Paulo, Brazil
Ramesh, Mayur	Henry Ford Health System	Detroit, MI, USA
Ramirez Sanchez, Isabel Cristina	Hospital Pablo Tobón Uribe	Antioquia, Colombia
Ramirez, Julio	University of Louisville	Louisville, KY, USA
Rankin, Bruce	Avail Clinical Research, LLC	DeLand, FL, USA
Restrepo, Jaime	Fundación Centro de Investigación Clinída (CIC)	Medellín, Colombia
Reynales Londono, Humberto	Centro de Atención e Investigación Médica S.A. – CAIMED	Bogotá, Colombia
Reynolds, Michele	Synexus Clinical Research US, Inc.	Dallas, TX, USA
Rhame, Frank	Abbott Northwestern Hospital Clinic	Minneapolis, MN, USA
Rhee, Margaret	Synexus Clinical Research US, Inc.	Akron, OH, USA
Riddle, Mark	VA Sierra Nevada Health Care System	Reno, NV, USA
Riegel Santos, Breno	Hospital Nossa Senhora da Conceição (HNSC)	Rio Grande do Sul, Brazil
Riffer, Ernie	Central Phoenix Medical Clinic	Phoenix, AZ, USA
Rizzardi, Barbara	Advanced Clinical Research	West Jordan, UT, USA
Rosso, Fernando	Fundación Valle del Lili	Valle del Cauca, Colombia
Saavedra, Carla	Bioclinica Santiago Bulnes	Santiago, Chile
Safirstein, Beth	MD Clinical	Hallandale Beach, FL, USA
Scapellato, Pablo	CEMEDIC	Buenos Aires, Argentina
Scheinberg, Phillip	Real e Benemérita Associação Portuguesa de Beneficência	São Paulo, Brazil
Schwartz, Howard	Research Centers of America, LLC	Hollywood, FL, USA
Sedillo, David J.	Rush University Medical Center	Chicago, IL, USA
Servilla, Karen	Raymond G. Murphy VA Medical Center	Albuquerque, NM, USA
Shah, Raj	Rush University Medical Center	Chicago, IL, USA
Siegel, Amy	MediSync Clinical Research	Petal, MS, USA
Silva Orellana, Rafael	Clínica del Maule	Talca, Chile
Silva, Federico	Fundación Cardiovascular de Colombia – Instituto del CorazónFloridablanca	Floridablanca, Colombia
Smith, Steven R.	AdventHealth Orlando	Orlando, FL, USA
Spooner, Elizabeth	South Africa Medical Research Council	KwaZulu-Natal, South Africa
Sprinz, Eduardo	Hospital de Clínicas de Porto Alegre	Porto Alegre, Brazil

Sriram, Peruvemba	North Florida/South Georgia Veterans Health System	Gainesville, FL, USA
Strout, Cynthia	Coastal Carolina Research Center, Inc.	Mount Pleasant, SC, USA
Swiatlo, Edwin	Southeast Louisiana Veterans Health Care System	New Orleans, LA, USA
Taiwo, Babafemi	Northwestern University	Evanston, IL, USA
Talwani, Rohit	Baltimore VA Medical Center	Baltimore, MD, USA
Tavares Russo, Luís Augusto	Instituto Brasil de Pesquisa Clínica	Rio de Janeiro, Brazil
Terront Lozano, Monica Alexandra	Solano y Terront Servicios Médicos Ltda	Bogotá, Colombia
Tharenos, Leslie	Synexus Clinical Research US, Inc.	St Louis, MO, USA
Tien, Phyllis	VA Medical Center	San Francisco, CA, USA
Tieu, Hong Van	New York Blood Center	New York, NY, USA
Toney, John	James A. Haley VA Hospital GNS	Tampa, FL, USA
Urbach, Dorothea	Synexus Helderberg Clinical Research Centre	Western Cape, South Africa
Vachris, Timothy	Optimal Research, LLC	Austin, TX, USA
Valencia, Javier	Asociación Civil Impacta Salud y Educación – San Miguel CRS	Lima, Peru
van Nieuwenhuizen, Elane	Synexus Watermeyer	Gauteng, South Africa
Vannucci Lomonte, Andrea	CEPIC – Centro Paulista de Investigação Clínica	São Paulo, Brazil
Vasconcellos, Eduardo	Instituto de Pesquisas Clínicas	Distrito Federal, Brazil
Velez, Ivan Dario	Programa de Estudio y Control de Enfermedades Tropicales	Medellín, Colombia
Ward, Amy	University of Cape Town IDM/CIDRI Research Site	Cape Town, South Africa
Wells, Cassia	Harlem Hospital Center	New York, NY, USA
White, Judith	Synexus Clinical Research US, Inc.	Orlando, FL, USA
Winkle, Peter	Anaheim Clinical Trials, LLC	Anaheim, CA, USA
Woods, Christopher	Durham VAMC	Raleigh, NC, USA
Zaidman, Cesar Javier	CIPREC	Buenos Aires, Argentina

United States Government (USG)/Coronavirus Prevention Network (CoVPN) Biostatistics Team
(PubMed listed, and ordered alphabetically by institution affiliation)

Affiliation	Team Members
Biomedical Advanced Research and Development Authority (BARDA), Washington, DC	Di Lu, James Zhou
Department of Biostatistics and Bioinformatics, Rollins School of Public Health, Emory University	David Benkeser
Vaccine and Infectious Disease Division, Fred Hutchinson Cancer Center, Seattle, WA	Jessica Andriesen, Bhavesh Borate, Lindsay N. Carpp, Andrew Fiore-Gartland, Youyi Fong*, Peter B. Gilbert*, Ying Huang*, Yunda Huang, Ollivier Hyrien, Holly E. Janes*, Michal Juraska, Yiwen Lu, April K. Randhawa, Lars W.P. van der Laan, Chenchen Yu, Bo Zhang
Biostatistics Research Branch, NIAID, NIH, Bethesda, MD	Michael P. Fay, Jonathan Fintzi, Dean Follmann, Martha Nason
Clinical Monitoring Research Program Directorate, Frederick National Laboratory for Cancer Research, Frederick, MD	Eric Chu
Department of Biostatistics, T.H. Chan School of Public Health, Harvard University	Nima S. Hejazi
Department of Biostatistics, University of Washington, Seattle, WA	Marco Carone, James Peng, Charlotte Talham
Department of Statistics, University of Washington, Seattle, WA	Alex Luedtke
Department of Population Health Sciences, Weill Cornell Medical College, New York, New York	Iván Díaz
Kaiser Permanente Washington Health Research Institute	Brian D. Williamson
Department of Biostatistics and Bioinformatics, Duke University; Global Health Institute, Duke University	Avi Kenny

*YF, PBG, YiH, and HEJ are also affiliated with the Department of Biostatistics, University of Washington, Seattle, WA. PBG and YiH are also affiliated with the Public Health Sciences Division, Fred Hutchinson Cancer Center, Seattle, WA.
